# The Development of the DePaul Symptom Questionnaire: Original, Expanded, Brief, and Pediatric Versions

**DOI:** 10.3389/fped.2018.00330

**Published:** 2018-11-06

**Authors:** Leonard A. Jason, Madison Sunnquist

**Affiliations:** Center for Community Research, DePaul University, Chicago, IL, United States

**Keywords:** myalgic encephalomyelitis, chronic fatigue syndrome, case definition, DePaul Symptom Questionnaire, instrument development

## Abstract

One of the key requirements of a reliable case definition is the use of standardized procedures for assessing symptoms. This article chronicles the development of the DePaul Symptom Questionnaire (DSQ) to assess symptoms of the major chronic fatigue syndrome (CFS) and myalgic encephalomyelitis (ME) case definitions. The original questionnaire has been modified and expanded over time to more fully capture symptoms from various adult case definitions, and a brief as well as pediatric version have also been developed. The DSQ has demonstrated very good psychometric properties in terms of test-retest reliability and sensitivity/specificity, as well as construct, predictive, and discriminant validity. The DSQ allows for a clear characterization of a patient's illness and allows scientists and clinicians to improve diagnostic reliability and validity when employing case definitions of ME and CFS.

Since 1994, many researchers have used the Fukuda et al. (1) chronic fatigue syndrome (CFS) case definition to select cases, but problems emerged in part due to this case definition not requiring core symptoms of CFS (2). In contrast, myalgic encephalomyelitis (ME) and CFS specialists have developed several adult case definitions that require essential symptoms of ME and CFS: the Canadian Consensus Criteria [CCC; (3)], ME (4), ME-International Consensus Criteria [ME-ICC; (5)], and Systemic Exertion Intolerance Disease [SEID; (6)]. These case definitions are a set of rules that allows investigators and clinicians to determine who has and who does not have an illness. In other words, the goals involve sensitivity (selecting those with the illness) and specificity (not selecting those without the illness).

Criterion variance represents the largest source of diagnostic unreliability for case definitions, and it involves specifying symptoms to classify patients' symptoms into diagnostic categories (7). Criterion variance can occur when there are multiple case definitions without a consensus on which symptoms need to be manifested to arrive at a diagnosis. In addition, case definition unreliability occurs when there is no consensus on scoring rules that specify how to determine whether a particular symptom is severe enough to qualify as satisfying criteria for the case definition, or when symptoms are not assessed by standardized instruments (8). These issues can result in investigators selecting samples of patients who are different on fundamental aspects of this illness. The consequences of these types of unreliability include difficulties replicating findings at different laboratories, estimating prevalence rates, identifying biomarkers, and determining effective treatments (9).

ME and CFS case definitions (1, 3–6, 10) have some overlapping and some different diagnostic criteria. In spite of the fact that there are currently alternative case definitions, it is still important to develop standardized ways to measure the symptoms just as this has occurred with other illnesses (11). The National Institutes of Health/Centers for Disease Control and Prevention (NIH/CDC) Common Data Elements (CDE) working group has recently recommended a set of instruments to be used by researchers, and for baseline symptoms the working group recommended using either the DePaul Symptom Questionnaire [DSQ; (12)] or a combined instrument using both the CDC's Symptom Inventory [SI; (13)] as well as items from the DSQ (even though the SI and DSQ differ on a number of dimensions, including the time period in which symptoms are measured and anchor points for the assessment of symptoms). Because of the recommendation for the use of the DSQ, this article reviews the genesis and psychometric properties of the different versions of the DSQ.

## Early efforts to assess symptoms

The DePaul research team's first attempt to measure CFS symptoms based on the Fukuda et al. (1) case definition involved the development of the CFS Screening Questionnaire. The instrument assessed 22 symptoms and was administered to four groups including those with CFS, lupus, multiple sclerosis (MS), and healthy controls (14). While the screening scale had excellent test-retest and interrater reliability, and patients with CFS could be differentiated from healthy controls, those with CFS could not be differentiated from the other illnesses. Subsequently, Hawk et al. (15) developed the CFS Questionnaire, which assessed whether each of a patient's symptoms had been present for 6 months or longer, how often the symptoms were experienced (never, seldom, often/usually, always), and the intensity of each symptom on a 100 point scale (0 = no problem and 100 = the worst problem possible). We decided to measure both symptom frequency and severity, as a severe symptom that occurs infrequently, or a very mild symptom that occurs frequently might not negatively affect patients. The items had adequate reliability, and Hawk et al. (16) later found that just six variables (i.e., percentage of time fatigue reported, postexertional malaise severity, unrefreshing sleep severity, confusion–disorientation severity, shortness of breath severity, and self-reproach) could differentiate with 100% accuracy patients with CFS from those with major depressive disorder (MDD).

In the next DePaul investigation, Jason et al. (17) administered a 22-item ME/CFS Fatigue Types Questionnaire to patients with ME and CFS, and controls. Factor analyses revealed a five-factor structure for participants with ME and CFS (with one factor being PEM, whose items were later used in the original DSQ), but the controls evidenced only a one-factor solution. This questionnaire focused on different aspects of fatigue, and our next effort attempted to assemble a more comprehensive questionnaire of symptoms.

## DSQ-1

The original version of the DSQ (termed “DSQ-1”) is a self-report measure of ME and CFS symptoms, demographic characteristics, and medical, occupational, and social history (12). The DSQ-1 includes 99 items, 54 of which assess the frequency and severity of ME and CFS symptoms required by several case definitions (See Data Sheet 1). A particular focus is placed on symptoms that fall within domains specified in the CCC (3), including fatigue, PEM, neurocognitive, sleep, pain, autonomic, neuroendocrine, and immune. Participants rate each symptom's frequency over the past 6 months on a 5-point Likert-type scale (e.g., 0 = none of the time, 2 = about half the time, 4 = all of the time). Likewise, participants rate each symptom's severity over the past 6 months on a 5-point Likert-type scale (e.g., 0 = symptom not present, 2 = moderate, 4 = very severe). While frequency and severity scores are considered separately in order to determine whether participants fulfill case definitions (see Data Sheet 1), researchers can also examine each symptom's intensity by multiplying frequency and severity scores by 25 to create 100-point scales (for ease of interpretation), then averaging each symptom's frequency and severity score to create a symptom composite score.

Considerable developmental work and testing have occurred over time with this instrument. For example, Jason et al. (8) found that that a symptom of moderate or greater severity occurring at least half of the time accurately distinguishes patients from controls. A study by Evans and Jason (18) suggested that the DSQ-1's 6-month timeframe (compared to 1-week or 1-month timeframes) led to the most reliable reports of ME and CFS symptoms. Jason et al. (19) found that the DSQ exhibited good to excellent test–retest reliability, with Pearson's or kappa correlation coefficients that were 0.70 or higher for the majority of items. An early factor analysis of the DSQ-1 symptoms (*n* = 189) resulted in a three-factor solution (which included one named PEM), and these factors evidenced good internal consistency (20). A later factor analysis with a larger sample (*n* = 969) found four factors: PEM, cognitive dysfunction, sleep difficulties, and a factor consisting of neuroendocrine, autonomic, and immune symptoms (21). Using the DSQ-1, Huber et al. (22) were able to extract six potential illness subtypes after performing a latent class analysis of symptoms that loaded onto the combined factor, including those who were likely to endorse all non-core symptoms; none of the non-core symptoms; primarily gastrointestinal symptoms; primarily circulatory symptoms; gastrointestinal and circulatory symptoms; and finally those with circulatory symptoms and orthostatic intolerance.

Other research has also confirmed different psychometric properties of the DSQ-1.When Murdock et al. (23) evaluated the performance of three self-report symptom measures (the DSQ-1, Multidimensional Fatigue Inventory, and RAND SF-36) in a sample of ME and CFS patients and controls, Cronbach's alpha statistics of the 40 DSQ-1 items that loaded onto four previously-identified factors (8) were indicative of excellent internal consistency reliability (α = 0.89–0.96). This study also found that the DSQ-1 PEM items were able to differentiate between patients and controls. Furthermore, the DSQ-1 did not have problems of ceiling effects that occurred with two other patient-reported symptom measures. In another study, Jason et al. (24) found that the five PEM items from the DSQ-1 captured the widest group of patients (97%), which was higher than any other item or series of items from different scales designed to measure PEM. Strand et al. (25) compared the agreement between a physician's diagnosis [using the Canadian ME/CFS criteria; (3)] and the DSQ-1's, and found a sensitivity of 98% (*n* = 55/56); while this study initially reported a specificity of 38% (*n* = 3/8), a correction was made after subsequent analyses revealed that the five DSQ-1 “false positive” participants had documented exclusionary conditions in their DSQ-1 responses and therefore should have been classified as true negatives. In addition, our group has recently developed a subscale to measure PEM (called the DSQ-PEM scale) that includes 10 items from the DSQ-1, and findings indicate it has good sensitivity (82%) and specificity (83%) (26).

The DSQ-1 has been used for a variety of purposes, including documenting specific ME and CFS vision-related abnormalities (27). In addition, using QEEG recordings, Zinn et al. (28) estimated cortical sources and perform a functional connectivity analysis on 84 Brodmann areas representing the entire cortex. Neurocognitive impairment, as measured by the DSQ-1's cognitive composite score, was negatively associated with small-worldness index for the delta band under observation. Finally, Kemp et al. (29) found seven DSQ-1 self-reported symptoms of autonomic dysfunction [seven autonomic symptoms: bladder problems, irritable bowel problems, nausea, feeling unsteady on feet (like you might fall), shortness of breath or trouble catching your breath, dizziness or fainting, and irregular heartbeats] were found to have a significant association with low frequency heart rate variability, a measure of increased sympathetic activity.

The DSQ-1 has been translated in multiple languages, including Norwegian, Spanish, Japanese, and Persian, and used in countries around the world including Canada, England, Iran, Norway, Spain, Mexico, France, and Japan. It has been employed in data collection efforts with the Solve ME/CFS Initiative's Biobank, the CDC multi-site study, and the Chronic Fatigue Initiative.

Consistent with its primary purpose, the DSQ-1 has been successfully utilized to operationalize various ME and CFS case definitions in order to compare the symptom profiles and functional status associated with different criteria (30–33). Scoring rules enable investigators to determine which of a variety of case definitions are met (see Data Sheet 1 for the syntax of the scoring rules as well as the questionnaire). The DSQ-1 is freely available at REDCap's (34) shared library: https://redcap.is.depaul.edu/surveys/?s=H443P9TPFX. Participants are able to save their responses and return to the questionnaire as many times as needed, as severely ill individuals many not be able to complete the full questionnaire at once. This feature is available for all of the DSQ instruments that are described in this article.

## DSQ-2

After several years of using the DSQ-1, feedback from patients and researchers as well as new developments in the field prompted our group to revise the DSQ-1. The revised questionnaire is called the “DSQ-2” (see Data Sheet 2), and we added several symptoms described in the ME-ICC case definition and primer for medical practitioners (5, 35) (See Table 1). Given that the DSQ-1's development coincided with the publication of the ME-ICC (5), we were only able to use approximations for several symptoms included in this criteria. As an example, rather than ratings of frequency and severity, we only asked whether patients had experienced intolerance to extremes of temperature or viral infections with prolonged recovery periods. The DSQ-2 now collects frequency and severity data on these two ME-ICC items. We also added other items due to findings related to orthostatic intolerance and mold sensitivity, and the DSQ-2 included new PEM items based on Ramsay's (36) writings. Furthermore, two items were added based upon feedback from patients who had completed the DSQ-1. Additionally, past participants reported difficulty answering questions related to exercise and activity that referenced the past 6 months, as they had purposefully limited activity in concordance with the Energy Envelope Theory (37). To address this limitation, we added an item to address this issue. In addition, we realized that two of our items on the DSQ-1 were double-barreled, meaning they measured more than one domain. With the DSQ-2, both of these items were split into two separate symptoms. Finally, we learned that the issue of alcohol intolerance was not well-phrased in the DSQ-1, as many patients did not have this symptom over the past 6 months due to not drinking during this period of time. We thus made this a hypothetical question asking what would occur if the respondent were to drink alcohol.

**Table 1:** New Items added to the DSQ-2.

Items from the ME-ICC case definition and primer
Feeling disoriented
Slowed speech
Difficulty reading (dyslexia) after mild physical or mental activity
Aching of the eyes or behind the eyes
Sensitivity to pain
Pressure on parts of your body causes pain in other parts of your body
Daytime drowsiness
Sensitivity to vibration
Poor coordination
Sinus infections
Urinary urgency
Waking up at night because you need to urinate
Inability to tolerate an upright position
Fluctuations in temperature throughout the day
Items revised that better approximated the ME-ICC case definition
Intolerance to extremes of temperature
Viral infections with prolonged recovery periods
Items added due to findings related to orthostatic intolerance and mold sensitivity
Heart beats quickly after standing
Blurred or tunnel vision after standing
Graying or blacking out after standing,
Sensitivity to mold
Items based on Ramsay (36)
Muscle fatigue after mild physical activity
Worsening of symptoms after mild physical activity
Worsening of symptoms after mild mental activity
Items added based upon patient feedback
Since the onset of your fatigue/energy related illness
Have you stopped getting sick with colds or flus
Item added in concordance with the Energy Envelope Theory
If you were to engage in exercise or vigorous activity, would you
feel physically drained or sick?
Revised double-barreled questions
Unable to focus vision
Unablle to focus attention
Losing weight without trying
Gaining weight without trying
Issue of alcohol intolerance
What would occur if you were to drink alcohol

Using this expanded list of 78 symptoms from the DSQ-2, our team was able to extract an eight-factor structure (i.e., PEM, cognitive impairment, fever and flu, pain, sleep disruption, orthostatic intolerance, genitourinary problems, and temperature intolerance) that better tapped domains of a number of case definitions (38). In addition, using machine learning with the DSQ-2, we were able to differentiate those with ME and CFS from those with MS utilizing five symptoms, including one of our PEM items (“Next-day soreness after non-strenuous activities”) (39). Current work with the DSQ-2 has also found that patients with ME have more severe symptoms than those with MS (40) and post-polio syndrome (41).

As the DSQ-2 includes almost all of the items found in the DSQ-1, it can also be used to operationalize various ME and CFS case definitions. The DSQ-2 is freely available at REDCap's (34) shared library: https://redcap.is.depaul.edu/surveys/?s=4NJ9CKW7JD.

## DSQ-SF (short form)

In response to the expressed need of researchers and clinicians for a shorter symptom screen, our team has developed a short form of the DSQ (termed “DSQ-SF”) (see Data Sheet 3). In validating our DSQ-SF, we used two distinct samples: a multisite sample [comprised of individuals with ME and CFS (*n* = 928) and controls (*n* = 46)] and a chronic illness sample [comprised of individuals with ME and CFS (*n* = 294), and a control group of individuals with MS (*n* = 111)]. We aimed to select a small number of symptoms from each of the domains identified in the CCC [i.e., fatigue, PEM, neurocognitive, sleep, pain, autonomic, neuroendocrine, and immune; (3)], as the DSQ-1 was originally developed to measure these criteria.

Based upon the prevalence rate of symptoms and outcomes from decision trees, the following 14 items were selected for inclusion in the DSQ-SF: fatigue (fatigue domain), next-day soreness after non-strenuous activities (PEM domain), minimum exercise makes you physically tired (PEM domain), unrefreshing sleep (sleep domain), muscle pain (pain domain), bloating (pain domain), problems remembering things (neurocognitive domain), difficulty paying attention for a long period of time (neurocognitive domain), irritable bowel problems (autonomic domain), feeling unsteady on your feet, like you might fall (autonomic domain), cold limbs (neuroendocrine domain), feeling hot or cold for no reason (neuroendocrine domain), flu-like symptoms (immune domain), and some smells, foods, medications, or chemicals make you feel sick (immune domain). Sunnquist et al. (42) found, for example, in the multisite sample that relatively similar numbers of patients were identified by the DSQ-1 and the DSQ-SF (69.7% met the CCC case definition as measured by the DSQ-1, and 65.8% met the CCC as measured by the DSQ-SF algorithm).

Our findings suggest that the DSQ-SF may serve as an effective, brief symptom screen for use in time-limited research studies and clinical practice. The DSQ-SF is freely available at REDCap's (34) shared library: https://redcap.is.depaul.edu/surveys/?s=HCT7J8EWPC

## DSQ-Ped (pediatric)

Prior to the development of the DSQ-1, our research group had been using a pediatric symptom inventory (43) based on the CCC case definition (3). This symptom inventory was used to assess symptoms found in the Pediatric ME/CFS case definition developed by Jason et al. (43), which had been endorsed by the International Association of Chronic Fatigue Syndrome. We called this instrument the DePaul Pediatric Health Questionnaire, but we will now refer to it as the “DSQ-Ped.” This instrument consists of a parent form (Data Sheet 4) and a child form (Data Sheet 5). Researchers are encouraged to collect data from both children and their parents (i.e., use both forms) to obtain a thorough understanding of the child's illness. Children under the age of 12, or those with reading or comprehension difficulties, complete this questionnaire with the assistance of a parent or guardian. The symptom categories that were assessed in order to meet diagnostic criteria included fatigue, PEM, unrefreshing sleep or disturbance of sleep quantity, pain (myofascial, joint, abdominal, and/or head pain), two or more neurocognitive manifestations, and at least one symptom from two of the following categories: autonomic, neuroendocrine, or immune manifestations. There are a list of symptoms within these categories, and as with the other DSQ instruments, if the respondent indicates that a symptom meets the required frequency and severity rating, then it is counted as fulfilling that domain criterion. Rather than inquiring about symptoms within the past 6 months [as seen in the adult (1) case definition], we used a 3-month time frame. This decision was supported by the work of Fowler et al. (44), as no significant differences emerged between 8 and 17 years old with 3 vs. 6 months of chronic fatigue.

Jason et al. (45) used the DSQ-Ped in a study that compared 33 physician-referred young people with ME and CFS to 21 controls. Findings indicated that the Fukuda et al. (1) criteria in comparison to the Pediatric ME/CFS criteria were less accurate (43) in identifying cases of the illness (24% of patients would be misdiagnosed using the Fukuda criteria vs. only 3% with the Pediatric ME/CFS criteria). Jason et al. (46) also found that the DSQ-Ped was effective in distinguishing between those with severe vs. moderate pediatric ME and CFS. The severe vs. moderate categories were defined by how many symptoms the pediatric samples met, with more symptoms required for the severe category.

We are currently using an updated version of the DSQ-Ped in a community-based epidemiologic study of pediatric ME and CFS (47). This updated version, which is completed by both children and their parents/guardians in the present study, has some small differences from the original instrument, including the elimination of items that may be difficult for children to understand (e.g., next day soreness, muscle twitches, or bloating) as well as the inclusion of child-friendly phasing (e.g., using “no appetite,” “some smells, foods, or chemicals make your child feel sick,” and “upset stomach” in lieu of “nausea”). While psychometric studies of this updated questionnaire are ongoing, the symptoms assessed in this questionnaire were explicitly derived from pediatric case definitions, and the structure of the instrument mirrors that of other well-validated DSQ instruments. Furthermore, to our knowledge, the DSQ-Ped is the only pediatric-specific instrument that assesses all ME and CFS symptoms identified in case definitions. In one recent study, Schultz and Jason (48) found the orthostatic domain of the DSQ-Ped (dizziness, chest pain, shortness of breath, feeling unsteady when standing, and irregular heartbeat) significantly correlated (*r* = 0.58) with the Autonomic Symptom Checklist, which is a valid questionnaire for assessing various autonomic symptoms.

The DSQ-Ped is freely available at REDCap's (34) shared library:

DSQ-Ped (Parent Report Form): https://redcap.is.depaul.edu/surveys/?s=3FPRX49778

DSQ-Ped (Child Report Form): https://redcap.is.depaul.edu/surveys/?s=7N399W47JF

## DSQ-PSQ (pediatric screener)

We developed a pediatric screening questionnaire (termed “DSQ-PSQ,” see Data Sheet 6) for use in large-scale epidemiological studies to screen potential participants for symptoms of ME and CFS, as full medical evaluations of all participants would not be feasible. Through this questionnaire, parents or guardians are asked to report upon the health status of each of their children. There are three parts of this questionnaire; the first part focuses on whether any children in the household are experiencing prolonged fatigue or exhaustion; the second part has questions pertaining to whether any of the children are experiencing cognitive difficulties or a disruption in their school activities, as some children may be more likely to report school or cognitive challenges to their parents instead of describing the fatigue (a more complex construct to verbalize) that is causing these challenges (49). The third part of the questionnaire evaluates the (1) presence, (2) frequency, and (3) severity of 13 additional ME and CFS-related symptoms, including: frequent headaches, sore throat, joint pain, muscle pain, abdominal pain, lymph node pain, rashes, fever/chills/shivers, eye pain/light sensitivity, problems sleeping, impaired memory or concentration, feeling worse, sick, or being exhausted after exercise, and dizziness.

The DSQ-PSQ “screen positive” criteria are purposefully broad in order to avoid overlooking children with non-traditional presentations, as children who screen positive should subsequently participate in thorough medical and psychiatric exams prior to diagnosis. To screen positive, a parent must endorse that their child reports either fatigue (of at least moderate severity and present at least half of the time) or one of the school or cognitive difficulties listed in the second part of the questionnaire (at any frequency or severity level). Finally, consistent with guidelines from the Fukuda et al. (1) criteria [one of the least restrictive research criteria (30)], screen positive youth must experience at least four symptoms from the third part of the questionnaire (at any frequency or severity). Preliminary psychometric analyses show that parent ratings of their child's symptoms according to these 18 items among screen-positive children and controls, internal reliability is good (α = 0.83).

The DSQ-PSQ is also freely available at REDCap's (34) shared library: https://redcap.is.depaul.edu/surveys/?s=MFF8TXRPC8.

## Discussion

In 1994, our team began the initial development of a ME and CFS symptom scale (14). After multiple rounds of testing and refinement, we believed that we have arrived at an instrument, the DSQ-1, that is capable of effectively capturing many of the critical symptoms of ME and CFS. The evidence reviewed in this article suggests that the DSQ-1 has very good psychometric properties including test-retest reliability, sensitivity/specificity, construct, predictive, and discriminant validity. Over the past decade, ongoing efforts have broadened the instrument to include new symptoms (DSQ-2), a briefer version (DSQ-SF), a pediatric version (DSQ-Ped), and a pediatric screener (DSQ-PSQ). Developing questionnaires to ensure that key information is elicited from an interview is one of the critical tasks in operationalizing any case definition.

There are other instruments with excellent psychometric properties that have been developed to measure symptoms such as fatigue and pain (11). However, these instruments have not captured some of the core symptoms of patients with ME and CFS, such as PEM. For example, individuals with other chronic illnesses do experience some version of PEM, but their exertion-induced symptoms are primarily within the fatigue domain, whereas those with ME and CFS have post-exertion symptoms that involve multiple domains, including immune functioning such as flu-like symptoms or swollen lymph nodes (24). In addition, the onset (sometimes delayed) and duration (frequently over 24 h) of their symptoms can vary, which is also not typical of other chronic illnesses. Finally, sometimes symptoms can be reduced significantly by reducing dramatically the amount of activity engaged in. But the individuals would still experience that symptom if they exerted themselves by exceeding their energy boundaries (37). Certainly, the unique characteristics of these atypical symptoms need to be considered when assessing patients with ME and CFS.

While reliability of a case definition is enhanced with the development of questionnaires to standardize the collection of symptom data, it is also essential that a consensus be reached within the scientific community on the symptoms that must be present to satisfy a particular case definition. It is instructive to follow developments in another research area regarding issues involving the reliability of criteria for case definitions. In the 1950s and 1960s, the American Psychiatric Diagnostic and Statistical Manual (DSM)I and -II were comprised of unreliable clinical descriptions of psychiatric illnesses (7). Low interrater reliability in determining a psychiatric diagnosis was due to the inability of two interviewers to agree on the symptoms needed to be present before making a diagnosis. Low interrater reliability was due to criterion variance, deciding what symptoms or criteria were to be used to classify patients' into diagnostic categories.

In 1972, the psychiatric diagnostic Feighner criteria were developed for 16 diagnostic categories of the DSM II. This effort to be explicit about what symptoms were included within each of the 16 categories led to improvements in clinician to clinician diagnostic reliability (50). But it was not enough to have explicit, objective criteria because clinicians also needed to ensure that the diagnostic information could be elicited from an interview. Next, structured interview schedules were developed such as the Structured Clinical Interview for DSM-IV (51), and now diagnostic criteria are elicited by standardized the questions. In other words, these questionnaires reduce differences in the way clinical information is elicited. There are several lessons learned from the DSM; it is essential to develop explicit, objective criteria for a case definition, and standardized interviews can significantly improve the reliability of clinical diagnosis.

In addition to symptoms used in case definitions being clearly identified and assessed through standardized procedures, there is also a need to develop rules regarding whether a symptom is severe enough to count as a symptom for a particular case definition. As an example, the DSQ defines symptom presence as symptoms of at least moderate severity that occur at least half of the time, and there is empirical support for this cut-off. Jason et al. (32) employed a data analytic system whereby the threshold was dynamically adjusted for each DSQ-1 symptom based on observed frequency and severity scores. The results were similar to the cut-off involving at least moderate severity and occurring at least half the time, thus confirming the usefulness of this simpler-to-use criterion. Yet other cut-offs have been recommended, such as Baraniuk et al. (52), who considered complaints of *mild* or *more severe* sufficient for CFS attribution. In addition, even case definitions have at times been unclear about these cut-off points. For example, the ME-ICC case definition initially published (5) indicated a severity level of “*mild*” was equated to a 50% reduction in activity levels but later (35) a “*moderate*” severity level was equated to a 50% reduction. The above suggests there is still not a consensus on whether to use mild vs. moderate severity as cut-off thresholds, and consequently, this will influence the number of individuals identified as having ME or CFS symptoms.

As another example of this variation, Reeves et al. (53) Symptom Inventory requires symptoms to occur within the past month rather than the past 6 months (as required by the DSQ). The 1-month requirement may inflate the number individuals classified as having ME and CFS and capture, for example, those who experienced severe sore throats in the past month due to influenza. It is not just the rules governing cut-off thresholds and length of time that varies among investigators, but also how symptoms are summed to determine whether a person meets ME or CFS criteria. For example, the case definition proposed by Reeves et al. (53) would be met if an individual rated only two symptoms as occurring all the time, and one was of moderate and the other of severe severity. Therefore, the overall level of symptoms might be low for some patients with this summary method.

The reliability of a case definition also depends upon the operationalization of other frequently included criteria. For example, this includes a “substantial reduction in functioning” (54–56), “lifelong fatigue” (57), “fatigue not substantially alleviated by rest,” and “fatigue that is the result of excessive exertion” (56). Attempts to concretely define these criteria have been met with considerable controversy [e.g., (58)]. For example, Reeves et al. (53) operationalized the way a patient's substantial reduction in functioning was measured using what was called the “empiric criteria.” These researchers selected an instrument [i.e., the 36-Item Short Form Health Survey; SF-36, (59)], and if a patient met criteria for one of several specified subscales within the SF-36, the patient would meet the substantial reduction criteria for having CFS. However, one of these domains was “role emotional” functioning, and every person with a diagnosis of MDD would meet the criteria for “role emotional” functioning (60). This example demonstrates the necessity of specifying not only the instruments to be used, but also which of the instrument's subscales are appropriate and what the cut-off points are for meeting the threshold for disability. If mistakes occur on these critical choice points, it is possible that individuals with other illnesses will be misdiagnosed. To illustrate this point, using the Reeves et al. (53) “empiric criteria”, with its decision to use “role emotional” functioning as a measure of substantial reduction, over one-third of individuals with MDD might have been inappropriately classified as having CFS (60). These types of decisions on how to assess substantial reductions in functioning as well as other decisions such as counting a symptom as needing to occur for only 1 rather than 6 months could be responsible for the estimated 10-fold increase in CDC prevalence estimates of CFS that occurred from 2003 to 2007 (9).

As mentioned in the introduction, the CDC/NIH CDE tasks were to recommend instruments that could be used by investigators to study ME and CFS, but they did not specify what subscales to use or scoring rules regarding thresholds that needed to be met. Criterion variance can occur when specifications of subscales, scoring rules or case definition are not specified. Without such specification, the same symptoms may not be described in different case definitions (1, 3, 5, 6, 61). As stated by Janson et al. (9), in addition to recommending measures, reducing criterion variance will only occur when there is a consensus on what subscales, scoring rules, and research case definition is to be employed in different settings (7).

A report from the IOM (6) recommended a “continuing surveillance of the evidence and revisiting of the criteria in no more than 5 years” (p. 188). Two years later, an NIH request for funding of ME/CFS centers recommended “that the investigators utilize the Canadian Consensus Criteria for ME/CFS as proposed by Carruther[s] and colleagues in 2003 and revised by Jason and colleagues in 2010, and the recent case definition from the Institute of Medicine Report on ME/CFS” (62). This NIH funding request suggests that the federal government has preferences for grant applications that use these two sets of criteria. Yet these case definitions were developed as clinical rather than research case definitions. Some prefer a broader perspective and others a more narrow one in the diagnosis and case definition, and both positions have some merit, and we might eventually call one more clinical criteria and the more research oriented. As an example, Jason et al. (63) suggested the following classification system, those with just chronic fatigue of 6 or more months would be the broadest category (similar to the Oxford criteria), those who meet a ME/CFS clinical criteria would be represented by the IOM (6) criteria (with few exclusionary illnesses), and a purer ME criteria could be based either on the Canadian Consensus Criteria (3) or work of Ramsay (36). Sophisticated and methodologically sound research methods could also be used to select and operationalize criteria for a research case definition (22, 31, 32, 64).

Our article highlights the development of DSQ in various forms. This type of interview schedule ensures that necessary symptom information is elicited reliably from an interview. This instrument is one of a variety of measures being recommended by the CDC NIH/CDE to assess ME and CFS domains, but there is now a need to also recommend a research case definition, as well as reach a consensus on other critical case definition criteria, such determining which subscales to use, what thresholds determine symptoms counting as a problem, and how to operationalize substantial reduction in functioning, lifelong fatigue, fatigue not substantially alleviated by rest, and fatigue that is the result of excessive exertion. Using large data sets and sophisticated research methods, we can work toward coming to a consensus on these issues.

An international, transparent, and inclusive effort, involving scientists, patient organizations, and government groups, could be assembled to resolve these fundamental reliability and diagnostic issues.

## Author contributions

LJ wrote the first draft of this article and was engaged in the research efforts on various versions of the DSQ over the past few decades. He also helped with the development of the Supplemental Materials. MS helped write the article and was primarily responsible for writing the Supplemental Materials that involved scoring rules and syntax for the case definitions.

### Conflict of interest statement

The authors declare that the research was conducted in the absence of any commercial or financial relationships that could be construed as a potential conflict of interest.
