# Supplementary material for: The Development of the DePaul Symptom Questionnaire: Original, Expanded, Brief, and Pediatric Versions
Source: Front Pediatr. 2018 Nov 6;6:330. doi: 10.3389/fped.2018.00330 (PMC6232226; doi:10.3389/fped.2018.00330)
Supplement: Supplementary file 1 [file Data_Sheet_1.docx]

**Data Sheet 1.**

**DePaul Symptom Questionnaire (DSQ-1)**

This document contains the following material:

1. Case definition scoring rules and associated symptoms for the following criteria:
   1. Fukuda et al. (1994)
   2. Canadian ME/CFS (Carruthers et al., 2003)
   3. ME-ICC (Carruthers et al., 2011)
   4. Institute of Medicine (IOM, 2015)
2. Syntax for the following case definitions:
   1. Fukuda et al. (1994)
   2. Canadian ME/CFS (Carruthers et al., 2003)
   3. ME-ICC (Carruthers et al., 2011)
   4. Institute of Medicine (IOM, 2015)
3. Hard copy of the DSQ-1 and the SF-36

The DePaul Symptom Questionnaire (DSQ-1) can be downloaded from the REDCap shared library. You can view the instrument here:

<https://redcap.is.depaul.edu/surveys/?s=H443P9TPFX>

**DSQ-1**

**Case Definition Criteria:**

**Fukuda (Fukuda et al., 1994)**

- Substantial Reduction in Functioning (must meet 2 of the following 3 SF-36 score cutoffs):
  - Role Physical <= 50
  - Social Functioning <= 62.5
  - Vitality <= 35
- 6+ months of fatigue (Question 69)
  - Fatigue *not* lifelong (*Exclude* if: Question 67 = “Yes” *and* Question 69 = “*Had problem since childhood/adolescence*”… *and* Question 77 = “*Over 3 or more years*”)
  - Fatigue *not* the result of exertion: (*Exclude* if: Sum of Question 89a and 89d >= 60)
- At least 1 symptom (frequency and severity ratings >=1) from at least 4 symptom domains:
  - Memory / Concentration (Questions 36-39; 43-44)
  - Unrefreshing Sleep (Question 19)
  - Joint Pain (Question 26)
  - Tender / Sore Lymph Nodes (Question 63)
  - Muscle Aches (Question 25)
  - Post-Exertional Malaise (Questions 14-18)
  - Headaches (Question 31; must also be of a new place/type, Question 68)
  - Sore Throat (Question 62)

**Canadian Consensus Criteria (CCC Case Definition, Carruthers et al., 2003)**

- Substantial Reduction in Functioning (must meet 2 of the following 3 SF-36 score cutoffs):
  - Role Physical <= 50
  - Social Functioning <= 62.5
  - Vitality <= 35
- Fatigue:
  - 6+ months of fatigue (Question 69)
  - Fatigue frequency and severity >= 2 (Question 13)
  - Fatigue *not* lifelong (*Exclude* if: Question 67 = “Yes” *and* Question 69 = “*Had problem since childhood/adolescence*”… *and* Question 77 = “*Over 3 or more years*”)
  - Fatigue *not* the result of exertion: (*Exclude* if: Sum of Question 89a and 89d >= 60)
- Post-Exertional Malaise (At least 1 symptom (frequency and severity ratings >=2) from questions 14-18)
- Sleep Problems (At least 1 symptom (frequency and severity ratings >=2) from questions 19-24)
- Pain (At least 1 symptom (frequency and severity ratings >=2) from questions 25-31)
- Neurological / Cognitive Problems (At least 2 symptoms (frequency and severity ratings >=2) from questions 32-44)
- At least 1 symptom (frequency and severity ratings >=2) from 2 of the 3 following areas:
  - Autonomic (Questions 45-51)
  - Neuroendocrine (Questions 52-61)
  - Immune (Questions 62-66)

**Myalgic Encephalomyelitis International Consensus Criteria (ME-ICC; Carruthers et al., 2011)**

- 50% reduction in activity level (“Yes” to Question 97)
- Post Exertional Malaise (At least one symptom (frequency and severity ratings >=2) from question 14-18)
- At least 1 symptom (frequency and severity ratings >=2) from 3 of the following 4 symptom domains:
  - Neurocognitive: Questions 36-44
  - Pain: Questions 25-28; 31
  - Sleep Disturbance: Questions 19-24
  - Neurosensory, Perceptual, and Motor Disturbance: Questions 32-35; 48
- At least 1 symptom (frequency and severity ratings >=2; except for Question 98, which requires a response of “Yes”) from 3 of the following 5 symptom domains:
  - Flu-like: Questions 62-65
  - Gastrointestinal: Questions 29-30; 46-47
  - Genitourinary: Question 45
  - Sensitivities: Questions 61, 66
  - Susceptibility to Viral Infections: Question 98
- At least 1 symptom (frequency and severity ratings >=2; except for Question 99, which requires a response of “Yes”) from 1 of the following 4 symptom domains:
  - Cardiovascular: Questions 50-51
  - Respiratory: Question 49
  - Loss of thermostatic ability: Questions 54-60
  - Temperature intolerance: Question 99

**IOM Clinical Case Definition** (IOM, 2015):

- Substantial Reduction in functioning (must meet 2 of the following 3 SF-36 score cutoffs):
  - Role Physical <= 50
  - Social Functioning <= 62.5
  - Vitality <= 35
- 6+ months of fatigue (Question 69)
  - Fatigue *not* lifelong (*Exclude* if: Question 67 = “Yes” *and* Question 69 = “*Had problem since childhood/adolescence*”… *and* Question 77 = “*Over 3 or more years*”)
  - Fatigue *not* the result of exertion: (*Exclude* if: Sum of Question 89a and 89d >= 60)
- Post-Exertional Malaise (At least 1 symptom (frequency and severity ratings >= 2) from questions 14-18)
- Unrefreshing Sleep (At least 1 symptom (frequency and severity ratings >= 2) from questions 19-22; 24)
- At least 1 symptom (frequency and severity ratings >= 2) from 1 of the following 2 symptom domains:
  - Cognitive Impairment (Questions 36-40; 43-44)
  - Orthostatic Intolerance (Questions 48-51)

**DSQ-1**

**SPSS Case Definition Syntax:**

**Scoring Note: To determine which variables are associated with which items, note that variable names utilize the items numbers present in the questionnaire*.*

*****************************************************************************.

*DSQ-1*.

*Fukuda et al. (1994) Criteria*.

*****************************************************************************.

***Substantial Reduction in Functioning Criteria, SF-36***.

***To score the SF-36, use scoring rules found here:

***https://www.rand.org/health/surveys_tools/mos/36-item-short-form/scoring.html***.

***Variable names are equivalent to SF-36 Subscale Names***.

COMPUTE Fukuda_RP = 0.

EXECUTE.

IF (RolePhysical <= 50) Fukuda_RP = 1.

EXECUTE.

COMPUTE Fukuda_SF = 0.

EXECUTE.

IF (SocialFunctioning <= 62.5) Fukuda_SF = 1.

EXECUTE.

COMPUTE Fukuda_V = 0.

EXECUTE.

IF (Vitality <= 35) Fukuda_V = 1.

EXECUTE.

COMPUTE Fukuda_SR=0.

EXECUTE.

IF (SUM(Fukuda_RP, Fukuda_SF, Fukuda_V) >= 2) Fukuda_SR = 1.

EXECUTE.

***DSQ-1 Symptom Scoring***.

*Fatigue, Not Lifelong, Not Result of Exertion *.

COMPUTE Fukuda_Six = 0.

EXECUTE.

IF ((dsq_69 = 2) | (dsq_69 = 3) | (dsq_69 = 4) | (dsq_69 = 5)) Fukuda_Six = 1.

EXECUTE.

COMPUTE Fukuda_Life = 0.

EXECUTE.

IF ((dsq_67 = 1) & (dsq_69 = 5) & (dsq_77 = 7)) Fukuda_Life = 1.

EXECUTE.

COMPUTE Fukuda_Exert = 0.

EXECUTE.

IF((SUM(dsq_89a, dsq_89d) >= 60)) Fukuda_Exert = 1.

EXECUTE.

COMPUTE Fukuda_Fatigue = 0.

EXECUTE.

IF ((Fukuda_Six = 1) & (Fukuda_Life = 0) & (Fukuda_Exert = 0)) Fukuda_Fatigue = 1.

EXECUTE.

*Post-Exertional Malaise*.

COMPUTE Fukuda_14 = 0.

EXECUTE.

IF ((dsq_14f >= 1) & (dsq_14s >= 1)) Fukuda_14 = 1.

EXECUTE.

COMPUTE Fukuda_15 = 0.

EXECUTE.

IF ((dsq_15f >= 1) & (dsq_15s >= 1)) Fukuda_15 = 1.

EXECUTE.

COMPUTE Fukuda_16 = 0.

EXECUTE.

IF ((dsq_16f >= 1) & (dsq_16s >= 1)) Fukuda_16 = 1.

EXECUTE.

COMPUTE Fukuda_17 = 0.

EXECUTE.

IF ((dsq_17f >= 1) & (dsq_17s >= 1)) Fukuda_17 = 1.

EXECUTE.

COMPUTE Fukuda_18 = 0.

EXECUTE.

IF ((dsq_18f >= 1) & (dsq_18s >= 1)) Fukuda_18 = 1.

EXECUTE.

COMPUTE Fukuda_PEM = 0.

EXECUTE.

IF(SUM(Fukuda_14, Fukuda_15, Fukuda_16, Fukuda_17, Fukuda_18) >= 1) Fukuda_PEM = 1.

EXECUTE.

*Unrefreshing Sleep*.

COMPUTE Fukuda_Sleep = 0.

EXECUTE.

IF ((dsq_19f >= 1) & (dsq_19s >= 1)) Fukuda_Sleep = 1.

EXECUTE.

*Muscle Aches*.

COMPUTE Fukuda_Muscle = 0.

EXECUTE.

IF ((dsq_25f >= 1) & (dsq_25s >= 1)) Fukuda_Muscle = 1.

EXECUTE.

*Joint Pain*.

COMPUTE Fukuda_Joint = 0.

EXECUTE.

IF ((dsq_26f >= 1) & (dsq_26s >= 1)) Fukuda_Joint = 1.

EXECUTE.

*Headaches*.

COMPUTE Fukuda_Headache = 0.

EXECUTE.

IF ((dsq_31f >= 1) & (dsq_31s >= 1) & (dsq_68 = 1)) Fukuda_Headache = 1.

EXECUTE.

*Memory and Concentration*.

COMPUTE Fukuda_36 = 0.

EXECUTE.

IF ((dsq_36f >= 1) & (dsq_36s >= 1)) Fukuda_36 = 1.

EXECUTE.

COMPUTE Fukuda_37 = 0.

EXECUTE.

IF ((dsq_37f >= 1) & (dsq_37s >= 1)) Fukuda_37 = 1.

EXECUTE.

COMPUTE Fukuda_38 = 0.

EXECUTE.

IF ((dsq_38f >= 1) & (dsq_38s >= 1)) Fukuda_38 = 1.

EXECUTE.

COMPUTE Fukuda_39 = 0.

EXECUTE.

IF ((dsq_39f >= 1) & (dsq_39s >= 1)) Fukuda_39 = 1.

EXECUTE.

COMPUTE Fukuda_43 = 0.

EXECUTE.

IF ((dsq_43f >= 1) & (dsq_43s >= 1)) Fukuda_43 = 1.

EXECUTE.

COMPUTE Fukuda_44 = 0.

EXECUTE.

IF ((dsq_44f >= 1) & (dsq_44s >= 1)) Fukuda_44 = 1.

EXECUTE.

COMPUTE Fukuda_Memory = 0.

EXECUTE.

IF (SUM(Fukuda_36, Fukuda_37, Fukuda_38, Fukuda_39, Fukuda_43, Fukuda_44) >= 1) Fukuda_Memory = 1.

EXECUTE.

* Sore Throat*.

COMPUTE Fukuda_Throat = 0.

EXECUTE.

IF ((dsq_62f >= 1) & (dsq_62s >= 1)) Fukuda_Throat = 1.

EXECUTE.

*Lymph Nodes*.

COMPUTE Fukuda_Lymph = 0.

EXECUTE.

IF ((dsq_63f >= 1) & (dsq_63s >= 1)) Fukuda_Lymph = 1.

EXECUTE.

***Fukuda Case Definition***.

COMPUTE Fukuda = 0.

EXECUTE.

IF((Fukuda_SR = 1) & (Fukuda_Fatigue = 1) & (SUM(Fukuda_PEM, Fukuda_Sleep, Fukuda_Muscle, Fukuda_Joint, Fukuda_Headache, Fukuda_Memory, Fukuda_Throat, Fukuda_Lymph) >= 4)) Fukuda = 1.

EXECUTE.

VALUE LABELS

Fukuda

0 ‘Does Not Meet Fukuda Criteria’

1 ‘Meets Fukuda Criteria’.

EXECUTE.

*****************************************************************************.

*DSQ-1*.

*Canadian Clinical ME/CFS Criteria (CCC; Carruthers et al., 2003)*.

*****************************************************************************.

***Substantial Reduction in Functioning Criteria, SF-36***.

***To score the SF-36, use scoring rules found here:

***https://www.rand.org/health/surveys_tools/mos/36-item-short-form/scoring.html***.

***Variable names are equivalent to SF-36 Subscale Names***.

COMPUTE CCC_RP = 0.

EXECUTE.

IF (RolePhysical <= 50) CCC_RP = 1.

EXECUTE.

COMPUTE CCC_SF = 0.

EXECUTE.

IF (SocialFunctioning <= 62.5) CCC_SF = 1.

EXECUTE.

COMPUTE CCC_V = 0.

EXECUTE.

IF (Vitality <= 35) CCC_V = 1.

EXECUTE.

COMPUTE CCC_SR=0.

EXECUTE.

IF (SUM(CCC_RP, CCC_SF, CCC_V) >= 2) CCC_SR = 1.

EXECUTE.

***DSQ-1 Symptom Scoring***.

*Fatigue, Not Lifelong, Not Result of Exertion*.

COMPUTE CCC_Six = 0.

EXECUTE.

IF ((dsq_69 = 2) | (dsq_69 = 3) | (dsq_69 = 4) | (dsq_69 = 5)) CCC_Six = 1.

EXECUTE.

COMPUTE CCC_13 = 0.

EXECUTE.

IF ((dsq_13f >= 2) & (dsq_13s >= 2)) CCC_13 = 1.

EXECUTE.

COMPUTE CCC_Life = 0.

EXECUTE.

IF ((dsq_67 = 1) & (dsq_69 = 5) & (dsq_77 = 7)) CCC_Life = 1.

EXECUTE.

COMPUTE CCC_Exert = 0.

EXECUTE.

IF((SUM(dsq_89a, dsq_89d) >= 60)) CCC_Exert = 1.

EXECUTE.

COMPUTE CCC_Fatigue = 0.

EXECUTE.

IF ((CCC_Six = 1) & (CCC_13 = 1) & (CCC_Life = 0) & (CCC_Exert = 0)) CCC_Fatigue = 1.

EXECUTE.

*Post-Exertional Malaise*.

COMPUTE CCC_14 = 0.

EXECUTE.

IF ((dsq_14f >= 2) & (dsq_14s >= 2)) CCC_14 = 1.

EXECUTE.

COMPUTE CCC_15 = 0.

EXECUTE.

IF ((dsq_15f >= 2) & (dsq_15s >= 2)) CCC_15 = 1.

EXECUTE.

COMPUTE CCC_16 = 0.

EXECUTE.

IF ((dsq_16f >= 2) & (dsq_16s >= 2)) CCC_16 = 1.

EXECUTE.

COMPUTE CCC_17 = 0.

EXECUTE.

IF ((dsq_17f >= 2) & (dsq_17s >= 2)) CCC_17 = 1.

EXECUTE.

COMPUTE CCC_18 = 0.

EXECUTE.

IF ((dsq_18f >= 2) & (dsq_18s >= 2)) CCC_18 = 1.

EXECUTE.

COMPUTE CCC_PEM = 0.

EXECUTE.

IF(SUM(CCC_14, CCC_15, CCC_16, CCC_17, CCC_18) >= 1) CCC_PEM = 1.

EXECUTE.

*Sleep*.

COMPUTE CCC_19 = 0.

EXECUTE.

IF ((dsq_19f >= 2) & (dsq_19s >= 2)) CCC_19 = 1.

EXECUTE.

COMPUTE CCC_20 = 0.

EXECUTE.

IF ((dsq_20f >= 2) & (dsq_20s >= 2)) CCC_20 = 1.

EXECUTE.

COMPUTE CCC_21 = 0.

EXECUTE.

IF ((dsq_21f >= 2) & (dsq_21s >= 2)) CCC_21 = 1.

EXECUTE.

COMPUTE CCC_22 = 0.

EXECUTE.

IF ((dsq_22f >= 2) & (dsq_22s >= 2)) CCC_22 = 1.

EXECUTE.

COMPUTE CCC_23 = 0.

EXECUTE.

IF ((dsq_23f >= 2) & (dsq_23s >= 2)) CCC_23 = 1.

EXECUTE.

COMPUTE CCC_24 = 0.

EXECUTE.

IF ((dsq_24f >= 2) & (dsq_24s >= 2)) CCC_24 = 1.

EXECUTE.

COMPUTE CCC_Sleep = 0.

EXECUTE.

IF(SUM(CCC_19, CCC_20, CCC_21, CCC_22, CCC_23, CCC_24) >= 1) CCC_Sleep = 1.

EXECUTE.

*Pain*.

COMPUTE CCC_25 = 0.

EXECUTE.

IF ((dsq_25f >= 2) & (dsq_25s >= 2)) CCC_25 = 1.

EXECUTE.

COMPUTE CCC_26 = 0.

EXECUTE.

IF ((dsq_26f >= 2) & (dsq_26s >= 2)) CCC_26 = 1.

EXECUTE.

COMPUTE CCC_27 = 0.

EXECUTE.

IF ((dsq_27f >= 2) & (dsq_27s >= 2)) CCC_27 = 1.

EXECUTE.

COMPUTE CCC_28 = 0.

EXECUTE.

IF ((dsq_28f >= 2) & (dsq_28s >= 2)) CCC_28 = 1.

EXECUTE.

COMPUTE CCC_29 = 0.

EXECUTE.

IF ((dsq_29f >= 2) & (dsq_29s >= 2)) CCC_29 = 1.

EXECUTE.

COMPUTE CCC_30 = 0.

EXECUTE.

IF ((dsq_30f >= 2) & (dsq_30s >= 2)) CCC_30 = 1.

EXECUTE.

COMPUTE CCC_31 = 0.

EXECUTE.

IF ((dsq_31f >= 2) & (dsq_31s >= 2) & (dsq_68 = 1)) CCC_31 = 1.

EXECUTE.

COMPUTE CCC_Pain = 0.

EXECUTE.

IF(SUM(CCC_25, CCC_26, CCC_27, CCC_28, CCC_29, CCC_30, CCC_31) >= 1) CCC_Pain = 1.

EXECUTE.

*Neurocognitive*.

COMPUTE CCC_32 = 0.

EXECUTE.

IF ((dsq_32f >= 2) & (dsq_32s >= 2)) CCC_32 = 1.

EXECUTE.

COMPUTE CCC_33 = 0.

EXECUTE.

IF ((dsq_33f >= 2) & (dsq_33s >= 2)) CCC_33 = 1.

EXECUTE.

COMPUTE CCC_34 = 0.

EXECUTE.

IF ((dsq_34f >= 2) & (dsq_34s >= 2)) CCC_34 = 1.

EXECUTE.

COMPUTE CCC_35 = 0.

EXECUTE.

IF ((dsq_35f >= 2) & (dsq_35s >= 2)) CCC_35 = 1.

EXECUTE.

COMPUTE CCC_36 = 0.

EXECUTE.

IF ((dsq_36f >= 2) & (dsq_36s >= 2)) CCC_36 = 1.

EXECUTE.

COMPUTE CCC_37 = 0.

EXECUTE.

IF ((dsq_37f >= 2) & (dsq_37s >= 2)) CCC_37 = 1.

EXECUTE.

COMPUTE CCC_38 = 0.

EXECUTE.

IF ((dsq_38f >= 2) & (dsq_38s >= 2)) CCC_38 = 1.

EXECUTE.

COMPUTE CCC_39 = 0.

EXECUTE.

IF ((dsq_39f >= 2) & (dsq_39s >= 2)) CCC_39 = 1.

EXECUTE.

COMPUTE CCC_40 = 0.

EXECUTE.

IF ((dsq_40f >= 2) & (dsq_40s >= 2)) CCC_40 = 1.

EXECUTE.

COMPUTE CCC_41 = 0.

EXECUTE.

IF ((dsq_41f >= 2) & (dsq_41s >= 2)) CCC_41 = 1.

EXECUTE.

COMPUTE CCC_42 = 0.

EXECUTE.

IF ((dsq_42f >= 2) & (dsq_42s >= 2)) CCC_42 = 1.

EXECUTE.

COMPUTE CCC_43 = 0.

EXECUTE.

IF ((dsq_43f >= 2) & (dsq_43s >= 2)) CCC_43 = 1.

EXECUTE.

COMPUTE CCC_44 = 0.

EXECUTE.

IF ((dsq_44f >= 2) & (dsq_44s >= 2)) CCC_44 = 1.

EXECUTE.

COMPUTE CCC_Neurocog = 0.

EXECUTE.

IF(SUM(CCC_32, CCC_33, CCC_34, CCC_35, CCC_36, CCC_37, CCC_38, CCC_39, CCC_40, CCC_41, CCC_42, CCC_43, CCC_44) >= 2) CCC_Neurocog = 1.

EXECUTE.

*Autonomic*.

COMPUTE CCC_45 = 0.

EXECUTE.

IF ((dsq_45f >= 2) & (dsq_45s >= 2)) CCC_45 = 1.

EXECUTE.

COMPUTE CCC_46 = 0.

EXECUTE.

IF ((dsq_46f >= 2) & (dsq_46s >= 2)) CCC_46 = 1.

EXECUTE.

COMPUTE CCC_47 = 0.

EXECUTE.

IF ((dsq_47f >= 2) & (dsq_47s >= 2)) CCC_47 = 1.

EXECUTE.

COMPUTE CCC_48 = 0.

EXECUTE.

IF ((dsq_48f >= 2) & (dsq_48s >= 2)) CCC_48 = 1.

EXECUTE.

COMPUTE CCC_49 = 0.

EXECUTE.

IF ((dsq_49f >= 2) & (dsq_49s >= 2)) CCC_49 = 1.

EXECUTE.

COMPUTE CCC_50 = 0.

EXECUTE.

IF ((dsq_50f >= 2) & (dsq_50s >= 2)) CCC_50 = 1.

EXECUTE.

COMPUTE CCC_51 = 0.

EXECUTE.

IF ((dsq_51f >= 2) & (dsq_51s >= 2)) CCC_51 = 1.

EXECUTE.

COMPUTE CCC_Auto = 0.

EXECUTE.

IF(SUM(CCC_45, CCC_46, CCC_47, CCC_48, CCC_49, CCC_50, CCC_51) >= 1) CCC_Auto = 1.

EXECUTE.

*Neuroendocrine*.

COMPUTE CCC_52 = 0.

EXECUTE.

IF ((dsq_52f >= 2) & (dsq_52s >= 2)) CCC_52 = 1.

EXECUTE.

COMPUTE CCC_53 = 0.

EXECUTE.

IF ((dsq_53f >= 2) & (dsq_53s >= 2)) CCC_53 = 1.

EXECUTE.

COMPUTE CCC_54 = 0.

EXECUTE.

IF ((dsq_54f >= 2) & (dsq_54s >= 2)) CCC_54 = 1.

EXECUTE.

COMPUTE CCC_55 = 0.

EXECUTE.

IF ((dsq_55f >= 2) & (dsq_55s >= 2)) CCC_55 = 1.

EXECUTE.

COMPUTE CCC_56 = 0.

EXECUTE.

IF ((dsq_56f >= 2) & (dsq_56s >= 2)) CCC_56 = 1.

EXECUTE.

COMPUTE CCC_57 = 0.

EXECUTE.

IF ((dsq_57f >= 2) & (dsq_57s >= 2)) CCC_57 = 1.

EXECUTE.

COMPUTE CCC_58 = 0.

EXECUTE.

IF ((dsq_58f >= 2) & (dsq_58s >= 2)) CCC_58 = 1.

EXECUTE.

COMPUTE CCC_59 = 0.

EXECUTE.

IF ((dsq_59f >= 2) & (dsq_59s >= 2)) CCC_59 = 1.

EXECUTE.

COMPUTE CCC_60 = 0.

EXECUTE.

IF ((dsq_60f >= 2) & (dsq_60s >= 2)) CCC_60 = 1.

EXECUTE.

COMPUTE CCC_61 = 0.

EXECUTE.

IF ((dsq_61f >= 2) & (dsq_61s >= 2)) CCC_61 = 1.

EXECUTE.

COMPUTE CCC_Neuroendo = 0.

EXECUTE.

IF(SUM(CCC_52, CCC_53, CCC_54, CCC_55, CCC_56, CCC_57, CCC_58, CCC_59, CCC_60, CCC_61) >= 1) CCC_Neuroendo = 1.

EXECUTE.

*Immune*.

COMPUTE CCC_62 = 0.

EXECUTE.

IF ((dsq_62f >= 2) & (dsq_62s >= 2)) CCC_62 = 1.

EXECUTE.

COMPUTE CCC_63 = 0.

EXECUTE.

IF ((dsq_63f >= 2) & (dsq_63s >= 2)) CCC_63 = 1.

EXECUTE.

COMPUTE CCC_64 = 0.

EXECUTE.

IF ((dsq_64f >= 2) & (dsq_64s >= 2)) CCC_64 = 1.

EXECUTE.

COMPUTE CCC_65 = 0.

EXECUTE.

IF ((dsq_65f >= 2) & (dsq_65s >= 2)) CCC_65 = 1.

EXECUTE.

COMPUTE CCC_66 = 0.

EXECUTE.

IF ((dsq_66f >= 2) & (dsq_66s >= 2)) CCC_66 = 1.

EXECUTE.

COMPUTE CCC_Immune = 0.

EXECUTE.

IF(SUM(CCC_62, CCC_63, CCC_64, CCC_65, CCC_66) >= 1) CCC_Immune = 1.

EXECUTE.

***Canadian ME/CFS Case Definition***.

COMPUTE CCC_ANI2 = 0.

EXECUTE.

IF(SUM(CCC_Auto, CCC_Neuroendo, CCC_Immune) >= 2) CCC_ANI2 = 1.

EXECUTE.

COMPUTE CCC = 0.

EXECUTE.

IF(SUM(CCC_SR, CCC_Fatigue, CCC_PEM, CCC_Sleep, CCC_Pain, CCC_Neurocog, CCC_ANI2) = 7) CCC = 1.

EXECUTE.

VALUE LABELS

CCC

0 ‘Does Not Meet Canadian MECFS Criteria’

1 ‘Meets Canadian MECFS Criteria’.

EXECUTE.

*****************************************************************************.

*DSQ-1*.

*International Consensus Criteria for ME (ME-ICC; Carruthers et al., 2011)*.

*****************************************************************************.

**50 Percent Reduction in Activity**.

COMPUTE MEICC_SR = 0.

EXECUTE.

IF(dsq_97 = 1) MEICC_SR = 1.

EXECUTE.

**PENE**.

COMPUTE MEICC_14 = 0.

EXECUTE.

IF ((dsq_14f >= 2) & (dsq_14s >= 2)) MEICC_14 = 1.

EXECUTE.

COMPUTE MEICC_15 = 0.

EXECUTE.

IF ((dsq_15f >= 2) & (dsq_15s >= 2)) MEICC_15 = 1.

EXECUTE.

COMPUTE MEICC_16 = 0.

EXECUTE.

IF ((dsq_16f >= 2) & (dsq_16s >= 2)) MEICC_16 = 1.

EXECUTE.

COMPUTE MEICC_17 = 0.

EXECUTE.

IF ((dsq_17f >= 2) & (dsq_17s >= 2)) MEICC_17 = 1.

EXECUTE.

COMPUTE MEICC_18 = 0.

EXECUTE.

IF ((dsq_18f >= 2) & (dsq_18s >= 2)) MEICC_18 = 1.

EXECUTE.

COMPUTE MEICC_PENE = 0.

EXECUTE.

IF(SUM(MEICC_14, MEICC_15, MEICC_16, MEICC_17, MEICC_18) >= 1) MEICC_PENE = 1.

EXECUTE.

**Neurological Impairments**.

*Neurocognitive*.

COMPUTE MEICC_36 = 0.

EXECUTE.

IF ((dsq_36f >= 2) & (dsq_36s >= 2)) MEICC_36 = 1.

EXECUTE.

COMPUTE MEICC_37 = 0.

EXECUTE.

IF ((dsq_37f >= 2) & (dsq_37s >= 2)) MEICC_37 = 1.

EXECUTE.

COMPUTE MEICC_38 = 0.

EXECUTE.

IF ((dsq_38f >= 2) & (dsq_38s >= 2)) MEICC_38 = 1.

EXECUTE.

COMPUTE MEICC_39 = 0.

EXECUTE.

IF ((dsq_39f >= 2) & (dsq_39s >= 2)) MEICC_39 = 1.

EXECUTE.

COMPUTE MEICC_40 = 0.

EXECUTE.

IF ((dsq_40f >= 2) & (dsq_40s >= 2)) MEICC_40 = 1.

EXECUTE.

COMPUTE MEICC_41 = 0.

EXECUTE.

IF ((dsq_41f >= 2) & (dsq_41s >= 2)) MEICC_41 = 1.

EXECUTE.

COMPUTE MEICC_42 = 0.

EXECUTE.

IF ((dsq_42f >= 2) & (dsq_42s >= 2)) MEICC_42 = 1.

EXECUTE.

COMPUTE MEICC_43 = 0.

EXECUTE.

IF ((dsq_43f >= 2) & (dsq_43s >= 2)) MEICC_43 = 1.

EXECUTE.

COMPUTE MEICC_44 = 0.

EXECUTE.

IF ((dsq_44f >= 2) & (dsq_44s >= 2)) MEICC_44 = 1.

EXECUTE.

COMPUTE MEICC_Cognitive = 0.

EXECUTE.

IF(SUM(MEICC_36, MEICC_37, MEICC_38, MEICC_39, MEICC_40, MEICC_41, MEICC_42, MEICC_43, MEICC_44) >= 1) MEICC_Cognitive = 1.

EXECUTE.

*Pain*.

COMPUTE MEICC_25 = 0.

EXECUTE.

IF ((dsq_25f >= 2) & (dsq_25s >= 2)) MEICC_25 = 1.

EXECUTE.

COMPUTE MEICC_26 = 0.

EXECUTE.

IF ((dsq_26f >= 2) & (dsq_26s >= 2)) MEICC_26 = 1.

EXECUTE.

COMPUTE MEICC_27 = 0.

EXECUTE.

IF ((dsq_27f >= 2) & (dsq_27s >= 2)) MEICC_27 = 1.

EXECUTE.

COMPUTE MEICC_28 = 0.

EXECUTE.

IF ((dsq_28f >= 2) & (dsq_28s >= 2)) MEICC_28 = 1.

EXECUTE.

COMPUTE MEICC_31 = 0.

EXECUTE.

IF ((dsq_31f >= 2) & (dsq_31s >= 2)) MEICC_31 = 1.

EXECUTE.

COMPUTE MEICC_Pain = 0.

EXECUTE.

IF(SUM(MEICC_25, MEICC_26, MEICC_27, MEICC_28, MEICC_31) >= 1) MEICC_Pain = 1.

EXECUTE.

*Sleep*.

COMPUTE MEICC_19 = 0.

EXECUTE.

IF ((dsq_19f >= 2) & (dsq_19s >= 2)) MEICC_19 = 1.

EXECUTE.

COMPUTE MEICC_20 = 0.

EXECUTE.

IF ((dsq_20f >= 2) & (dsq_20s >= 2)) MEICC_20 = 1.

EXECUTE.

COMPUTE MEICC_21 = 0.

EXECUTE.

IF ((dsq_21f >= 2) & (dsq_21s >= 2)) MEICC_21 = 1.

EXECUTE.

COMPUTE MEICC_22 = 0.

EXECUTE.

IF ((dsq_22f >= 2) & (dsq_22s >= 2)) MEICC_22 = 1.

EXECUTE.

COMPUTE MEICC_23 = 0.

EXECUTE.

IF ((dsq_23f >= 2) & (dsq_23s >= 2)) MEICC_23 = 1.

EXECUTE.

COMPUTE MEICC_24 = 0.

EXECUTE.

IF ((dsq_24f >= 2) & (dsq_24s >= 2)) MEICC_24 = 1.

EXECUTE.

COMPUTE MEICC_Sleep = 0.

EXECUTE.

IF(SUM(MEICC_19, MEICC_20, MEICC_21, MEICC_22, MEICC_23, MEICC_24) >= 1) MEICC_Sleep = 1.

EXECUTE.

*Neurosensory*.

COMPUTE MEICC_32 = 0.

EXECUTE.

IF ((dsq_32f >= 2) & (dsq_32s >= 2)) MEICC_32 = 1.

EXECUTE.

COMPUTE MEICC_33 = 0.

EXECUTE.

IF ((dsq_33f >= 2) & (dsq_33s >= 2)) MEICC_33 = 1.

EXECUTE.

COMPUTE MEICC_34 = 0.

EXECUTE.

IF ((dsq_34f >= 2) & (dsq_34s >= 2)) MEICC_34 = 1.

EXECUTE.

COMPUTE MEICC_35 = 0.

EXECUTE.

IF ((dsq_35f >= 2) & (dsq_35s >= 2)) MEICC_35 = 1.

EXECUTE.

COMPUTE MEICC_48 = 0.

EXECUTE.

IF ((dsq_48f >= 2) & (dsq_48s >= 2)) MEICC_48 = 1.

EXECUTE.

COMPUTE MEICC_Sensory = 0.

EXECUTE.

IF(SUM(MEICC_32, MEICC_33, MEICC_34, MEICC_35, MEICC_48) >= 1) MEICC_Sensory = 1.

EXECUTE.

**Neurological Impairments**.

COMPUTE MEICC_Neuro = 0.

EXECUTE.

IF(SUM(MEICC_Cognitive, MEICC_Pain, MEICC_Sleep, MEICC_Sensory) >= 3) MEICC_Neuro = 1.

EXECUTE.

**Immune, Gastrointestinal, Genitourinary Impairments**.

*Flu*.

COMPUTE MEICC_62 = 0.

EXECUTE.

IF ((dsq_62f >= 2) & (dsq_62s >= 2)) MEICC_62 = 1.

EXECUTE.

COMPUTE MEICC_63 = 0.

EXECUTE.

IF ((dsq_63f >= 2) & (dsq_63s >= 2)) MEICC_63 = 1.

EXECUTE.

COMPUTE MEICC_64 = 0.

EXECUTE.

IF ((dsq_64f >= 2) & (dsq_64s >= 2)) MEICC_64 = 1.

EXECUTE.

COMPUTE MEICC_65 = 0.

EXECUTE.

IF ((dsq_65f >= 2) & (dsq_65s >= 2)) MEICC_65 = 1.

EXECUTE.

COMPUTE MEICC_Flu = 0.

EXECUTE.

IF(SUM(MEICC_62, MEICC_63, MEICC_64, MEICC_65) >= 1) MEICC_Flu = 1.

EXECUTE.

*Gastrointestinal*.

COMPUTE MEICC_29 = 0.

EXECUTE.

IF ((dsq_29f >= 2) & (dsq_29s >= 2)) MEICC_29 = 1.

EXECUTE.

COMPUTE MEICC_30 = 0.

EXECUTE.

IF ((dsq_30f >= 2) & (dsq_30s >= 2)) MEICC_30 = 1.

EXECUTE.

COMPUTE MEICC_46 = 0.

EXECUTE.

IF ((dsq_46f >= 2) & (dsq_46s >= 2)) MEICC_46 = 1.

EXECUTE.

COMPUTE MEICC_47 = 0.

EXECUTE.

IF ((dsq_47f >= 2) & (dsq_47s >= 2)) MEICC_47 = 1.

EXECUTE.

COMPUTE MEICC_Gastro = 0.

EXECUTE.

IF(SUM(MEICC_29, MEICC_30, MEICC_46, MEICC_47) >= 1) MEICC_Gastro = 1.

EXECUTE.

*Genitourinary*.

COMPUTE MEICC_Urinary = 0.

EXECUTE.

IF ((dsq_45f >= 2) & (dsq_45s >= 2)) MEICC_Urinary = 1.

EXECUTE.

*Sensitivities*.

COMPUTE MEICC_61 = 0.

EXECUTE.

IF ((dsq_61f >= 2) & (dsq_61s >= 2)) MEICC_61 = 1.

EXECUTE.

COMPUTE MEICC_66 = 0.

EXECUTE.

IF ((dsq_66f >= 2) & (dsq_66s >= 2)) MEICC_66 = 1.

EXECUTE.

COMPUTE MEICC_Sensitivity = 0.

EXECUTE.

IF(SUM(MEICC_61, MEICC_66) >= 1) MEICC_Sensitivity = 1.

EXECUTE.

*Virus Susceptibility*.

COMPUTE MEICC_Viral = 0.

EXECUTE.

IF(dsq_98 = 1) MEICC_Viral = 1.

EXECUTE.

**Immune, Gastrointestinal, Genitourinary Impairments**.

COMPUTE MEICC_IGG = 0.

EXECUTE.

IF(SUM(MEICC_Flu, MEICC_Gastro, MEICC_Urinary, MEICC_Sensitivity, MEICC_Viral) >= 3) MEICC_IGG = 1.

EXECUTE.

**Energy Metabolism, Ion Transport Impairments**.

*Cardiovascular*

COMPUTE MEICC_50 = 0.

EXECUTE.

IF ((dsq_50f >= 2) & (dsq_50s >= 2)) MEICC_50 = 1.

EXECUTE.

COMPUTE MEICC_51 = 0.

EXECUTE.

IF ((dsq_51f >= 2) & (dsq_51s >= 2)) MEICC_51 = 1.

EXECUTE.

COMPUTE MEICC_Cardio = 0.

EXECUTE.

IF(SUM(MEICC_50, MEICC_51) >= 1) MEICC_Cardio = 1.

EXECUTE.

*Respiratory*.

COMPUTE MEICC_Resp = 0.

EXECUTE.

IF((dsq_49f >= 2) & (dsq_49s >= 2)) MEICC_Resp = 1.

EXECUTE.

*Thermostatic Ability*.

COMPUTE MEICC_54 = 0.

EXECUTE.

IF ((dsq_54f >= 2) & (dsq_54s >= 2)) MEICC_54 = 1.

EXECUTE.

COMPUTE MEICC_55 = 0.

EXECUTE.

IF ((dsq_55f >= 2) & (dsq_55s >= 2)) MEICC_55 = 1.

EXECUTE.

COMPUTE MEICC_56 = 0.

EXECUTE.

IF ((dsq_56f >= 2) & (dsq_56s >= 2)) MEICC_56 = 1.

EXECUTE.

COMPUTE MEICC_57 = 0.

EXECUTE.

IF ((dsq_57f >= 2) & (dsq_57s >= 2)) MEICC_57 = 1.

EXECUTE.

COMPUTE MEICC_58 = 0.

EXECUTE.

IF ((dsq_58f >= 2) & (dsq_58s >= 2)) MEICC_58 = 1.

EXECUTE.

COMPUTE MEICC_59 = 0.

EXECUTE.

IF ((dsq_59f >= 2) & (dsq_59s >= 2)) MEICC_59 = 1.

EXECUTE.

COMPUTE MEICC_60 = 0.

EXECUTE.

IF ((dsq_60f >= 2) & (dsq_60s >= 2)) MEICC_60 = 1.

EXECUTE.

COMPUTE MEICC_Thermo = 0.

EXECUTE.

IF(SUM(MEICC_54, MEICC_55, MEICC_56, MEICC_57, MEICC_58, MEICC_59, MEICC_60) >= 1) MEICC_Thermo = 1.

EXECUTE.

*Temperature Intolerance*.

COMPUTE MEICC_Temp = 0.

EXECUTE.

IF(dsq_99 = 1) MEICC_Temp = 1.

EXECUTE.

**Immune, Gastrointestinal, Genitourinary Impairments**.

COMPUTE MEICC_Energy = 0.

EXECUTE.

IF(SUM(MEICC_Cardio, MEICC_Resp, MEICC_Thermo, MEICC_Temp) >= 1) MEICC_Energy = 1.

EXECUTE.

***ME-ICC Case Definition***.

COMPUTE MEICC = 0.

EXECUTE.

IF(SUM(MEICC_SR, MEICC_PENE, MEICC_Neuro, MEICC_IGG, MEICC_Energy) = 5) MEICC = 1.

EXECUTE.

VALUE LABELS

MEICC

0 ‘Does Not Meet ME-ICC’

1 ‘Meets ME-ICC’.

EXECUTE.

*****************************************************************************.

*DSQ-1*.

*Institute of Medicine Criteria (IOM, 2015)*.

*****************************************************************************.

***Substantial Reduction in Functioning Criteria, SF-36***.

***To score the SF-36, use scoring rules found here:

***https://www.rand.org/health/surveys_tools/mos/36-item-short-form/scoring.html***.

***Variable names are equivalent to SF-36 Subscale Names***.

COMPUTE IOM_RP = 0.

EXECUTE.

IF (RolePhysical <= 50) IOM_RP = 1.

EXECUTE.

COMPUTE IOM_SF = 0.

EXECUTE.

IF (SocialFunctioning <= 62.5) IOM_SF = 1.

EXECUTE.

COMPUTE IOM_V = 0.

EXECUTE.

IF (Vitality <= 35) IOM_V = 1.

EXECUTE.

COMPUTE IOM_SR=0.

EXECUTE.

IF (SUM(IOM_RP, IOM_SF, IOM_V) >= 2) IOM_SR = 1.

EXECUTE.

***DSQ-1 Symptom Scoring***.

*Fatigue, Not Lifelong, Not Result of Exertion*.

COMPUTE IOM_Six = 0.

EXECUTE.

IF ((dsq_69 = 2) | (dsq_69 = 3) | (dsq_69 = 4) | (dsq_69 = 5)) IOM_Six = 1.

EXECUTE.

COMPUTE IOM_Life = 0.

EXECUTE.

IF ((dsq_67 = 1) & (dsq_69 = 5) & (dsq_77 = 7)) IOM_Life = 1.

EXECUTE.

COMPUTE IOM_Exert = 0.

EXECUTE.

IF((SUM(dsq_89a, dsq_89d) >= 60)) IOM_Exert = 1.

EXECUTE.

COMPUTE IOM_Fatigue = 0.

EXECUTE.

IF((IOM_Six = 1) & (IOM_Life = 0) & (IOM_Exert = 0)) IOM_Fatigue = 1.

EXECUTE.

*Post-Exertional Malaise*.

COMPUTE IOM_14 = 0.

EXECUTE.

IF ((dsq_14f >= 2) & (dsq_14s >= 2)) IOM_14 = 1.

EXECUTE.

COMPUTE IOM_15 = 0.

EXECUTE.

IF ((dsq_15f >= 2) & (dsq_15s >= 2)) IOM_15 = 1.

EXECUTE.

COMPUTE IOM_16 = 0.

EXECUTE.

IF ((dsq_16f >= 2) & (dsq_16s >= 2)) IOM_16 = 1.

EXECUTE.

COMPUTE IOM_17 = 0.

EXECUTE.

IF ((dsq_17f >= 2) & (dsq_17s >= 2)) IOM_17 = 1.

EXECUTE.

COMPUTE IOM_18 = 0.

EXECUTE.

IF ((dsq_18f >= 2) & (dsq_18s >= 2)) IOM_18 = 1.

EXECUTE.

COMPUTE IOM_PEM = 0.

EXECUTE.

IF(SUM(IOM_14, IOM_15, IOM_16, IOM_17, IOM_18) >= 1) IOM_PEM = 1.

EXECUTE.

*Unrefreshing Sleep*.

COMPUTE IOM_19 = 0.

EXECUTE.

IF ((dsq_19f >= 2) & (dsq_19s >= 2)) IOM_19 = 1.

EXECUTE.

COMPUTE IOM_20 = 0.

EXECUTE.

IF ((dsq_20f >= 2) & (dsq_20s >= 2)) IOM_20 = 1.

EXECUTE.

COMPUTE IOM_21 = 0.

EXECUTE.

IF ((dsq_21f >= 2) & (dsq_21s >= 2)) IOM_21 = 1.

EXECUTE.

COMPUTE IOM_22 = 0.

EXECUTE.

IF ((dsq_22f >= 2) & (dsq_22s >= 2)) IOM_22 = 1.

EXECUTE.

COMPUTE IOM_24 = 0.

EXECUTE.

IF ((dsq_24f >= 2) & (dsq_24s >= 2)) IOM_24 = 1.

EXECUTE.

COMPUTE IOM_Sleep = 0.

EXECUTE.

IF(SUM(IOM_19, IOM_20, IOM_21, IOM_22, IOM_24) >= 1) IOM_Sleep = 1.

EXECUTE.

*Cognitive Impairment*.

COMPUTE IOM_36 = 0.

EXECUTE.

IF ((dsq_36f >= 2) & (dsq_36s >= 2)) IOM_36 = 1.

EXECUTE.

COMPUTE IOM_37 = 0.

EXECUTE.

IF ((dsq_37f >= 2) & (dsq_37s >= 2)) IOM_37 = 1.

EXECUTE.

COMPUTE IOM_38 = 0.

EXECUTE.

IF ((dsq_38f >= 2) & (dsq_38s >= 2)) IOM_38 = 1.

EXECUTE.

COMPUTE IOM_39 = 0.

EXECUTE.

IF ((dsq_39f >= 2) & (dsq_39s >= 2)) IOM_39 = 1.

EXECUTE.

COMPUTE IOM_40 = 0.

EXECUTE.

IF ((dsq_40f >= 2) & (dsq_40s >= 2)) IOM_40 = 1.

EXECUTE.

COMPUTE IOM_43 = 0.

EXECUTE.

IF ((dsq_43f >= 2) & (dsq_43s >= 2)) IOM_43 = 1.

EXECUTE.

COMPUTE IOM_44 = 0.

EXECUTE.

IF ((dsq_44f >= 2) & (dsq_44s >= 2)) IOM_44 = 1.

EXECUTE.

COMPUTE IOM_Cognitive = 0.

EXECUTE.

IF(SUM(IOM_36, IOM_37, IOM_38, IOM_39, IOM_40, IOM_43, IOM_44) >= 1) IOM_Cognitive = 1.

EXECUTE.

*Orthostatic Intolerance*.

COMPUTE IOM_48 = 0.

EXECUTE.

IF ((dsq_48f >= 2) & (dsq_48s >= 2)) IOM_48 = 1.

EXECUTE.

COMPUTE IOM_49 = 0.

EXECUTE.

IF ((dsq_49f >= 2) & (dsq_49s >= 2)) IOM_49 = 1.

EXECUTE.

COMPUTE IOM_50 = 0.

EXECUTE.

IF ((dsq_50f >= 2) & (dsq_50s >= 2)) IOM_50 = 1.

EXECUTE.

COMPUTE IOM_51 = 0.

EXECUTE.

IF ((dsq_51f >= 2) & (dsq_51s >= 2)) IOM_51 = 1.

EXECUTE.

COMPUTE IOM_OI = 0.

EXECUTE.

IF(SUM(IOM_48, IOM_49, IOM_50, IOM_51) >= 1) IOM_OI = 1.

EXECUTE.

***IOM Case Definition***.

COMPUTE IOM = 0.

EXECUTE.

IF((SUM(IOM_SR, IOM_Fatigue, IOM_PEM, IOM_Sleep) = 4) & (SUM(IOM_Cognitive, IOM_OI) >= 1)) IOM = 1.

EXECUTE.

VALUE LABELS

IOM

0 ‘Does Not Meet IOM Criteria’

1 ‘Meets IOM Criteria’.

EXECUTE.

ID# ____________________ Date_____________________

**DePaul Symptom Questionnaire (DSQ-1)**

1. What is your height?

2. What is your weight?

3. What is your date of birth?

4. What is your gender?

5. To which of the following race(s) do you belong?

 Black, African-American

 White

 American Indian or Alaska Native

 Asian or Pacific Islander

 Other race (*Please specify*)

6. Are you of Latino or Hispanic origin?

 Yes  No

7. What is your current marital status?

 Married or living with partner

 Separated

 Widowed

 Divorced

 Never married

8. Do you have any children?

 Yes  No (*Skip to Question 9)*

8a. How many children do you have?

8b. How many of your children are under 18 years old?

9. How many people live in your home?

10. What grade or degree have you completed in school?

 Less than high school

 Some high school

 High school degree or GED

 Partial college (at least one year) or specialized training

 Standard college degree

 Graduate professional degree including masters and doctorate

11. What is your current work status? **(Check all that apply)**

 On disability

 Student

 Homemaker

 Retired

 Unemployed

 Working part-time

 Working full-time

11a. If you are on disability, for what condition do you receive disability compensation?

Please Specify

12. What is your current occupation?

Current

12a. If you are currently not working, what was your most recent occupation?

Most Recent

For the following questions (13-66), we would like to know **how often you have had each symptom** and **how much each symptom has bothered you over the last 6 months**. For each symptom please circle **one number for frequency** **and** **one number for severity**. Please fill the chart out from left to right.

| **Symptoms** | *Frequency:*  Throughout the **past 6 months**, **how often** have you had this symptom?  For each symptom listed below, circle a number from:  **0 = none of the time**  **1 = a little of the time**  **2 = about half the time**  **3 = most of the time**  **4 = all of the time** | *Severity:*  Throughout the **past 6 months**, **how much** has this symptom bothered you?  For each symptom listed below, circle a number from:  **0 = symptom not present**  **1 = mild**  **2 = moderate**  **3 = severe**  **4 = very severe** |
| --- | --- | --- |
| **13) Fatigue/extreme tiredness** | 0 1 2 3 4 | 0 1 2 3 4 |
| **14) Dead, heavy feeling after starting to exercise** | 0 1 2 3 4 | 0 1 2 3 4 |
| **15) Next day soreness or fatigue after non-strenuous, everyday activities** | 0 1 2 3 4 | 0 1 2 3 4 |
| **16)** **Mentally tired after the slightest effort** | 0 1 2 3 4 | 0 1 2 3 4 |
| **17) Minimum exercise makes you physically tired** | 0 1 2 3 4 | 0 1 2 3 4 |
| **18) Physically drained or sick after mild activity** | 0 1 2 3 4 | 0 1 2 3 4 |
| **19) Feeling unrefreshed after you wake up in the morning** | 0 1 2 3 4 | 0 1 2 3 4 |
| **20) Need to nap daily** | 0 1 2 3 4 | 0 1 2 3 4 |
| **21) Problems falling asleep** | 0 1 2 3 4 | 0 1 2 3 4 |
| **22) Problems staying asleep** | 0 1 2 3 4 | 0 1 2 3 4 |
| **23) Waking up early in the morning (e.g. 3am)** | 0 1 2 3 4 | 0 1 2 3 4 |
| **24) Sleep all day and stay awake all night** | 0 1 2 3 4 | 0 1 2 3 4 |
| **25) Pain or aching in your muscles** | 0 1 2 3 4 | 0 1 2 3 4 |
| **26) Pain/stiffness/tenderness in more than one joint without swelling or redness** | 0 1 2 3 4 | 0 1 2 3 4 |
| **27) Eye pain** | 0 1 2 3 4 | 0 1 2 3 4 |
| **Symptoms** | *Frequency:*  Throughout the **past 6 months**, **how often** have you had this symptom?  For each symptom listed below, circle a number from:  **0 = none of the time**  **1 = a little of the time**  **2 = about half the time**  **3 = most of the time**  **4 = all of the time** | *Severity:*  Throughout the **past 6 months**, **how much** has this symptom bothered you?  For each symptom listed below, circle a number from:  **0 = symptom not present**  **1 = mild**  **2 = moderate**  **3= severe**  **4 = very severe** |
| **28) Chest pain** | 0 1 2 3 4 | 0 1 2 3 4 |
| **29) Bloating** | 0 1 2 3 4 | 0 1 2 3 4 |
| **30) Abdomen/stomach pain** | 0 1 2 3 4 | 0 1 2 3 4 |
| **31) Headaches** | 0 1 2 3 4 | 0 1 2 3 4 |
| **32) Muscle twitches** | 0 1 2 3 4 | 0 1 2 3 4 |
| **33) Muscle weakness** | 0 1 2 3 4 | 0 1 2 3 4 |
| **34) Sensitivity to noise** | 0 1 2 3 4 | 0 1 2 3 4 |
| **35) Sensitivity to bright lights** | 0 1 2 3 4 | 0 1 2 3 4 |
| **36) Problems remembering things** | 0 1 2 3 4 | 0 1 2 3 4 |
| **37) Difficulty paying attention for a long period of time** | 0 1 2 3 4 | 0 1 2 3 4 |
| **38) Difficulty finding the right word to say or expressing thoughts** | 0 1 2 3 4 | 0 1 2 3 4 |
| **39) Difficulty understanding things** | 0 1 2 3 4 | 0 1 2 3 4 |
| **40) Only able to focus on one thing at a time** | 0 1 2 3 4 | 0 1 2 3 4 |
| **41) Unable to focus vision and/or attention** | 0 1 2 3 4 | 0 1 2 3 4 |
| **42) Loss of depth perception** | 0 1 2 3 4 | 0 1 2 3 4 |
| **43) Slowness of thought** | 0 1 2 3 4 | 0 1 2 3 4 |
| **44) Absent-mindedness or forgetfulness** | 0 1 2 3 4 | 0 1 2 3 4 |
| **45) Bladder problems** | 0 1 2 3 4 | 0 1 2 3 4 |
| **46) Irritable bowel problems** | 0 1 2 3 4 | 0 1 2 3 4 |
| **Symptoms** | *Frequency:*  Throughout the **past 6 months**, **how often** have you had this symptom?  For each symptom listed below, circle a number from:  **0 = none of the time**  **1 = a little of the time**  **2 = about half the time**  **3 = most of the time**  **4 = all of the time** | *Severity:*  Throughout the **past 6 months**, **how much** has this symptom bothered you?  For each symptom listed below, circle a number from:  **0 = symptom not present**  **1 = mild**  **2 = moderate**  **3= severe**  **4 = very severe** |
| **47) Nausea** | 0 1 2 3 4 | 0 1 2 3 4 |
| **48) Feeling unsteady on your feet, like you might fall** | 0 1 2 3 4 | 0 1 2 3 4 |
| **49) Shortness of breath or trouble catching your breath** | 0 1 2 3 4 | 0 1 2 3 4 |
| **50) Dizziness or fainting** | 0 1 2 3 4 | 0 1 2 3 4 |
| **51) Irregular heart beats** | 0 1 2 3 4 | 0 1 2 3 4 |
| **52) Losing or gaining weight without trying** | 0 1 2 3 4 | 0 1 2 3 4 |
| **53) No appetite** | 0 1 2 3 4 | 0 1 2 3 4 |
| **54) Sweating hands** | 0 1 2 3 4 | 0 1 2 3 4 |
| **55) Night sweats** | 0 1 2 3 4 | 0 1 2 3 4 |
| **56) Cold limbs (e.g. arms, legs, hands)** | 0 1 2 3 4 | 0 1 2 3 4 |
| **57) Feeling chills or shivers** | 0 1 2 3 4 | 0 1 2 3 4 |
| **58) Feeling hot or cold for no reason** | 0 1 2 3 4 | 0 1 2 3 4 |
| **59) Feeling like you have a high temperature** | 0 1 2 3 4 | 0 1 2 3 4 |
| **60) Feeling like you have a low temperature** | 0 1 2 3 4 | 0 1 2 3 4 |
| **61) Alcohol intolerance** | 0 1 2 3 4 | 0 1 2 3 4 |
| **62) Sore throat** | 0 1 2 3 4 | 0 1 2 3 4 |
| **63) Tender/sore lymph nodes** | 0 1 2 3 4 | 0 1 2 3 4 |
| **64) Fever** | 0 1 2 3 4 | 0 1 2 3 4 |
| **65) Flu-like symptoms** | 0 1 2 3 4 | 0 1 2 3 4 |
| **66) Some smells, foods, medications, or chemicals make you feel sick** | 0 1 2 3 4 | 0 1 2 3 4 |

67. Have you **always** had persistent or recurring **fatigue/energy problems**, even back to your earliest memories as a child? (By persistent or recurring, we mean that the fatigue/energy problems are usually ongoing and constant, but sometimes there are good periods and bad periods.)

 Yes  No  Not having a problem with fatigue/energy

68. Since your **fatigue/energy related illness** began, do your headaches either happen more often, feel worse or more severe, or are they in a different place or spot?

 Yes  No  Not having a problem with fatigue/energy

69. How long ago did your problem with **fatigue/energy** begin?

 Less than 6 months

 6-12 months

 1-2 years

 Longer than 2 years

 Had problem with fatigue/energy since childhood or adolescence

 Not having a problem with fatigue/energy

70. Have you been diagnosed with Chronic Fatigue Syndrome or Myalgic Encephalomyelitis?

 Yes  No

70a. If yes, what year were you diagnosed?

70b. Do you currently have a diagnosis of Chronic Fatigue Syndrome or Myalgic Encephalomyelitis?

 Yes  No

70c. Who diagnosed you with Chronic Fatigue Syndrome or Myalgic Encephalomyelitis?

 Medical Doctor  Alternative Practitioner  Self-Diagnosed

70d. Have any of your family members been diagnosed with Chronic Fatigue Syndrome or Myalgic Encephalomyelitis?

 Yes  No

If yes, please list their relation to you and current age

71. Did you experience any of the following symptoms regularly and repeatedly in the months and years before your fatigue/energy problems began?

 Sore throat

 Tender/sore lymph nodes

 Unrefreshing sleep

 Impaired memory and concentration

 Prolonged fatigue following physical or mental exertion

 Muscle pain

 Headaches

 Joint Pain

 Not having a problem with fatigue/energy

72. If you rest, does your problem with **fatigue/energy** go away? **(Check one)**

 Entirely

 Partially

 My fatigue/energy problem is not improved by rest *(Skip to Question 73)*

 I am not having a problem with fatigue/energy *(Skip to Question 73)*

72a. How long do you have to rest for your problem with **fatigue/energy** to entirely or partially go away?

 less than 30 minutes  30 to 59 minutes  1-2 hours  more than 2 hours

73. If you were to become exhausted after actively participating in extracurricular activities, sports, or outings with friends, would you recover within an hour or two after the activity ended?

 Yes  No

74. Do you reduce your activity level to avoid experiencing problems with **fatigue/energy**?

 Yes  No  Not having a problem with fatigue/energy

75. Do you experience a worsening of your **fatigue/energy related illness** after engaging in minimal physical effort?

Yes  No  Not having a problem with fatigue/energy

75a. Do you experience a worsening of your **fatigue/energy related illness** after engaging in mental effort?

 Yes  No

75b. If you feel worse after activities, how long does this last? **(Check one)**

 1 hour or less  2-3 Hrs  4-10 Hrs  11-13 Hrs

 14-23 Hrs  More than 24 Hrs (Please specify__________)

76. Are you currently engaging in any form of exercise?

 Yes *(Skip to Question 77)*  No

76a. If you do not exercise, why aren’t you exercising? **(Check all boxes that you agree with)**

 Not interested

 No time

 Would like to but cannot because of problems with fatigue/energy

 Cannot because exercise makes symptoms worse

77. Over what period of time did your **fatigue/energy related illness,** develop? **(Check one)**

 Within 24 hours

 Over 1 week

 Over 1 month

 Over 2-6 months

 Over 7-12 months

 Over 1-2 years

 Over 3 or more years

 I am not ill

78. How would you describe the course of your **fatigue/energy related illness?** **(Check one)**

 Constantly getting worse

 Constantly improving

 Persisting (no change)

 Relapsing & remitting (having “good” periods with no symptoms & “bad” periods)

 Fluctuating (symptoms periodically get better and get worse, but never disappear completely)

 No Symptoms/I am not ill

79. Which statement best describes your **fatigue/energy related illness** during the **last 6**  **months**? **(Check one)**

 I am not able to work or do anything, and I am bedridden.

 I can walk around the house, but I cannot do light housework.

 I can do light housework, but I cannot work part-time.

 I can only work part-time at work or on some family responsibilities.

 I can work full time, but I have no energy left for anything else.

 I can work full time and finish some family responsibilities but I have no energy left

for anything else.

 I can do all work or family responsibilities without any problems with my energy.

80. Did your **fatigue/energy related illness** start after you experienced any of the following? **(Check one or more and please specify)**

 An infectious illness

 An accident

 A trip or vacation

 An immunization (shot at doctor’s office)

 Surgery

 Severe stress (bad or unhappy event(s))

 Other

 I am not ill

81. Have you ever consulted a medical doctor or health professional about your **fatigue/energy** problem?

 Yes  No *(Skip to Question 83)*

82. Do you currently have a medical doctor overseeing your **fatigue/energy** problem?

 Yes  No

83. Do you have any medical illness (es) that might be causing your symptoms?

 Yes  No *(Skip to Question 84)*

83a. What medical illnesses do you have?

Illness name(s) and year it began:

83b. For which of these conditions are you currently receiving treatment?

84. Are you currently taking any medications (over the counter or prescription)?

 Yes  No *(Skip to Question 86)*

84a. What medications are you taking?

85. Do you think any medication(s) is (are) causing your problem with **fatigue/energy**?

 Yes  No *(Skip to Question 86)*

 I do not have a problem with fatigue/energy *(Skip to Question 86)*

85a. Please specify which medications:

86. Have you ever been diagnosed and/or treated for any of the following: **(Check all that apply and write year (s) experienced, years treated, and medication (if applicable) in the blank)**

 Major depression

 Major depression with melancholic or psychotic features

 Bipolar disorder (Manic-depression)

 Anxiety

 Schizophrenia

 Eating disorder

 Substance abuse

 Multiple chemical sensitivities

 Fibromyalgia

 Allergies

 Other (*Please specify*)

 No diagnosis/treatment

87. What do you think is the cause of your problem with **fatigue/energy**? **(Check one)**

 Definitely physical

 Mainly physical

 Equally physical and psychological

 Mainly psychological

 Definitely psychological

 No problem with fatigue/energy

88. Do you think anything specific in your personal life or environment accounts for your problem with **fatigue/energy**?

 Yes  No *(Skip to Question 89)*

 I do not have a problem with fatigue/energy (*Skip to Question 89)*

88a. Please specify:

89. In the **past 4 weeks**, approximately how many hours per week have you spent doing:

Household related activities? hours per week

Social/Recreational related activities? hours per week

Family related activities? hours per week

Work related activities? hours per week

90. In the **past** **4 weeks**, have you had to reduce the number of hours you previously spent (prior to your illness) on occupational, social or family activities because of your health or problems with **fatigue/energy**?

 Yes  No *(Skip to Question 91)*  Not having a problem with fatigue/energy

90a. **Before your fatigue/energy related illness**, approximately how many hours did you used to spend on:

Household related activities? hours per week

Social/Recreational related activities? hours per week

Family related activities? hours per week

Work related activities? hours per week

91. Please rate the amount of **energy** you had available **yesterday**, using a scale from 1 to 100 where 1= no energy and 100 = your pre-illness energy level. **(If you don't have a fatigue/energy related illness, a score of 100 = having abundant energy such that you could work full time and complete your family responsibilities)**

92. Please rate the amount of **energy** you expended (used) **yesterday**, using a scale from 1 to 100 where 1 = no energy and 100 = your pre-illness energy expended

93. Please rate the amount of **fatigue** you had **yesterday**, using a scale from 1 to 100 where 1 = no fatigue and 100 = severe fatigue

94. For the **past week**, please rate the amount of **energy** you had available using a scale from 1 to 100 where 1 = no energy and 100 = your pre-illness energy level

95. For the **past week**, please rate the amount of **energy** you have expended (used) using a scale from 1 to 100 where 1 = no energy and 100 = your pre-illness energy expended

96. For the **past week**, please rate the amount of **fatigue** you have had using a scale from 1 to 100 where 1 = no fatigue and 100 = severe fatigue

97. Since the onset of your problems with fatigue/energy, have your symptoms caused a 50% or greater reduction in your activity level?

 Yes  No  Not having a problem with fatigue/energy

98. Do you experience frequent viral infections with prolonged recovery periods?

 Yes  No

99. Are you intolerant of extremes of temperatures (when it is extremely hot or cold)?

 Yes  No

**To Measure Substantial Reduction Requirement in the Case Definitions**

**MOS SURVEY (SF-36)**

**INSTRUCTIONS:**

This survey asks for your views about your health. This information will help keep track of how you feel and

how well you are able to do your usual activities. Answer every question by marking the answer as

indicated. If you are unsure about how to answer a question, please give the best answer you can.

1. In general, would you say your health is: ***(Please circle one)***

Excellent 1

Very good 2

Good 3

Fair 4

Poor 5

2. **Compared to one year ago,** how would you rate your health in general now? ***(Please circle one)***

Much better than one year ago 1

Somewhat better now than one year ago 2

About the same as one year ago 3

Somewhat worse now than one year ago 4

Much worse now than one year ago 5

3. The following items are about activities you might do during a typical day. Does your health now

limit you in these activities? If so, how much?

| **Activities** | **Yes,**  **Limited**  **A Lot** | **Yes,  Limited**  **A Little** | **No, Not**  **Limited**  **At All** |
| --- | --- | --- | --- |
| **Vigorous activities**: running, lifting heavy objects, participating in strenuous sports | 1 | 2 | 3 |
| **Moderate activities**: moving a table, pushing a vacuum cleaner, bowling, playing golf | 1 | 2 | 3 |
| Lifting or carrying groceries | 1 | 2 | 3 |
| Climbing **several** flights of stairs | 1 | 2 | 3 |
| Climbing **one** flight of stairs | 1 | 2 | 3 |
| Bending, kneeling, or stooping | 1 | 2 | 3 |
| Walking **more than a mile** | 1 | 2 | 3 |
| Walking **several blocks** | 1 | 2 | 3 |
| Walking **one** block | 1 | 2 | 3 |
| Bathing or dressing yourself | 1 | 2 | 3 |

4. During the **past 4 weeks**, have you had any of the following problems with your work or other

regular daily activities as a result of your **physical health**?

| **Problems** | **Yes** | **No** |
| --- | --- | --- |
| Cut down on the  **amount of time** you spent on work or other activities | 1 | 2 |
| **Accomplished less** than you would like | 1 | 2 |
| Were limited in the **kind** of work or other activities | 1 | 2 |
| Had **difficulty** performing the work or other activities (For example, it took extra effort) | 1 | 2 |

5. During the **past 4 weeks**, have you had any of the following problems with your work or other

regular daily activities **as a result of any emotional problems** (such as feeling depressed or anxious)?

| **Problems** | **Yes** | **No** |
| --- | --- | --- |
| Cut down the **amount of time** you spent on work or other activities | 1 | 2 |
| **Accomplished less** than you would like | 1 | 2 |
| Didn’t do work or other activities as **carefully** as usual | 1 | 2 |

6. During the **past 4 weeks**, to what extent has your physical health or emotional problems interfered with

your normal social activities with family, neighbors, or groups? ***(Please circle one)***

Not at all 1

Slightly 2

Moderately 3

Quite a bit 4

Extremely 5

7. How much bodily pain have you had during the **past 4 weeks**?

None 1

Very mild 2

Mild 3

Moderate 4

Severe 5

Very Severe 6

8. During the **past 4 weeks**, how much did pain interfere with your normal work (including both work

outside the home and housework)?

Not at all 1

Slightly 2

Moderately 3

Quite a bit 4

Extremely 5

9. These questions are about how you feel and how things have been with you **during the past 4 weeks**.

For each question, please give the one answer that comes closest to the way you have been feeling. How much of the time **during the past 4 weeks**-

| **Questions** | **All**  **of the**  **Time** | **Most**  **of the**  **Time** | **A Good**  **Bit of**  **the Time** | **Some**  **of the**  **Time** | **A Little**  **of the**  **Time** | **None**  **of the**  **Time** |
| --- | --- | --- | --- | --- | --- | --- |
| Did you feel full of pep? | 1 | 2 | 3 | 4 | 5 | 6 |
| Have you been a nervous person? | 1 | 2 | 3 | 4 | 5 | 6 |
| Have you felt so down in the dumps that nothing could cheer you up? | 1 | 2 | 3 | 4 | 5 | 6 |
| Have you felt calm and peaceful? | 1 | 2 | 3 | 4 | 5 | 6 |
| Did you have a lot of energy? | 1 | 2 | 3 | 4 | 5 | 6 |
| Have you felt down-hearted and blue? | 1 | 2 | 3 | 4 | 5 | 6 |
| Did you feel worn out? | 1 | 2 | 3 | 4 | 5 | 6 |
| Have you been a happy person? | 1 | 2 | 3 | 4 | 5 | 6 |
| Did you feel tired? | 1 | 2 | 3 | 4 | 5 | 6 |

10. During the **past 4 weeks**, how much of the time has your physical health or

emotional problems interfered with your social activities (like visiting with friends,

relatives, etc.)?

All of the time 1

Most of the time 2

Some of the time 3

A little of the time 4

None of the time 5

11. How **TRUE** or **FALSE** is each of following statements for you?

| **Statements** | **Definitely**  **True** | **Mostly**  **True** | **Don’t**  **Know** | **Mostly**  **False** | **Definitely**  **False** |
| --- | --- | --- | --- | --- | --- |
| I seem to get sick a little easier than other people | 1 | 2 | 3 | 4 | 5 |
| I am as healthy as anybody I know | 1 | 2 | 3 | 4 | 5 |
| I expect my health to get worse | 1 | 2 | 3 | 4 | 5 |
| My health is excellent | 1 | 2 | 3 | 4 | 5 |
