# Supplementary material for: The Development of the DePaul Symptom Questionnaire: Original, Expanded, Brief, and Pediatric Versions
Source: Front Pediatr. 2018 Nov 6;6:330. doi: 10.3389/fped.2018.00330 (PMC6232226; doi:10.3389/fped.2018.00330)
Supplement: Supplementary file 3 [file Data_Sheet_3.docx]

**Data Sheet 3.**

**DSQ-SF**

This document contains the following material:

1. Case definition scoring rules and associated symptoms for the following criteria:
   1. Fukuda et al. (1994)
   2. Canadian ME/CFS (Carruthers et al., 2003)
   3. Institute of Medicine (IOM, 2015)
2. Syntax for the following case definitions:
   1. Fukuda et al. (1994)
   2. Canadian ME/CFS (Carruthers et al., 2003)
   3. Institute of Medicine (IOM, 2015)
3. Hard copy of the DSQ-SF and the SF-36

The DePaul Symptom Questionnaire (DSQ-SF) can be downloaded from the REDCap shared library. You can view the instrument here:

<https://redcap.is.depaul.edu/surveys/?s=HCT7J8EWPC>

**DSQ-SF**

**Case Definition Criteria:**

**Fukuda (Fukuda et al., 1994)**

- Substantial Reduction in Functioning (must meet 2 of the following 3 SF-36 score cutoffs):
  - Role Physical <= 50
  - Social Functioning <= 62.5
  - Vitality <= 35
- 6+ months of fatigue (Question 1; frequency and severity ratings >=1)
- At least 1 symptom (frequency and severity ratings >=1) from at least 4 symptom domains:
  - Post-Exertional Malaise (Question 2 or 3)
  - Unrefreshing Sleep (Question 4)
  - Muscle Aches (Question 5)
  - Memory / Concentration (Question 7 or 8*)*
  - Flu-like symptoms (Question 13)

**Canadian Consensus Criteria (CCC Case Definition) (Carruthers et al., 2003)**

- Substantial Reduction in Functioning (must meet 2 of the following 3 SF-36 score cutoffs):
  - Role Physical <= 50
  - Social Functioning <= 62.5
  - Vitality <= 35
- At least one symptom (frequency and severity ratings >=2) from **all** of the following domains:
  - Fatigue (Question 1)
  - Post-Exertional Malaise (Question 2 or 3)
  - Sleep Problems (Question 4)
  - Neurological / Cognitive Problems (Question 7 or 8)
- At least one symptom (frequency and severity ratings >=2) from **one** of the following domains:
  - Pain (Question 5 or 6)
  - Autonomic (Question 9 or 10)
  - Neuroendocrine (Question 11 or 12)
  - Immune (Question 13 or 14)

**IOM Clinical Case Definition (IOM, 2015):**

- Substantial Reduction in Functioning (must meet 2 of the following 3 SF-36 score cutoffs):
  - Role Physical <= 50
  - Social Functioning <= 62.5
  - Vitality <= 35
- At least one symptom (frequency and severity ratings >=2) from **all** of the following domains:
  - Fatigue (Question 1)
  - Post-Exertional Malaise (Question 2 or 3)
  - Unrefreshing Sleep (Question 4)
- At least one symptom (frequency and severity ratings >=2) from **one** of the following domains:
  - Neurocognitive Problems (Question 7 or 8)
  - Orthostatic Intolerance (Question 10)

**DSQ-SF**

**SPSS Case Definition Syntax:**

**Scoring Note: To determine which variables are associated with which items, note that variable names utilize the items numbers present in the questionnaire*.*

********************************************************************************

*DSQ-SF*.

*Fukuda et al. (1994) Criteria*.

********************************************************************************.

***Substantial Reduction in Functioning Criteria, SF-36***.

***To score the SF-36, use scoring rules found here:

***https://www.rand.org/health/surveys_tools/mos/36-item-short-form/scoring.html***.

***Variable names are equivalent to SF-36 Subscale Names***.

COMPUTE SF_RP = 0.

EXECUTE.

IF (RolePhysical <= 50) SF_RP = 1.

EXECUTE.

COMPUTE SF_SF = 0.

EXECUTE.

IF (SocialFunctioning <= 62.5) SF_SF = 1.

EXECUTE.

COMPUTE SF_V = 0.

EXECUTE.

IF (Vitality <= 35) SF_V = 1.

EXECUTE.

COMPUTE SF_SR=0.

EXECUTE.

IF (SUM(SF_RP, SF_SF, SF_V) >= 2) SF_SR = 1.

EXECUTE.

***DSQ-SF Symptom Scoring***.

COMPUTE Fatigue_11 = 0.

EXECUTE.

IF((dsqsf_1f > 0) & (dsqsf_1s) > 0) Fatigue_11 = 1.

EXECUTE.

COMPUTE Soreness_11 = 0.

EXECUTE.

IF((dsqsf_2f > 0) & (dsqsf_2s > 0)) Soreness_11 = 1.

EXECUTE.

COMPUTE Minimum_11 = 0.

EXECUTE.

IF((dsqsf_3f > 0) & (dsqsf_3s > 0)) Minimum_11 = 1.

EXECUTE.

COMPUTE Unrefreshed_11 = 0.

EXECUTE.

IF((dsqsf_4f > 0) & (dsqsf_4s > 0)) Unrefreshed_11 = 1.

EXECUTE.

COMPUTE Musclepain_11 = 0.

EXECUTE.

IF((dsqsf_5f > 0) & (dsqsf_5s > 0)) Musclepain_11 = 1.

EXECUTE.

COMPUTE Remember_11 = 0.

EXECUTE.

IF((dsqsf_7f > 0) & (dsqsf_7s > 0)) Remember_11 = 1.

EXECUTE.

COMPUTE Attention_11 = 0.

EXECUTE.

IF((dsqsf_8f > 0) & (dsqsf_8s > 0)) Attention_11 = 1.

EXECUTE.

COMPUTE Flu_11 = 0.

EXECUTE.

IF((dsqsf_13f > 0) & (dsqsf_13s > 0)) Flu_11 = 1.

EXECUTE.

***Short Form Fukuda et al. (1994) Criteria***.

COMPUTE PEM_11 = 0.

EXECUTE.

IF(SUM(Soreness_11, Minimum_11) > 0) PEM_11 = 1.

EXECUTE.

COMPUTE Cognitive_11 = 0.

EXECUTE.

IF(SUM(Remember_11, Attention_11) > 0) Cognitive_11 = 1.

EXECUTE.

COMPUTE SF_Fukuda = 0.

EXECUTE.

IF ((Fatigue_11 = 1) & (SF_SR = 1) &

SUM(PEM_11, Cognitive_11, Unrefreshed_11, Musclepain_11, Flu_11) > 3)

SF_Fukuda = 1.

EXECUTE.

VALUE LABELS

SF_Fukuda

0 ‘Does Not Meet Fukuda Criteria’

1 ‘Meets Fukuda Criteria’

EXECUTE.

***End of DSQ-SF Fukuda et al. (1994) Criteria Syntax***.

********************************************************************************

*DSQ-SF*.

*Canadian ME/CFS Criteria (Carruthers et al., 2003)*.

********************************************************************************.

***Substantial Reduction in Functioning Criteria, SF-36***.

***To score the SF-36, use scoring rules found here:***.

***https://www.rand.org/health/surveys_tools/mos/36-item-short-form/scoring.html***.

***Variable names are equivalent to SF-36 Subscale Names***.

COMPUTE SF_RP = 0.

EXECUTE.

IF (RolePhysical <= 50) SF_RP = 1.

EXECUTE.

COMPUTE SF_SF = 0.

EXECUTE.

IF (SocialFunctioning <= 62.5) SF_SF = 1.

EXECUTE.

COMPUTE SF_V = 0.

EXECUTE.

IF (Vitality <= 35) SF_V = 1.

EXECUTE.

COMPUTE SF_SR=0.

EXECUTE.

IF (SUM(SF_RP, SF_SF, SF_V) >= 2) SF_SR = 1.

EXECUTE.

***DSQ-SF Symptom Scoring***.

COMPUTE Fatigue_22 = 0.

EXECUTE.

IF((dsqsf_1f > 0) & (dsqsf_1s) > 0) Fatigue_22 = 1.

EXECUTE.

COMPUTE Soreness_22 = 0.

EXECUTE.

IF((dsqsf_2f > 0) & (dsqsf_2s > 0)) Soreness_22 = 1.

EXECUTE.

COMPUTE Minimum_22 = 0.

EXECUTE.

IF((dsqsf_3f > 0) & (dsqsf_3s > 0)) Minimum_22 = 1.

EXECUTE.

COMPUTE Unrefreshed_22 = 0.

EXECUTE.

IF((dsqsf_4f > 0) & (dsqsf_4s > 0)) Unrefreshed_22 = 1.

EXECUTE.

COMPUTE Musclepain_22 = 0.

EXECUTE.

IF((dsqsf_5f > 0) & (dsqsf_5s > 0)) Musclepain_22 = 1.

EXECUTE.

COMPUTE Bloating_22 = 0.

EXECUTE.

IF((dsqsf_6f > 0) & (dsqsf_6s > 0)) Bloating_22 = 1.

EXECUTE.

COMPUTE Remember_22 = 0.

EXECUTE.

IF((dsqsf_7f > 0) & (dsqsf_7s > 0)) Remember_22 = 1.

EXECUTE.

COMPUTE Attention_22 = 0.

EXECUTE.

IF((dsqsf_8f > 0) & (dsqsf_8s > 0)) Attention_22 = 1.

EXECUTE.

COMPUTE Bowel_22 = 0.

EXECUTE.

IF((dsqsf_9f > 0) & (dsqsf_9s > 0)) Bowel_22 = 1.

EXECUTE.

COMPUTE Unsteady_22 = 0.

EXECUTE.

IF((dsqsf_10f > 0) & (dsqsf_10s > 0)) Unsteady_22 = 1.

EXECUTE.

COMPUTE Limbs_22 = 0.

EXECUTE.

IF((dsqsf_11f > 0) & (dsqsf_11s > 0)) Limbs_22 = 1.

EXECUTE.

COMPUTE HotCold_22 = 0.

EXECUTE.

IF((dsqsf_12f > 0) & (dsqsf_12s > 0)) HotCold_22 = 1.

EXECUTE.

COMPUTE Flu_22 = 0.

EXECUTE.

IF((dsqsf_13f > 0) & (dsqsf_13s > 0)) Flu_22 = 1.

EXECUTE.

COMPUTE Sensitivity_22 = 0.

EXECUTE.

IF((dsqsf_14f > 0) & (dsqsf_14s > 0)) Sensitivity_22 = 1.

EXECUTE.

***DSQ-SF Canadian ME/CFS (2003) Criteria.***

COMPUTE PEM_22 = 0.

EXECUTE.

IF(SUM(Soreness_22, Minimum_22) > 0) PEM_22 = 1.

EXECUTE.

COMPUTE Neurocog_22 = 0.

EXECUTE.

IF(SUM(Remember_22, Attention_22) > 0) Neurocog_22 = 1.

EXECUTE.

COMPUTE Pain_22 = 0.

EXECUTE.

IF(SUM(Musclepain_22, Bloating_22) > 0) Pain_22 = 1.

EXECUTE.

COMPUTE Autonomic_22 = 0.

EXECUTE.

IF(SUM(Bowel_22, Unsteady_22) > 0) Autonomic_22 = 1.

EXECUTE.

COMPUTE Neuroendo_22 = 0.

EXECUTE.

IF(SUM(Limbs_22, HotCold_22) > 0) Neuroendo_22 = 1.

EXECUTE.

COMPUTE Immune_22 = 0.

EXECUTE.

IF(SUM(Flu_22, Sensitivity_22) > 0) Immune_22 = 1.

EXECUTE.

COMPUTE SF_Canadian = 0.

EXECUTE.

IF((Fatigue_22 = 1) & (SF_SR = 1) & (PEM_22 = 1) & (Unrefreshed_22 = 1) & (Neurocog_22 = 1) & (SUM(Pain_22, Autonomic_22, Neuroendo_22, Immune_22) >= 1)) SF_Canadian = 1.

EXECUTE.

VALUE LABELS

SF_Canadian

0 ‘Does Not Meet Canadian Criteria’

1 ‘Meets Canadian Criteria’.

EXECUTE.

***End of DSQ-SF Canadian ME/CFS Criteria (Carruthers et al., 2003) Syntax***.

********************************************************************************

*DSQ-SF*.

*Institute of Medicine (IOM) Criteria (2015)*.

********************************************************************************.

***Substantial Reduction in Functioning Criteria, SF-36***.

***To score the SF-36, use scoring rules found here:***.

***https://www.rand.org/health/surveys_tools/mos/36-item-short-form/scoring.html***.

***Variable names are equivalent to SF-36 Subscale Names***.

COMPUTE SF_RP = 0.

EXECUTE.

IF (RolePhysical <= 50) SF_RP = 1.

EXECUTE.

COMPUTE SF_SF = 0.

EXECUTE.

IF (SocialFunctioning <= 62.5) SF_SF = 1.

EXECUTE.

COMPUTE SF_V = 0.

EXECUTE.

IF (Vitality <= 35) SF_V = 1.

EXECUTE.

COMPUTE SF_SR=0.

EXECUTE.

IF (SUM(SF_RP, SF_SF, SF_V) >= 2) SF_SR = 1.

EXECUTE.

***DSQ-SF Symptom Scoring***.

COMPUTE Fatigue_22 = 0.

EXECUTE.

IF((dsqsf_1f > 0) & (dsqsf_1s) > 0) Fatigue_22 = 1.

EXECUTE.

COMPUTE Soreness_22 = 0.

EXECUTE.

IF((dsqsf_2f > 0) & (dsqsf_2s > 0)) Soreness_22 = 1.

EXECUTE.

COMPUTE Minimum_22 = 0.

EXECUTE.

IF((dsqsf_3f > 0) & (dsqsf_3s > 0)) Minimum_22 = 1.

EXECUTE.

COMPUTE Unrefreshed_22 = 0.

EXECUTE.

IF((dsqsf_4f > 0) & (dsqsf_4s > 0)) Unrefreshed_22 = 1.

EXECUTE.

COMPUTE Remember_22 = 0.

EXECUTE.

IF((dsqsf_7f > 0) & (dsqsf_7s > 0)) Remember_22 = 1.

EXECUTE.

COMPUTE Attention_22 = 0.

EXECUTE.

IF((dsqsf_8f > 0) & (dsqsf_8s > 0)) Attention_22 = 1.

EXECUTE.

COMPUTE Unsteady_22 = 0.

EXECUTE.

IF((dsqsf_10f > 0) & (dsqsf_10s > 0)) Unsteady_22 = 1.

EXECUTE.

***DSQ-SF IOM (2015) Criteria.***

COMPUTE PEM_22 = 0.

EXECUTE.

IF(SUM(Soreness_22, Minimum_22) > 0) PEM_22 = 1.

EXECUTE.

COMPUTE Neurocog_22 = 0.

EXECUTE.

IF(SUM(Remember_22, Attention_22) > 0) Neurocog_22 = 1.

EXECUTE.

COMPUTE SF_IOM = 0.

EXECUTE.

IF((Fatigue_22 = 1) & (SF_SR = 1) & (PEM_22 = 1) & (Unrefreshed_22 = 1) &

SUM(Neurocog_22, Unsteady_22) >= 1) SF_IOM = 1.

EXECUTE.

VALUE LABELS

SF_IOM

0 ‘Does Not Meet DSQ-SF IOM Criteria’

1 ‘Meets DSQ-SF IOM Criteria’.

EXECUTE.

**DePaul Symptom Questionnaire – Short Form**

**DSQ – SF**

For each symptom below, please circle **one number for frequency** and **one number for severity**:

Please complete the chart from left to right.

| *Frequency:*  Throughout the **past 6 months**,  **how often** have you had this symptom?  For each symptom listed below, circle a number from:  **0 = none of the time**  **1 = a little of the time**  **2 = about half the time**  **3 = most of the time**  **4 = all of the time** | *Severity:*  Throughout the **past 6 months**,  **how much** has this symptom bothered you?  For each symptom listed below, circle a number from:  **0 = symptom not present**  **1 = mild**  **2 = moderate**  **3 = severe**  **4 = very severe** |
| --- | --- |

| **Symptom** | **Frequency:** | **Severity:** |
| --- | --- | --- |
| 1. Fatigue/extreme tiredness | 0 1 2 3 4 | 0 1 2 3 4 |
| 2. Next day soreness or fatigue after non-strenuous, everyday activities | 0 1 2 3 4 | 0 1 2 3 4 |
| 3. Minimum exercise makes you physically tired | 0 1 2 3 4 | 0 1 2 3 4 |
| 4. Feeling unrefreshed after you wake up in the morning | 0 1 2 3 4 | 0 1 2 3 4 |
| 5. Pain or aching in your muscles | 0 1 2 3 4 | 0 1 2 3 4 |
| 6. Bloating | 0 1 2 3 4 | 0 1 2 3 4 |
| 7. Problems remembering things | 0 1 2 3 4 | 0 1 2 3 4 |
| 8. Difficulty paying attention for a long period of time | 0 1 2 3 4 | 0 1 2 3 4 |
| 9. Irritable bowel problems | 0 1 2 3 4 | 0 1 2 3 4 |
| 10. Feeling unsteady on your feet, like you might fall | 0 1 2 3 4 | 0 1 2 3 4 |
| 11. Cold limbs (e.g. arms, legs, hands) | 0 1 2 3 4 | 0 1 2 3 4 |
| 12. Feeling hot or cold for no reason | 0 1 2 3 4 | 0 1 2 3 4 |
| 13. Flu-like symptoms | 0 1 2 3 4 | 0 1 2 3 4 |
| 14. Some smells, foods, medications, or chemicals make you feel sick | 0 1 2 3 4 | 0 1 2 3 4 |

**To Measure Substantial Reduction Requirement in the Case Definitions**

**MOS SURVEY (SF-36)**

**INSTRUCTIONS:**

This survey asks for your views about your health. This information will help keep track of how you feel and

how well you are able to do your usual activities. Answer every question by marking the answer as

indicated. If you are unsure about how to answer a question, please give the best answer you can.

1. In general, would you say your health is: ***(Please circle one)***

Excellent 1

Very good 2

Good 3

Fair 4

Poor 5

2. **Compared to one year ago,** how would you rate your health in general now? ***(Please circle one)***

Much better than one year ago 1

Somewhat better now than one year ago 2

About the same as one year ago 3

Somewhat worse now than one year ago 4

Much worse now than one year ago 5

3. The following items are about activities you might do during a typical day. Does your health now

limit you in these activities? If so, how much?

| **Activities** | **Yes,**  **Limited**  **A Lot** | **Yes,  Limited**  **A Little** | **No, Not**  **Limited**  **At All** |
| --- | --- | --- | --- |
| **Vigorous activities**: running, lifting heavy objects, participating in strenuous sports | 1 | 2 | 3 |
| **Moderate activities**: moving a table, pushing a vacuum cleaner, bowling, playing golf | 1 | 2 | 3 |
| Lifting or carrying groceries | 1 | 2 | 3 |
| Climbing **several** flights of stairs | 1 | 2 | 3 |
| Climbing **one** flight of stairs | 1 | 2 | 3 |
| Bending, kneeling, or stooping | 1 | 2 | 3 |
| Walking **more than a mile** | 1 | 2 | 3 |
| Walking **several blocks** | 1 | 2 | 3 |
| Walking **one** block | 1 | 2 | 3 |
| Bathing or dressing yourself | 1 | 2 | 3 |

4. During the **past 4 weeks**, have you had any of the following problems with your work or other

regular daily activities as a result of your **physical health**?

| **Problems** | **Yes** | **No** |
| --- | --- | --- |
| Cut down on the  **amount of time** you spent on work or other activities | 1 | 2 |
| **Accomplished less** than you would like | 1 | 2 |
| Were limited in the **kind** of work or other activities | 1 | 2 |
| Had **difficulty** performing the work or other activities (For example, it took extra effort) | 1 | 2 |

5. During the **past 4 weeks**, have you had any of the following problems with your work or other

regular daily activities **as a result of any emotional problems** (such as feeling depressed or anxious)?

| **Problems** | **Yes** | **No** |
| --- | --- | --- |
| Cut down the **amount of time** you spent on work or other activities | 1 | 2 |
| **Accomplished less** than you would like | 1 | 2 |
| Didn’t do work or other activities as **carefully** as usual | 1 | 2 |

6. During the **past 4 weeks**, to what extent has your physical health or emotional problems interfered with

your normal social activities with family, neighbors, or groups? ***(Please circle one)***

Not at all 1

Slightly 2

Moderately 3

Quite a bit 4

Extremely 5

7. How much bodily pain have you had during the **past 4 weeks**?

None 1

Very mild 2

Mild 3

Moderate 4

Severe 5

Very Severe 6

8. During the **past 4 weeks**, how much did pain interfere with your normal work (including both work

outside the home and housework)?

Not at all 1

Slightly 2

Moderately 3

Quite a bit 4

Extremely 5

9. These questions are about how you feel and how things have been with you **during the past 4 weeks**.

For each question, please give the one answer that comes closest to the way you have been feeling. How much of the time **during the past 4 weeks**-

| **Questions** | **All**  **of the**  **Time** | **Most**  **of the**  **Time** | **A Good**  **Bit of**  **the Time** | **Some**  **of the**  **Time** | **A Little**  **of the**  **Time** | **None**  **of the**  **Time** |
| --- | --- | --- | --- | --- | --- | --- |
| Did you feel full of pep? | 1 | 2 | 3 | 4 | 5 | 6 |
| Have you been a nervous person? | 1 | 2 | 3 | 4 | 5 | 6 |
| Have you felt so down in the dumps that nothing could cheer you up? | 1 | 2 | 3 | 4 | 5 | 6 |
| Have you felt calm and peaceful? | 1 | 2 | 3 | 4 | 5 | 6 |
| Did you have a lot of energy? | 1 | 2 | 3 | 4 | 5 | 6 |
| Have you felt down-hearted and blue? | 1 | 2 | 3 | 4 | 5 | 6 |
| Did you feel worn out? | 1 | 2 | 3 | 4 | 5 | 6 |
| Have you been a happy person? | 1 | 2 | 3 | 4 | 5 | 6 |
| Did you feel tired? | 1 | 2 | 3 | 4 | 5 | 6 |

10. During the **past 4 weeks**, how much of the time has your physical health or

emotional problems interfered with your social activities (like visiting with friends,

relatives, etc.)?

All of the time 1

Most of the time 2

Some of the time 3

A little of the time 4

None of the time 5

11. How **TRUE** or **FALSE** is each of following statements for you?

| **Statements** | **Definitely**  **True** | **Mostly**  **True** | **Don’t**  **Know** | **Mostly**  **False** | **Definitely**  **False** |
| --- | --- | --- | --- | --- | --- |
| I seem to get sick a little easier than other people | 1 | 2 | 3 | 4 | 5 |
| I am as healthy as anybody I know | 1 | 2 | 3 | 4 | 5 |
| I expect my health to get worse | 1 | 2 | 3 | 4 | 5 |
| My health is excellent | 1 | 2 | 3 | 4 | 5 |
