# Supplementary material for: The Development of the DePaul Symptom Questionnaire: Original, Expanded, Brief, and Pediatric Versions
Source: Front Pediatr. 2018 Nov 6;6:330. doi: 10.3389/fped.2018.00330 (PMC6232226; doi:10.3389/fped.2018.00330)
Supplement: Supplementary file 4 [file Data_Sheet_4.docx]

**Data Sheet 4.**

**DSQ Pediatric (DSQ-Ped)**

**Parent Form**

This document contains the following material:

1. Case definition scoring rules and associated symptoms for the following criteria:
   1. Jason et al. (2006) Pediatric Case Definition for ME and CFS
   2. Rowe et al. (2017) Pediatric ME/CFS Case Definition
2. Syntax for the following case definitions:
   1. Jason et al. (2006) Pediatric Case Definition for ME and CFS
   2. Rowe et al. (2017) Pediatric ME/CFS Case Definition
3. Hard copy of the DSQ-Ped (Parent Form)

The DSQ-Ped (Parent Form) can be downloaded from the REDCap shared library. You can view the instrument here: <https://redcap.is.depaul.edu/surveys/?s=3FPRX49778>

**DSQ-Ped**

**(Parent Form)**

**Case Definition Criteria:**

*Note: Results from both the parent and child forms of the DSQ-Ped should be considered when making diagnostic decisions.*

**Pediatric Case Definition for ME and CFS (Jason et al., 2006)**

- 3+ months Fatigue (Question 15; frequency and severity scores >= 2)
  - *Note: Due to challenges in measuring the ‘substantial reduction in functioning’ criterion among children, clinician assessment is required to determine if the child has demonstrated a significant reduction in educational, social, and/or personal activities*
- At least 1 symptom from each of the following domains (frequency and severity scores >= 2):
  - Post-exertional malaise (Questions 16-20)
  - Unrefreshing sleep (Questions 21-25)
  - Pain (Question 26-31; 42-45)
- At least 2 neurocognitive manifestations (Questions 32-41; frequency and severity scores >= 2)
- At least 1 symptom from 2 of the following categories (frequency and severity scores >= 2):
  - Autonomic (Questions 46-49)
  - Neuroendocrine (Questions 50-57)
  - Immune (Questions 58-61)

**Pediatric ME/CFS Criteria (Rowe et al., 2006)**

- 6+ months of illness (Question 68a >= 6)
- Impaired function; at least 1 of the following symptoms:
  - Question 75 (*“Yes”*)
  - Question 80 (“*Yes*”)
- Fatigue
  - Question 15 (frequency and severity scores >= 2)
  - Not relieved by rest: Question 69 (“*Some of it goes away*” or “*None of it goes away*”)
- Post-exertional symptoms (At least 1 symptom from Questions 16-20; frequency and severity scores >= 2)
- At least 1 symptom from 2 of the following categories (frequency and severity scores >= 2):
  - Sleep problems (Questions 21-25)
  - Cognitive problems (Questions 33-41)
  - Pain (Questions 26-27; 30-31; 58-59)

**DSQ-Ped (Parent Form)**

**SPSS Case Definition Syntax:**

**Results from both the parent and child forms of the DSQ-Ped should be considered when making diagnostic decisions*.*

**Scoring Note: To determine which variables are associated with which items, note that variable names utilize the items numbers present in the questionnaire*.*

********************************************************************************

*DSQ-Ped (Parent Form)*.

* Pediatric Case Definition for ME and CFS (Jason et al., 2006)*.

********************************************************************************.

*Fatigue*.

**Note: Due to challenges in measuring the ‘substantial reduction in functioning’ criterion among children, clinician assessment is required to determine if the child has demonstrated a significant reduction in educational, social, and/or personal activities*.*

COMPUTE PedP_Fatigue = 0.

EXECUTE.

IF((dsqp_p_15f >= 2) & (dsqp_p_15s >= 2)) PedP_Fatigue = 1.

EXECUTE.

*Post-exertional malaise*.

COMPUTE PedP_16 = 0.

EXECUTE.

IF((dsqp_p_16f >= 2) & (dsqp_p_16s >= 2)) PedP_16 = 1.

EXECUTE.

COMPUTE PedP_17 = 0.

EXECUTE.

IF((dsqp_p_17f >= 2) & (dsqp_p_17s >= 2)) PedP_17 = 1.

EXECUTE.

COMPUTE PedP_18 = 0.

EXECUTE.

IF((dsqp_p_18f >= 2) & (dsqp_p_18s >= 2)) PedP_18 = 1.

EXECUTE.

COMPUTE PedP_19 = 0.

EXECUTE.

IF((dsqp_p_19f >= 2) & (dsqp_p_19s >= 2)) PedP_19 = 1.

EXECUTE.

COMPUTE PedP_20 = 0.

EXECUTE.

IF((dsqp_p_20f >= 2) & (dsqp_p_20s >= 2)) PedP_20 = 1.

EXECUTE.

COMPUTE PedP_PEM = 0.

EXECUTE.

IF(SUM(PedP_16, PedP_17, PedP_18, PedP_19, PedP_20) >= 1) PedP_PEM = 1.

EXECUTE.

*Unrefreshing Sleep*.

COMPUTE PedP_21 = 0.

EXECUTE.

IF((dsqp_p_21f >= 2) & (dsqp_p_21s >= 2)) PedP_21 = 1.

EXECUTE.

COMPUTE PedP_22 = 0.

EXECUTE.

IF((dsqp_p_22f >= 2) & (dsqp_p_22s >= 2)) PedP_22 = 1.

EXECUTE.

COMPUTE PedP_23 = 0.

EXECUTE.

IF((dsqp_p_23f >= 2) & (dsqp_p_23s >= 2)) PedP_23 = 1.

EXECUTE.

COMPUTE PedP_24 = 0.

EXECUTE.

IF((dsqp_p_24f >= 2) & (dsqp_p_24s >= 2)) PedP_24 = 1.

EXECUTE.

COMPUTE PedP_25 = 0.

EXECUTE.

IF((dsqp_p_25f >= 2) & (dsqp_p_25s >= 2)) PedP_25 = 1.

EXECUTE.

COMPUTE PedP_Sleep = 0.

EXECUTE.

IF(SUM(PedP_21, PedP_22, PedP_23, PedP_24, PedP_25) >= 1) PedP_Sleep = 1.

EXECUTE.

*Pain*.

COMPUTE PedP_26 = 0.

EXECUTE.

IF((dsqp_p_26f >= 2) & (dsqp_p_26s >= 2)) PedP_26 = 1.

EXECUTE.

COMPUTE PedP_27 = 0.

EXECUTE.

IF((dsqp_p_27f >= 2) & (dsqp_p_27s >= 2)) PedP_27 = 1.

EXECUTE.

COMPUTE PedP_28 = 0.

EXECUTE.

IF((dsqp_p_28f >= 2) & (dsqp_p_28s >= 2)) PedP_28 = 1.

EXECUTE.

COMPUTE PedP_29 = 0.

EXECUTE.

IF((dsqp_p_29f >= 2) & (dsqp_p_29s >= 2)) PedP_29 = 1.

EXECUTE.

COMPUTE PedP_30 = 0.

EXECUTE.

IF((dsqp_p_30f >= 2) & (dsqp_p_30s >= 2)) PedP_30 = 1.

EXECUTE.

COMPUTE PedP_31 = 0.

EXECUTE.

IF((dsqp_p_31f >= 2) & (dsqp_p_31s >= 2)) PedP_31 = 1.

EXECUTE.

COMPUTE PedP_42 = 0.

EXECUTE.

IF((dsqp_p_42f >= 2) & (dsqp_p_42s >= 2)) PedP_42 = 1.

EXECUTE.

COMPUTE PedP_43 = 0.

EXECUTE.

IF((dsqp_p_43f >= 2) & (dsqp_p_43s >= 2)) PedP_43 = 1.

EXECUTE.

COMPUTE PedP_44 = 0.

EXECUTE.

IF((dsqp_p_44f >= 2) & (dsqp_p_44s >= 2)) PedP_44 = 1.

EXECUTE.

COMPUTE PedP_45 = 0.

EXECUTE.

IF((dsqp_p_45f >= 2) & (dsqp_p_45s >= 2)) PedP_45 = 1.

EXECUTE.

COMPUTE PedP_Pain = 0.

IF(SUM(PedP_26, PedP_27, PedP_28, PedP_29, PedP_30, PedP_31, PedP_42, PedP_43, PedP_44, PedP_45) >= 1) PedP_Pain = 1.

EXECUTE.

*Neurocognitive*.

COMPUTE PedP_32 = 0.

EXECUTE.

IF((dsqp_p_32f >= 2) & (dsqp_p_32s >= 2)) PedP_32 = 1.

EXECUTE.

COMPUTE PedP_33 = 0.

EXECUTE.

IF((dsqp_p_33f >= 2) & (dsqp_p_33s >= 2)) PedP_33 = 1.

EXECUTE.

COMPUTE PedP_34 = 0.

EXECUTE.

IF((dsqp_p_34f >= 2) & (dsqp_p_34s >= 2)) PedP_34 = 1.

EXECUTE.

COMPUTE PedP_35 = 0.

EXECUTE.

IF((dsqp_p_35f >= 2) & (dsqp_p_35s >= 2)) PedP_35 = 1.

EXECUTE.

COMPUTE PedP_36 = 0.

EXECUTE.

IF((dsqp_p_36f >= 2) & (dsqp_p_36s >= 2)) PedP_36 = 1.

EXECUTE.

COMPUTE PedP_37 = 0.

EXECUTE.

IF((dsqp_p_37f >= 2) & (dsqp_p_37s >= 2)) PedP_37 = 1.

EXECUTE.

COMPUTE PedP_38 = 0.

EXECUTE.

IF((dsqp_p_38f >= 2) & (dsqp_p_38s >= 2)) PedP_38 = 1.

EXECUTE.

COMPUTE PedP_39 = 0.

EXECUTE.

IF((dsqp_p_39f >= 2) & (dsqp_p_39s >= 2)) PedP_39 = 1.

EXECUTE.

COMPUTE PedP_40 = 0.

EXECUTE.

IF((dsqp_p_40f >= 2) & (dsqp_p_40s >= 2)) PedP_40 = 1.

EXECUTE.

COMPUTE PedP_41 = 0.

EXECUTE.

IF((dsqp_p_41f >= 2) & (dsqp_p_41s >= 2)) PedP_41 = 1.

EXECUTE.

COMPUTE PedP_Cognitive = 0.

EXECUTE.

IF(SUM(PedP_32, PedP_33, PedP_34, PedP_35, PedP_36, PedP_37, PedP_38, PedP_39, PedP_40, PedP_41) >=2) PedP_Cognitive = 1.

EXECUTE.

*Autonomic*.

COMPUTE PedP_46 = 0.

EXECUTE.

IF((dsqp_p_46f >= 2) & (dsqp_p_46s >= 2)) PedP_46 = 1.

EXECUTE.

COMPUTE PedP_47 = 0.

EXECUTE.

IF((dsqp_p_47f >= 2) & (dsqp_p_47s >= 2)) PedP_47 = 1.

EXECUTE.

COMPUTE PedP_48 = 0.

EXECUTE.

IF((dsqp_p_48f >= 2) & (dsqp_p_48s >= 2)) PedP_48 = 1.

EXECUTE.

COMPUTE PedP_49 = 0.

EXECUTE.

IF((dsqp_p_49f >= 2) & (dsqp_p_49s >= 2)) PedP_49 = 1.

EXECUTE.

COMPUTE PedP_Autonomic = 0.

EXECUTE.

IF(SUM(PedP_46, PedP_47, PedP_48, PedP_49) >= 1) PedP_Autonomic = 1.

EXECUTE.

*Neuroendocrine*.

COMPUTE PedP_50 = 0.

EXECUTE.

IF((dsqp_p_50f >= 2) & (dsqp_p_50s >= 2)) PedP_50 = 1.

EXECUTE.

COMPUTE PedP_51 = 0.

EXECUTE.

IF((dsqp_p_51f >= 2) & (dsqp_p_51s >= 2)) PedP_51 = 1.

EXECUTE.

COMPUTE PedP_52 = 0.

EXECUTE.

IF((dsqp_p_52f >= 2) & (dsqp_p_52s >= 2)) PedP_52 = 1.

EXECUTE.

COMPUTE PedP_53 = 0.

EXECUTE.

IF((dsqp_p_53f >= 2) & (dsqp_p_53s >= 2)) PedP_53 = 1.

EXECUTE.

COMPUTE PedP_54 = 0.

EXECUTE.

IF((dsqp_p_54f >= 2) & (dsqp_p_54s >= 2)) PedP_54 = 1.

EXECUTE.

COMPUTE PedP_55 = 0.

EXECUTE.

IF((dsqp_p_55f >= 2) & (dsqp_p_55s >= 2)) PedP_55 = 1.

EXECUTE.

COMPUTE PedP_56 = 0.

EXECUTE.

IF((dsqp_p_56f >= 2) & (dsqp_p_56s >= 2)) PedP_56 = 1.

EXECUTE.

COMPUTE PedP_57 = 0.

EXECUTE.

IF((dsqp_p_57f >= 2) & (dsqp_p_57s >= 2)) PedP_57 = 1.

EXECUTE.

COMPUTE PedP_Neuroendo = 0.

EXECUTE.

IF(SUM(PedP_50, PedP_51, PedP_52, PedP_53, PedP_54, PedP_55, PedP_56, PedP_57) >= 1) PedP_Neuroendo = 1.

EXECUTE.

*Immune*

COMPUTE PedP_58 = 0.

EXECUTE.

IF((dsqp_p_58f >= 2) & (dsqp_p_58s >= 2)) PedP_58 = 1.

EXECUTE.

COMPUTE PedP_59 = 0.

EXECUTE.

IF((dsqp_p_59f >= 2) & (dsqp_p_59s >= 2)) PedP_59 = 1.

EXECUTE.

COMPUTE PedP_60 = 0.

EXECUTE.

IF((dsqp_p_60f >= 2) & (dsqp_p_60s >= 2)) PedP_60 = 1.

EXECUTE.

COMPUTE PedP_61 = 0.

EXECUTE.

IF((dsqp_p_61f >= 2) & (dsqp_p_61s >= 2)) PedP_61 = 1.

EXECUTE.

COMPUTE PedP_Immune = 0.

EXECUTE.

IF(SUM(PedP_58, PedP_59, PedP_60, PedP_61) >= 1) PedP_Immune = 1.

EXECUTE.

***Jason et al. (2006) Pediatric Case Definition for ME and CFS***.

COMPUTE PedP_ANI2 = 0.

EXECUTE.

IF(SUM(PedP_Autonomic, PedP_Neuroendo, PedP_Immune) >= 2) PedP_ANI2 = 1.

EXECUTE.

COMPUTE PedP_Jason = 0.

EXECUTE.

IF(SUM(PedP_Fatigue, PedP_PEM, PedP_Sleep, PedP_Pain, PedP_Cognitive, PedP_ANI2) = 6) PedP_Jason = 1.

EXECUTE.

VALUE LABELS

PedP_Jason

0 ‘Does not meet Jason et al Pediatric ME and CFS Criteria’

1 ‘Meets Jason et al Pediatric ME and CFS Criteria’.

EXECUTE.

********************************************************************************.

*DSQ-Ped (Parent Form)*.

* Pediatric ME/CFS Criteria (Rowe et al., 2017)*.

********************************************************************************.

*Six months of illness*.

COMPUTE RoweP_Six = 0.

EXECUTE.

IF(dsqp_p_68a >= 6) RoweP_Six = 1.

EXECUTE.

*Impaired Function*.

COMPUTE RoweP_75 = 0.

EXECUTE.

IF(dsqp_p_75 = 1) RoweP_75 = 1.

EXECUTE.

COMPUTE RoweP_80 = 0.

EXECUTE.

IF(dsqp_p_80 = 1) RoweP_80 = 1.

EXECUTE.

COMPUTE RoweP_Function = 0.

EXECUTE.

IF((RoweP_75 = 1) | (RoweP_80 = 1)) RoweP_Function = 1.

EXECUTE.

*Fatigue*.

COMPUTE RoweP_15 = 0.

EXECUTE.

IF((dsqp_p_15f >= 2) & (dsqp_p_15s >= 2)) RoweP_15 = 1.

EXECUTE.

COMPUTE RoweP_69 = 0.

EXECUTE.

IF((dsqp_p_69 = 2) | (dsqp_p_69 = 3)) RoweP_69 = 1.

EXECUTE.

COMPUTE RoweP_Fatigue = 0.

EXECUTE.

IF(SUM(RoweP_15, RoweP_69) = 2) RoweP_Fatigue = 1.

EXECUTE.

*Post-exertional malaise*.

COMPUTE RoweP_16 = 0.

EXECUTE.

IF((dsqp_p_16f >= 2) & (dsqp_p_16s >= 2)) RoweP_16 = 1.

EXECUTE.

COMPUTE RoweP_17 = 0.

EXECUTE.

IF((dsqp_p_17f >= 2) & (dsqp_p_17s >= 2)) RoweP_17 = 1.

EXECUTE.

COMPUTE RoweP_18 = 0.

EXECUTE.

IF((dsqp_p_18f >= 2) & (dsqp_p_18s >= 2)) RoweP_18 = 1.

EXECUTE.

COMPUTE RoweP_19 = 0.

EXECUTE.

IF((dsqp_p_19f >= 2) & (dsqp_p_19s >= 2)) RoweP_19 = 1.

EXECUTE.

COMPUTE RoweP_20 = 0.

EXECUTE.

IF((dsqp_p_20f >= 2) & (dsqp_p_20s >= 2)) RoweP_20 = 1.

EXECUTE.

COMPUTE RoweP_PEM = 0.

EXECUTE.

IF(SUM(RoweP_16, RoweP_17, RoweP_18, RoweP_19, RoweP_20) >= 1) RoweP_PEM = 1.

EXECUTE.

*Sleep Problems*.

COMPUTE RoweP_21 = 0.

EXECUTE.

IF((dsqp_p_21f >= 2) & (dsqp_p_21s >= 2)) RoweP_21 = 1.

EXECUTE.

COMPUTE RoweP_22 = 0.

EXECUTE.

IF((dsqp_p_22f >= 2) & (dsqp_p_22s >= 2)) RoweP_22 = 1.

EXECUTE.

COMPUTE RoweP_23 = 0.

EXECUTE.

IF((dsqp_p_23f >= 2) & (dsqp_p_23s >= 2)) RoweP_23 = 1.

EXECUTE.

COMPUTE RoweP_24 = 0.

EXECUTE.

IF((dsqp_p_24f >= 2) & (dsqp_p_24s >= 2)) RoweP_24 = 1.

EXECUTE.

COMPUTE RoweP_25 = 0.

EXECUTE.

IF((dsqp_p_25f >= 2) & (dsqp_p_25s >= 2)) RoweP_25 = 1.

EXECUTE.

COMPUTE RoweP_Sleep = 0.

EXECUTE.

IF(SUM(RoweP_21, RoweP_22, RoweP_23, RoweP_24, RoweP_25) >= 1) RoweP_Sleep = 1.

EXECUTE.

*Cognitive Problems*.

COMPUTE RoweP_33 = 0.

EXECUTE.

IF((dsqp_p_33f >= 2) & (dsqp_p_33s >= 2)) RoweP_33 = 1.

EXECUTE.

COMPUTE RoweP_34 = 0.

EXECUTE.

IF((dsqp_p_34f >= 2) & (dsqp_p_34s >= 2)) RoweP_34 = 1.

EXECUTE.

COMPUTE RoweP_35 = 0.

EXECUTE.

IF((dsqp_p_35f >= 2) & (dsqp_p_35s >= 2)) RoweP_35 = 1.

EXECUTE.

COMPUTE RoweP_36 = 0.

EXECUTE.

IF((dsqp_p_36f >= 2) & (dsqp_p_36s >= 2)) RoweP_36 = 1.

EXECUTE.

COMPUTE RoweP_37 = 0.

EXECUTE.

IF((dsqp_p_37f >= 2) & (dsqp_p_37s >= 2)) RoweP_37 = 1.

EXECUTE.

COMPUTE RoweP_38 = 0.

EXECUTE.

IF((dsqp_p_38f >= 2) & (dsqp_p_38s >= 2)) RoweP_38 = 1.

EXECUTE.

COMPUTE RoweP_39 = 0.

EXECUTE.

IF((dsqp_p_39f >= 2) & (dsqp_p_39s >= 2)) RoweP_39 = 1.

EXECUTE.

COMPUTE RoweP_40 = 0.

EXECUTE.

IF((dsqp_p_40f >= 2) & (dsqp_p_40s >= 2)) RoweP_40 = 1.

EXECUTE.

COMPUTE RoweP_41 = 0.

EXECUTE.

IF((dsqp_p_41f >= 2) & (dsqp_p_41s >= 2)) RoweP_41 = 1.

EXECUTE.

COMPUTE RoweP_Cognitive = 0.

EXECUTE.

IF(SUM(RoweP_33, RoweP_34, RoweP_35, RoweP_36, RoweP_37, RoweP_38, RoweP_39, RoweP_40, RoweP_41) >= 1) RoweP_Cognitive = 1.

EXECUTE.

*Pain*.

COMPUTE RoweP_26 = 0.

EXECUTE.

IF((dsqp_p_26f >= 2) & (dsqp_p_26s >= 2)) RoweP_26 = 1.

EXECUTE.

COMPUTE RoweP_27 = 0.

EXECUTE.

IF((dsqp_p_27f >= 2) & (dsqp_p_27s >= 2)) RoweP_27 = 1.

EXECUTE.

COMPUTE RoweP_30 = 0.

EXECUTE.

IF((dsqp_p_30f >= 2) & (dsqp_p_30s >= 2)) RoweP_30 = 1.

EXECUTE.

COMPUTE RoweP_31 = 0.

EXECUTE.

IF((dsqp_p_31f >= 2) & (dsqp_p_31s >= 2)) RoweP_31 = 1.

EXECUTE.

COMPUTE RoweP_58 = 0.

EXECUTE.

IF((dsqp_p_58f >= 2) & (dsqp_p_58s >= 2)) RoweP_58 = 1.

EXECUTE.

COMPUTE RoweP_59 = 0.

EXECUTE.

IF((dsqp_p_59f >= 2) & (dsqp_p_59s >= 2)) RoweP_59 = 1.

EXECUTE.

COMPUTE RoweP_Pain = 0.

EXECUTE.

IF(SUM(RoweP_26, RoweP_27, RoweP_30, RoweP_31, RoweP_58, RoweP_59) >= 1) RoweP_Pain = 1.

EXECUTE.

***Pediatric ME/CFS Criteria (Rowe et al., 2017)***.

COMPUTE RoweP_SCP2 = 0.

EXECUTE.

IF(SUM(RoweP_Sleep, RoweP_Cognitive, RoweP_Pain) >= 2) RoweP_SCP2 = 1.

EXECUTE.

COMPUTE Rowe_Parent = 0.

EXECUTE.

IF(SUM(RoweP_Six, RoweP_Function, RoweP_Fatigue, RoweP_PEM, RoweP_SCP2) = 5) Rowe_Parent = 1.

EXECUTE.

VALUE LABELS

Rowe_Parent

0 ‘ Does Not Meet Rowe et al Pediatric MECFS Criteria’

1 ‘Meets Rowe et al Pediatric MECFS Criteria’.

EXECUTE.

**DePaul Symptom Questionnaire – Pediatric (Parent Report Form)**

**DSQ-Ped (Parent)**

Participant ID: _______________

1. Today’s Date: _______________
2. Child’s Date of Birth: _______________
3. Child’s Age: _______________
4. Child’s Gender:
   - Male
   - Female
   - Other
   - Prefer not to respond
5. Child’s Grade in School:
   - Preschool
   - Kindergarten
   - 1^st^ Grade
   - 2^nd^ Grade
   - 3^rd^ Grade
   - 4^th^ Grade
   - 5^th^ Grade
   - 6^th^ Grade
   - 7^th^ Grade
   - 8^th^ Grade
   - 9^th^ Grade (Freshman)
   - 10^th^ Grade (Sophomore)
   - 11^th^ Grade (Junior)
   - 12^th^ Grade (Senior)
   - Ungraded
     1. If ungraded, how many years has your child attended school? _______________
6. Do you have any other children?
   - Yes
   - No
     1. If so, how many additional children do you have? _______________
     2. How many of your children are under 18 years old? _______________
7. How many people live in your home (including you)? _______________
8. What is the highest degree or level of education you have completed?
   - Less than high school
   - Some high school
   - High school or GED
   - Partial college (at least one year) or specialized training
   - Standard college degree
   - Graduate or professional degree, including Master’s and Doctorate
9. What category best describes your annual household income?
   - Less than $24,999
   - $25,000 - $49,999
   - $50,000 - $99,999
   - $100,000 - $149,999
   - $150,000 - $199,999
   - $200,000 - $249,999
   - $250,000 or more
   - Prefer not to respond
10. How would you describe your current employment status?
    - Employed full-time
    - Employed part-time
    - Unemployed / Looking for work
    - Student
    - Homemaker
    - Retired
11. What is your current marital status?
    - Married / Living with partner
    - Separated
    - Widowed
    - Divorced
    - Never married
12. What is your race / ethnicity (check all that apply)?

- American Indian or Alaskan Native
- Asian or Pacific Islander
- Black / African American
- White / Caucasian
- Prefer not to respond
- Other (please specify)

1. If other, please specify: _______________
2. Are you of Latino or Hispanic origin?
   - Yes
   - No
3. What is your religious affiliation?
   - Atheist or Agnostic
   - Buddhist
   - Christian
   - Hindu
   - Jewish
   - Muslim
   - Prefer not to respond
   - Other (please specify)
4. If other, please specify: _______________

For the next questions, think about your child over the **past 3 months**. Circle how often your child has had each symptom (frequency) and how much each symptom has bothered him/her (severity).

Please circle **one number for frequency** and **one number for severity**, using the following scales:

| *Frequency:*  Throughout the **past 3 months**,  **how often** has your child had this symptom?  For each symptom listed below, circle a number from:  **0 = none of the time**  **1 = a little of the time**  **2 = about half the time**  **3 = most of the time**  **4 = all of the time** | *Severity:*  Throughout the **past 3 months**,  **how much** has this symptom bothered your child?  For each symptom listed below, circle a number from:  **0 = no problem**  **1 = mild**  **2 = moderate**  **3 = severe**  **4 = very severe** |
| --- | --- |

| **Symptom** | **Frequency:** | **Severity:** |
| --- | --- | --- |
| 15. Fatigue / Extreme tiredness | 0 1 2 3 4 | 0 1 2 3 4 |
| 16. Body feels heavy after starting to exercise | 0 1 2 3 4 | 0 1 2 3 4 |
| 17. Feeling sore / very tired after everyday activities, like walking around the house | 0 1 2 3 4 | 0 1 2 3 4 |
| 18. Mind is tired after just a little effort | 0 1 2 3 4 | 0 1 2 3 4 |
| 19. A little bit of exercise makes your child’s body tired | 0 1 2 3 4 | 0 1 2 3 4 |
| 20. Child’s body is tired or he/she feels sick after a little bit of activity | 0 1 2 3 4 | 0 1 2 3 4 |
| 21. Feeling tired after he/she wakes up in the morning | 0 1 2 3 4 | 0 1 2 3 4 |
| 22. Needing to nap daily | 0 1 2 3 4 | 0 1 2 3 4 |
| 23. Problems falling asleep | 0 1 2 3 4 | 0 1 2 3 4 |
| 24. Problems staying asleep | 0 1 2 3 4 | 0 1 2 3 4 |
| 25. Waking up early in the morning (like 3:00 am) | 0 1 2 3 4 | 0 1 2 3 4 |
| 26. Pain or aching in muscles | 0 1 2 3 4 | 0 1 2 3 4 |
| 27. Pain / Stiffness / Tenderness in more than one joint, without swelling or redness | 0 1 2 3 4 | 0 1 2 3 4 |
| 28. Eye pain | 0 1 2 3 4 | 0 1 2 3 4 |
| 29. Chest pain / Heartburn | 0 1 2 3 4 | 0 1 2 3 4 |
| 30. Abdomen / Stomach pain | 0 1 2 3 4 | 0 1 2 3 4 |
| 31. Headaches | 0 1 2 3 4 | 0 1 2 3 4 |
| 32. Muscle twitches | 0 1 2 3 4 | 0 1 2 3 4 |
| **Symptom** | **Frequency:** | **Severity:** |
| 33. Problems remembering things | 0 1 2 3 4 | 0 1 2 3 4 |
| 34. Difficulty paying attention for a long period of time | 0 1 2 3 4 | 0 1 2 3 4 |
| 35. Difficulty finding the right word to say | 0 1 2 3 4 | 0 1 2 3 4 |
| 36. Difficulty understanding things | 0 1 2 3 4 | 0 1 2 3 4 |
| 37. Only able to focus on one thing at a time | 0 1 2 3 4 | 0 1 2 3 4 |
| 38. Slowness of thought | 0 1 2 3 4 | 0 1 2 3 4 |
| 39. Absent-mindedness or forgetfulness | 0 1 2 3 4 | 0 1 2 3 4 |
| 40. Frequently losing train of thought | 0 1 2 3 4 | 0 1 2 3 4 |
| 41. Trouble with math or numbers | 0 1 2 3 4 | 0 1 2 3 4 |
| 42. Nausea | 0 1 2 3 4 | 0 1 2 3 4 |
| 43. Upset stomach | 0 1 2 3 4 | 0 1 2 3 4 |
| 44. Vomiting | 0 1 2 3 4 | 0 1 2 3 4 |
| 45. Ringing in ears | 0 1 2 3 4 | 0 1 2 3 4 |
| 46. Feeling unsteady on feet, like he/she might fall | 0 1 2 3 4 | 0 1 2 3 4 |
| 47. Shortness of breath or trouble catching his/her breath | 0 1 2 3 4 | 0 1 2 3 4 |
| 48. Dizziness | 0 1 2 3 4 | 0 1 2 3 4 |
| 49. Irregular heart beats | 0 1 2 3 4 | 0 1 2 3 4 |
| 50. Losing or gaining weight without trying | 0 1 2 3 4 | 0 1 2 3 4 |
| 51. Sweating hands | 0 1 2 3 4 | 0 1 2 3 4 |
| 52. Not wanting to eat | 0 1 2 3 4 | 0 1 2 3 4 |
| 53. Night sweats | 0 1 2 3 4 | 0 1 2 3 4 |
| 54. Feeling chills or shivers | 0 1 2 3 4 | 0 1 2 3 4 |
| 55. Feeling hot or cold for no reason | 0 1 2 3 4 | 0 1 2 3 4 |
| 56. Feeling like he/she has a high temperature | 0 1 2 3 4 | 0 1 2 3 4 |
| 57. Feeling like he/she has a low temperature | 0 1 2 3 4 | 0 1 2 3 4 |
| 58. Sore throat | 0 1 2 3 4 | 0 1 2 3 4 |
| 59. Tender / sore lymph nodes | 0 1 2 3 4 | 0 1 2 3 4 |
| 60. Fever and sweats | 0 1 2 3 4 | 0 1 2 3 4 |
| 61. Some smells, foods, or chemicals make your child feel sick | 0 1 2 3 4 | 0 1 2 3 4 |
| 62. Rashes | 0 1 2 3 4 | 0 1 2 3 4 |
| 63. Allergies | 0 1 2 3 4 | 0 1 2 3 4 |

1. When your child **first became sick**, what were his/her worst three symptoms? Please select from the list above, or if your child is not sick, write “my child is not ill.”
   1. Worst symptom: _________________________
   2. Second-worst symptom: _________________________
   3. Third-worst symptom: _________________________
2. **Right now**, what are his/her worst three symptoms? Please select from the list above, or if your child is not sick, write “my child is not ill.”
   1. Worst symptom: _________________________
   2. Second-worst symptom: _________________________
   3. Third-worst symptom: _________________________
3. How long did it take for your child’s problem with fatigue or tiredness to get started?
   - Rapidly - within 24 hours
   - Over 1 week
   - Over 1 month
   - Over 2-3 months
   - Over 4-6 months
   - Over 7-11 months
   - Over 1-2 years
   - Longer than 2 years
   - My child has always experienced fatigue
   - My child does not have fatigue
4. If your child has headaches now, does he/she get them more often, in a different place, or do the headaches feel worse than they did in the past?
   - Headaches happen more often
   - Headaches feel worse / more severe
   - Headaches are in a different place / spot
   - My child doesn’t have headaches
5. Has your child been experiencing problems with fatigue / extreme tiredness for **at least one month**?
   - Yes
   - No
6. If yes, for about how many months? _______________
7. If your child rests, does all of his/her fatigue go away, some of it go away, or none of it go away?
   - All of it goes away
   - Some of it goes away
   - None of it goes away
   - My child does not have fatigue
   1. If all or some of it goes away, for **how many hours** does your child have to rest before his/her fatigue gets better? _______________
8. Does your child participate in any hobbies or activities outside of school?
   - Yes
   - No
   1. If no, why? Select all that apply.
      - Not interested
      - No time
      - Would like to, but cannot because of problems with fatigue / energy
      - Cannot because hobbies or activities make symptoms worse
9. Does your child limit or cut back activity levels to avoid feeling even more tired?
   - Yes
   - No
10. How would you describe the way your child’s fatigue problem is changing over time?
    - My child’s fatigue is getting worse
    - My child has good and bad periods
    - There is no change
    - My child’s fatigue is getting better
    - My child does not have fatigue
11. Do your child’s symptoms change over time?
    - Yes
    - No
12. Did your child’s fatigue or energy problems start after he/she experienced any of the following? Check all that apply. If you check an option, please provide more details in the appropriate blank (e.g., year it began, treatment history, etc.)
    - - a. An infectious illness ________________________________________________________________________________________________________________________________________________
    - b. An accident ________________________________________________________________________________________________________________________________________________
    - c. A trip or vacation ________________________________________________________________________________________________________________________________________________
    - d. An immunization (shot at doctor’s office) ________________________________________________________________________________________________________________________________________________
    - e. Surgery ________________________________________________________________________________________________________________________________________________
    - f. Severe stress (bad or unhappy events) ________________________________________________________________________________________________________________________________________________
    - g. Other (please describe below) ________________________________________________________________________________________________________________________________________________
13. Since the start of your child’s problems with fatigue or energy, have your child’s symptoms caused him/her to reduce the time he/she does activities by 50% or more?
    - Yes
    - No
    - Not having a problem with fatigue / energy
14. Does your child seem to catch illnesses more easily than other people his/her age?
    1. Yes
    2. No
15. Does it seem to take your child longer to get better after he/she is sick than other people his/her age?
    1. Yes
    2. No
16. Is your child more uncomfortable than other people his/her age when it is extremely hot or cold?
    1. Yes
    2. No
17. Thinking about the past month, about how many **hours per week** in a typical week has your child spent on:
    1. School / School activities _______________
    2. Sports / Recreational activities _______________
    3. Activities with friends and family _______________
    4. Work activities _______________
18. In the past 4 weeks, has your child had to reduce the number of hours he/she previously spent (prior to illness) on school, sports or recreational activities, activities with friends or family, or work because of health or problems with fatigue / energy?
    - Yes
    - No
    - Not having problems with fatigue / energy

If you answered “Yes” to the previous question, **before** your child’s fatigue/energy-related problems, approximately how many hours per week did he/she used to spend on:

- 1. School / School activities _______________
  2. Sports / Recreational activities _______________
  3. Activities with friends and family _______________
  4. Work activities _______________

1. In a typical week, please rate the amount of energy your child has available using a scale from 1 to 100, where 1 = no energy and 100 = a lot of energy: _______________
2. In a typical week, please rate the amount of energy your child uses on a scale from 1 to 100, where 1 = no energy and 100 = a lot of energy: _______________
3. In a typical week, please rate the amount of **fatigue** your child has using a scale from 1 to 100, where 1 = no fatigue and 100 = very bad fatigue: _______________
4. Does your child have any medical illnesses that might be causing his/her symptoms?
   - Yes
   - No
   1. If yes, please name the illnesses and the year they began:

________________________________________________________________________________________________________________________________________________________________________________________________________________________________________________________________________________________________

- 1. For which of these medical illnesses is your child currently receiving treatment? Please list the illness(es) and associated treatment(s):

________________________________________________________________________________________________________________________________________________________________________________________________________________________________________________________________________________________________

1. Is your child currently taking any medications (over the counter or prescription)?
   - Yes
   - No
   1. If yes, what medications? ________________________________________________________________________________________________________________________________________________
2. Do you think any medications are causing your child’s problem with fatigue / energy?
   - Yes
   - No
   1. If yes, which medications? ________________________________________________________________________________________________________________________________________________________________________________________________________________________________________________________________________________________________
3. Do you think anything in your child’s personal life or environment is causing your child’s problem with fatigue/energy?
   - Yes
   - No
   - Not having problems with fatigue / energy
   1. If yes, please describe: ________________________________________________________________________________________________________________________________________________________________________________________________________________________________________________________________________________________________
4. Has your child been diagnosed with Chronic Fatigue Syndrome or Myalgic Encephalomyelitis?
   - Yes
   - No
   1. If yes, in what year was he/she diagnosed? _______________
5. Does your child currently have a diagnosis of Chronic Fatigue Syndrome or Myalgic Encephalomyelitis?
   - Yes
   - No
   1. If your child has ever been diagnosed with Chronic Fatigue Syndrome or Myalgic Encephalomyelitis, who diagnosed him/her? Select all that apply.
      - Medical Doctor
      - Alternative Practitioner
      - Self-diagnosed
6. Have any of your child’s family members been diagnosed with Chronic Fatigue Syndrome or Myalgic Encephalomyelitis?
   - Yes
   - No
   1. If Yes, please list their relation to your child and current age:

________________________________________________________________________________________________________________________________________________________________________________________________________________________________________________________________________________________________

1. Did your child experience any of the following symptoms regularly and repeatedly in the months and years before his/her fatigue / energy problems began? Select all that apply.
   - Sore throat
   - Tender / Sore lymph nodes
   - Unrefreshing sleep
   - Impaired memory and concentration
   - Prolonged fatigue following physical or mental exertion
   - Muscle pain
   - Headaches
   - Joint pain
   - Not having a problem with fatigue / energy
2. Have you ever consulted a medical doctor or health professional about your child’s fatigue / energy problem?
   - Yes
   - No
3. Do you currently have a medical doctor overseeing your child’s symptoms?
   - Yes
   - No
4. What do you think is the cause of your child’s problem with fatigue / energy?
   - Definitely physical
   - Mainly physical
   - Equally physical / psychological
   - Mainly psychological
   - Definitely psychological
   - Not having a problem with fatigue / energy
5. Has your child every been diagnosed or treated for any of the following conditions? If yes, please provide more details in the appropriate blank, including year of diagnosis and history of treatment.
   1. Major depressive disorder ________________________________________________________________________________________________________________________________________________
   2. Major depressive disorder with melancholic features ________________________________________________________________________________________________________________________________________________
   3. Bipolar disorder (manic-depression) ________________________________________________________________________________________________________________________________________________
   4. Anxiety ________________________________________________________________________________________________________________________________________________
   5. Schizophrenia ________________________________________________________________________________________________________________________________________________
   6. Eating disorder ________________________________________________________________________________________________________________________________________________
   7. Substance abuse ________________________________________________________________________________________________________________________________________________
   8. Multiple chemical sensitivities ________________________________________________________________________________________________________________________________________________
   9. Fibromyalgia ________________________________________________________________________________________________________________________________________________
   10. Allergies ________________________________________________________________________________________________________________________________________________
   11. Other ________________________________________________________________________________________________________________________________________________
   12. No diagnosis / treatment
