# Supplementary material for: The Development of the DePaul Symptom Questionnaire: Original, Expanded, Brief, and Pediatric Versions
Source: Front Pediatr. 2018 Nov 6;6:330. doi: 10.3389/fped.2018.00330 (PMC6232226; doi:10.3389/fped.2018.00330)
Supplement: Supplementary file 5 [file Data_Sheet_5.docx]

**Data Sheet 5.**

**DSQ Pediatric (DSQ-Ped)**

**Child Form**

This document contains the following material:

1. Case definition scoring rules and associated symptoms for the following criteria:
   1. Jason et al. (2006) Pediatric Case Definition for ME and CFS
   2. Rowe et al. (2017) Pediatric ME/CFS Case Definition
2. Syntax for the following case definitions:
   1. Jason et al. (2006) Pediatric Case Definition for ME and CFS
   2. Rowe et al. (2017) Pediatric ME/CFS Case Definition
3. Hard copy of the DSQ-Ped (Child Form)

The DSQ-Ped (Child Form) can be downloaded from the REDCap shared library. You can view the instrument here: <https://redcap.is.depaul.edu/surveys/?s=7N399W47JF>

**DSQ-Ped**

**(Child Form)**

**Case Definition Criteria:**

*Note: Results from both the parent and child forms of the DSQ-Ped should be considered when making diagnostic decisions.*

**Pediatric Case Definition for ME and CFS (Jason et al., 2006)**

- 3+ months Fatigue (Question 11; frequency and severity scores >= 2)
  - *Note: Due to challenges in measuring the ‘substantial reduction in functioning’ criterion among children, clinician assessment is required to determine if the child has demonstrated a significant reduction in educational, social, and/or personal activities*
- At least 1 symptom from each of the following domains (frequency and severity scores >= 2):
  - Post-exertional malaise (Questions 12-16)
  - Unrefreshing sleep (Questions 17-21)
  - Pain (Question 22-27; 38-41)
- At least 2 neurocognitive manifestations (Questions 28-37; frequency and severity scores >= 2)
- At least 1 symptom from 2 of the following categories (frequency and severity scores >= 2):
  - Autonomic (Questions 42-45)
  - Neuroendocrine (Questions 46-53)
  - Immune (Questions 54-57)

**Pediatric ME/CFS Criteria (Rowe et al., 2006)**

- 6+ months of illness (Question 64a >= 6)
- Impaired function; at least 1 of the following symptoms:
  - Question 71 (*“Yes”*)
  - Question 76 (“*Yes*”)
- Fatigue
  - Question 11 (frequency and severity scores >= 2)
  - Not relieved by rest: Question 65 (“*Some of it goes away*” or “*None of it goes away*”)
- Post-exertional symptoms (At least 1 symptom from Questions 12-16; frequency and severity scores >= 2)
- At least 1 symptom from 2 of the following categories (frequency and severity scores >= 2):
  - Sleep problems (Questions 17-21)
  - Cognitive problems (Questions 29-37)
  - Pain (Questions 22-23; 26-27; 54-55)

**DSQ-Ped (Child Form)**

**SPSS Case Definition Syntax:**

**Results from both the parent and child forms of the DSQ-Ped should be considered when making diagnostic decisions*.*

**Scoring Note: To determine which variables are associated with which items, note that variable names utilize the items numbers present in the questionnaire*.*

********************************************************************************

*DSQ-Ped (Child Form)*.

* Pediatric Case Definition for ME and CFS (Jason et al., 2006)*.

********************************************************************************.

*Fatigue*.

**Note: Due to challenges in measuring the ‘substantial reduction in functioning’ criterion among children, clinician assessment is required to determine if the child has demonstrated a significant reduction in educational, social, and/or personal activities*.*

COMPUTE PedC_Fatigue = 0.

EXECUTE.

IF((dsqp_c_11f >= 2) & (dsqp_c_11s >= 2)) PedC_Fatigue = 1.

EXECUTE.

*Post-exertional malaise*.

COMPUTE PedC_12 = 0.

EXECUTE.

IF((dsqp_c_12f >= 2) & (dsqp_c_12s >= 2)) PedC_12 = 1.

EXECUTE.

COMPUTE PedC_13 = 0.

EXECUTE.

IF((dsqp_c_13f >= 2) & (dsqp_c_13s >= 2)) PedC_13 = 1.

EXECUTE.

COMPUTE PedC_14 = 0.

EXECUTE.

IF((dsqp_c_14f >= 2) & (dsqp_c_14s >= 2)) PedC_14 = 1.

EXECUTE.

COMPUTE PedC_15 = 0.

EXECUTE.

IF((dsqp_c_15f >= 2) & (dsqp_c_15s >= 2)) PedC_15 = 1.

EXECUTE.

COMPUTE PedC_16 = 0.

EXECUTE.

IF((dsqp_c_16f >= 2) & (dsqp_c_16s >= 2)) PedC_16 = 1.

EXECUTE.

COMPUTE PedC_PEM = 0.

EXECUTE.

IF(SUM(PedC_12, PedC_13, PedC_14, PedC_15, PedC_16) >= 1) PedC_PEM = 1.

EXECUTE.

*Unrefreshing Sleep*.

COMPUTE PedC_17 = 0.

EXECUTE.

IF((dsqp_c_17f >= 2) & (dsqp_c_17s >= 2)) PedC_17 = 1.

EXECUTE.

COMPUTE PedC_18 = 0.

EXECUTE.

IF((dsqp_c_18f >= 2) & (dsqp_c_18s >= 2)) PedC_18 = 1.

EXECUTE.

COMPUTE PedC_19 = 0.

EXECUTE.

IF((dsqp_c_19f >= 2) & (dsqp_c_19s >= 2)) PedC_19 = 1.

EXECUTE.

COMPUTE PedC_20 = 0.

EXECUTE.

IF((dsqp_c_20f >= 2) & (dsqp_c_20s >= 2)) PedC_20 = 1.

EXECUTE.

COMPUTE PedC_21 = 0.

EXECUTE.

IF((dsqp_c_21f >= 2) & (dsqp_c_21s >= 2)) PedC_21 = 1.

EXECUTE.

COMPUTE PedC_Sleep = 0.

EXECUTE.

IF(SUM(PedC_17, PedC_18, PedC_19, PedC_20, PedC_21) >= 1) PedC_Sleep = 1.

EXECUTE.

*Pain*.

COMPUTE PedC_22 = 0.

EXECUTE.

IF((dsqp_c_22f >= 2) & (dsqp_c_22s >= 2)) PedC_22 = 1.

EXECUTE.

COMPUTE PedC_23 = 0.

EXECUTE.

IF((dsqp_c_23f >= 2) & (dsqp_c_23s >= 2)) PedC_23 = 1.

EXECUTE.

COMPUTE PedC_24 = 0.

EXECUTE.

IF((dsqp_c_24f >= 2) & (dsqp_c_24s >= 2)) PedC_24 = 1.

EXECUTE.

COMPUTE PedC_25 = 0.

EXECUTE.

IF((dsqp_c_25f >= 2) & (dsqp_c_25s >= 2)) PedC_25 = 1.

EXECUTE.

COMPUTE PedC_26 = 0.

EXECUTE.

IF((dsqp_c_26f >= 2) & (dsqp_c_26s >= 2)) PedC_26 = 1.

EXECUTE.

COMPUTE PedC_27 = 0.

EXECUTE.

IF((dsqp_c_27f >= 2) & (dsqp_c_27s >= 2)) PedC_27 = 1.

EXECUTE.

COMPUTE PedC_38 = 0.

EXECUTE.

IF((dsqp_c_38f >= 2) & (dsqp_c_38s >= 2)) PedC_38 = 1.

EXECUTE.

COMPUTE PedC_39 = 0.

EXECUTE.

IF((dsqp_c_39f >= 2) & (dsqp_c_39s >= 2)) PedC_39 = 1.

EXECUTE.

COMPUTE PedC_40 = 0.

EXECUTE.

IF((dsqp_c_40f >= 2) & (dsqp_c_40s >= 2)) PedC_40 = 1.

EXECUTE.

COMPUTE PedC_41 = 0.

EXECUTE.

IF((dsqp_c_41f >= 2) & (dsqp_c_41s >= 2)) PedC_41 = 1.

EXECUTE.

COMPUTE PedC_Pain = 0.

IF(SUM(PedC_22, PedC_23, PedC_24, PedC_25, PedC_26, PedC_27, PedC_38, PedC_39, PedC_40, PedC_41) >= 1) PedC_Pain = 1.

EXECUTE.

*Neurocognitive*.

COMPUTE PedC_28= 0.

EXECUTE.

IF((dsqp_c_28f >= 2) & (dsqp_c_28s >= 2)) PedC_28 = 1.

EXECUTE.

COMPUTE PedC_29 = 0.

EXECUTE.

IF((dsqp_c_29f >= 2) & (dsqp_c_29s >= 2)) PedC_29 = 1.

EXECUTE.

COMPUTE PedC_30 = 0.

EXECUTE.

IF((dsqp_c_30f >= 2) & (dsqp_c_30s >= 2)) PedC_30 = 1.

EXECUTE.

COMPUTE PedC_31 = 0.

EXECUTE.

IF((dsqp_c_31f >= 2) & (dsqp_c_31s >= 2)) PedC_31 = 1.

EXECUTE.

COMPUTE PedC_32 = 0.

EXECUTE.

IF((dsqp_c_32f >= 2) & (dsqp_c_32s >= 2)) PedC_32 = 1.

EXECUTE.

COMPUTE PedC_33 = 0.

EXECUTE.

IF((dsqp_c_33f >= 2) & (dsqp_c_33s >= 2)) PedC_33 = 1.

EXECUTE.

COMPUTE PedC_34 = 0.

EXECUTE.

IF((dsqp_c_34f >= 2) & (dsqp_c_34s >= 2)) PedC_34 = 1.

EXECUTE.

COMPUTE PedC_35 = 0.

EXECUTE.

IF((dsqp_c_35f >= 2) & (dsqp_c_35s >= 2)) PedC_35 = 1.

EXECUTE.

COMPUTE PedC_36 = 0.

EXECUTE.

IF((dsqp_c_36f >= 2) & (dsqp_c_36s >= 2)) PedC_36 = 1.

EXECUTE.

COMPUTE PedC_37 = 0.

EXECUTE.

IF((dsqp_c_37f >= 2) & (dsqp_c_37s >= 2)) PedC_37 = 1.

EXECUTE.

COMPUTE PedC_Cognitive = 0.

EXECUTE.

IF(SUM(PedC_28, PedC_29, PedC_30, PedC_31, PedC_32, PedC_33, PedC_34, PedC_35, PedC_36, PedC_27) >= 2) PedC_Cognitive = 1.

EXECUTE.

*Autonomic*.

COMPUTE PedC_42 = 0.

EXECUTE.

IF((dsqp_c_42f >= 2) & (dsqp_c_42s >= 2)) PedC_42 = 1.

EXECUTE.

COMPUTE PedC_43 = 0.

EXECUTE.

IF((dsqp_c_43f >= 2) & (dsqp_c_43s >= 2)) PedC_43 = 1.

EXECUTE.

COMPUTE PedC_44 = 0.

EXECUTE.

IF((dsqp_c_44f >= 2) & (dsqp_c_44s >= 2)) PedC_44 = 1.

EXECUTE.

COMPUTE PedC_45 = 0.

EXECUTE.

IF((dsqp_c_45f >= 2) & (dsqp_c_45s >= 2)) PedC_45 = 1.

EXECUTE.

COMPUTE PedC_Autonomic = 0.

EXECUTE.

IF(SUM(PedC_42, PedC_43, PedC_44, PedC_45) >= 1) PedC_Autonomic = 1.

EXECUTE.

*Neuroendocrine*.

COMPUTE PedC_46 = 0.

EXECUTE.

IF((dsqp_c_46f >= 2) & (dsqp_c_46s >= 2)) PedC_46 = 1.

EXECUTE.

COMPUTE PedC_47 = 0.

EXECUTE.

IF((dsqp_c_47f >= 2) & (dsqp_c_47s >= 2)) PedC_47 = 1.

EXECUTE.

COMPUTE PedC_48 = 0.

EXECUTE.

IF((dsqp_c_48f >= 2) & (dsqp_c_48s >= 2)) PedC_48 = 1.

EXECUTE.

COMPUTE PedC_49 = 0.

EXECUTE.

IF((dsqp_c_49f >= 2) & (dsqp_c_49s >= 2)) PedC_49 = 1.

EXECUTE.

COMPUTE PedC_50 = 0.

EXECUTE.

IF((dsqp_c_50f >= 2) & (dsqp_c_50s >= 2)) PedC_50 = 1.

EXECUTE.

COMPUTE PedC_51 = 0.

EXECUTE.

IF((dsqp_c_51f >= 2) & (dsqp_c_51s >= 2)) PedC_51 = 1.

EXECUTE.

COMPUTE PedC_52 = 0.

EXECUTE.

IF((dsqp_c_52f >= 2) & (dsqp_c_52s >= 2)) PedC_52 = 1.

EXECUTE.

COMPUTE PedC_53 = 0.

EXECUTE.

IF((dsqp_c_53f >= 2) & (dsqp_c_53s >= 2)) PedC_53 = 1.

EXECUTE.

COMPUTE PedC_Neuroendo = 0.

EXECUTE.

IF(SUM(PedC_46, PedC_47, PedC_48, PedC_49, PedC_50, PedC_51, PedC_52, PedC_53) >= 1) PedC_Neuroendo = 1.

EXECUTE.

*Immune*

COMPUTE PedC_54 = 0.

EXECUTE.

IF((dsqp_c_54f >= 2) & (dsqp_c_54s >= 2)) PedC_54 = 1.

EXECUTE.

COMPUTE PedC_55 = 0.

EXECUTE.

IF((dsqp_c_55f >= 2) & (dsqp_c_55s >= 2)) PedC_55 = 1.

EXECUTE.

COMPUTE PedC_56 = 0.

EXECUTE.

IF((dsqp_c_56f >= 2) & (dsqp_c_56s >= 2)) PedC_56 = 1.

EXECUTE.

COMPUTE PedC_57 = 0.

EXECUTE.

IF((dsqp_c_57f >= 2) & (dsqp_c_57s >= 2)) PedC_57 = 1.

EXECUTE.

COMPUTE PedC_Immune = 0.

EXECUTE.

IF(SUM(PedC_54, PedC_55, PedC_56, PedC_57) >= 1) PedC_Immune = 1.

EXECUTE.

***Jason et al. (2006) Pediatric Case Definition for ME and CFS***.

COMPUTE PedC_ANI2 = 0.

EXECUTE.

IF(SUM(PedC_Autonomic, PedC_Neuroendo, PedC_Immune) >= 2) PedC_ANI2 = 1.

EXECUTE.

COMPUTE PedC_Jason = 0.

EXECUTE.

IF(SUM(PedC_Fatigue, PedC_PEM, PedC_Sleep, PedC_Pain, PedC_Cognitive, PedC_ANI2) = 6) PedC_Jason = 1.

EXECUTE.

VALUE LABELS

PedC_Jason

0 ‘Does not meet Jason et al Pediatric ME and CFS Criteria’

1 ‘Meets Jason et al Pediatric ME and CFS Criteria’.

EXECUTE.

********************************************************************************.

*DSQ-Ped (Child Form)*.

* Pediatric ME/CFS Criteria (Rowe et al., 2017)*.

********************************************************************************.

*Six months of illness*.

COMPUTE RoweC_Six = 0.

EXECUTE.

IF(dsqp_c_64a >= 6) RoweC_Six = 1.

EXECUTE.

*Impaired Function*.

COMPUTE RoweC_71 = 0.

EXECUTE.

IF(dsqp_c_71 = 1) RoweC_71 = 1.

EXECUTE.

COMPUTE RoweC_76 = 0.

EXECUTE.

IF(dsqp_c_76 = 1) RoweC_76 = 1.

EXECUTE.

COMPUTE RoweC_Function = 0.

EXECUTE.

IF((RoweC_71 = 1) | (RoweC_76 = 1)) RoweC_Function = 1.

EXECUTE.

*Fatigue*.

COMPUTE RoweC_11 = 0.

EXECUTE.

IF((dsqp_c_11f >= 2) & (dsqp_c_11s >= 2)) RoweC_11 = 1.

EXECUTE.

COMPUTE RoweC_65 = 0.

EXECUTE.

IF((dsqp_c_65 = 2) | (dsqp_c_65 = 3)) RoweC_65 = 1.

EXECUTE.

COMPUTE RoweC_Fatigue = 0.

EXECUTE.

IF(SUM(RoweC_11, RoweC_65) = 2) RoweC_Fatigue = 1.

EXECUTE.

*Post-exertional malaise*.

COMPUTE RoweC_12 = 0.

EXECUTE.

IF((dsqp_c_12f >= 2) & (dsqp_c_12s >= 2)) RoweC_12 = 1.

EXECUTE.

COMPUTE RoweC_13 = 0.

EXECUTE.

IF((dsqp_c_13f >= 2) & (dsqp_c_13s >= 2)) RoweC_13 = 1.

EXECUTE.

COMPUTE RoweC_14 = 0.

EXECUTE.

IF((dsqp_c_14f >= 2) & (dsqp_c_14s >= 2)) RoweC_14 = 1.

EXECUTE.

COMPUTE RoweC_15 = 0.

EXECUTE.

IF((dsqp_c_15f >= 2) & (dsqp_c_15s >= 2)) RoweC_15 = 1.

EXECUTE.

COMPUTE RoweC_16 = 0.

EXECUTE.

IF((dsqp_c_16f >= 2) & (dsqp_c_16s >= 2)) RoweC_16 = 1.

EXECUTE.

COMPUTE RoweC_PEM = 0.

EXECUTE.

IF(SUM(RoweC_12, RoweC_13, RoweC_14, RoweC_15, RoweC_16) >= 1) RoweC_PEM = 1.

EXECUTE.

*Sleep Problems*.

COMPUTE RoweC_17 = 0.

EXECUTE.

IF((dsqp_c_17f >= 2) & (dsqp_c_17s >= 2)) RoweC_17 = 1.

EXECUTE.

COMPUTE RoweC_18 = 0.

EXECUTE.

IF((dsqp_c_18f >= 2) & (dsqp_c_18s >= 2)) RoweC_18 = 1.

EXECUTE.

COMPUTE RoweC_19 = 0.

EXECUTE.

IF((dsqp_c_19f >= 2) & (dsqp_c_19s >= 2)) RoweC_19 = 1.

EXECUTE.

COMPUTE RoweC_20 = 0.

EXECUTE.

IF((dsqp_c_20f >= 2) & (dsqp_c_20s >= 2)) RoweC_20 = 1.

EXECUTE.

COMPUTE RoweC_21 = 0.

EXECUTE.

IF((dsqp_c_21f >= 2) & (dsqp_c_21s >= 2)) RoweC_21 = 1.

EXECUTE.

COMPUTE RoweC_Sleep = 0.

EXECUTE.

IF(SUM(RoweC_17, RoweC_18, RoweC_19, RoweC_20, RoweC_21) >= 1) RoweC_Sleep = 1.

EXECUTE.

*Cognitive Problems*.

COMPUTE RoweC_29 = 0.

EXECUTE.

IF((dsqp_c_29f >= 2) & (dsqp_c_29s >= 2)) RoweC_29 = 1.

EXECUTE.

COMPUTE RoweC_30 = 0.

EXECUTE.

IF((dsqp_c_30f >= 2) & (dsqp_c_30s >= 2)) RoweC_30 = 1.

EXECUTE.

COMPUTE RoweC_31 = 0.

EXECUTE.

IF((dsqp_c_31f >= 2) & (dsqp_c_31s >= 2)) RoweC_31 = 1.

EXECUTE.

COMPUTE RoweC_32 = 0.

EXECUTE.

IF((dsqp_c_32f >= 2) & (dsqp_c_32s >= 2)) RoweC_32 = 1.

EXECUTE.

COMPUTE RoweC_33 = 0.

EXECUTE.

IF((dsqp_c_33f >= 2) & (dsqp_c_33s >= 2)) RoweC_33 = 1.

EXECUTE.

COMPUTE RoweC_34 = 0.

EXECUTE.

IF((dsqp_c_34f >= 2) & (dsqp_c_34s >= 2)) RoweC_34 = 1.

EXECUTE.

COMPUTE RoweC_35 = 0.

EXECUTE.

IF((dsqp_c_35f >= 2) & (dsqp_c_35s >= 2)) RoweC_35 = 1.

EXECUTE.

COMPUTE RoweC_36 = 0.

EXECUTE.

IF((dsqp_c_36f >= 2) & (dsqp_c_36s >= 2)) RoweC_36 = 1.

EXECUTE.

COMPUTE RoweC_37 = 0.

EXECUTE.

IF((dsqp_c_37f >= 2) & (dsqp_c_37s >= 2)) RoweC_37 = 1.

EXECUTE.

COMPUTE RoweC_Cognitive = 0.

EXECUTE.

IF(SUM(RoweC_29, RoweC_30, RoweC_31, RoweC_32, RoweC_33, RoweC_34, RoweC_35, RoweC_36, RoweC_37) >= 1) RoweC_Cognitive = 1.

EXECUTE.

*Pain*.

COMPUTE RoweC_22 = 0.

EXECUTE.

IF((dsqp_c_22f >= 2) & (dsqp_c_22s >= 2)) RoweC_22 = 1.

EXECUTE.

COMPUTE RoweC_23 = 0.

EXECUTE.

IF((dsqp_c_23f >= 2) & (dsqp_c_23s >= 2)) RoweC_23 = 1.

EXECUTE.

COMPUTE RoweC_26 = 0.

EXECUTE.

IF((dsqp_c_26f >= 2) & (dsqp_c_26s >= 2)) RoweC_26 = 1.

EXECUTE.

COMPUTE RoweC_27 = 0.

EXECUTE.

IF((dsqp_c_27f >= 2) & (dsqp_c_27s >= 2)) RoweC_27 = 1.

EXECUTE.

COMPUTE RoweC_54 = 0.

EXECUTE.

IF((dsqp_c_54f >= 2) & (dsqp_c_54s >= 2)) RoweC_54 = 1.

EXECUTE.

COMPUTE RoweC_55 = 0.

EXECUTE.

IF((dsqp_c_55f >= 2) & (dsqp_c_55s >= 2)) RoweC_55 = 1.

EXECUTE.

COMPUTE RoweC_Pain = 0.

EXECUTE.

IF(SUM(RoweC_22, RoweC_23, RoweC_26, RoweC_27, RoweC_54, RoweC_55) >= 1) RoweC_Pain = 1.

EXECUTE.

***Pediatric ME/CFS Criteria (Rowe et al., 2017)***.

COMPUTE RoweC_SCP2 = 0.

EXECUTE.

IF(SUM(RoweC_Sleep, RoweC_Cognitive, RoweC_Pain) >= 2) RoweC_SCP2 = 1.

EXECUTE.

COMPUTE Rowe_Child = 0.

EXECUTE.

IF(SUM(RoweC_Six, RoweC_Function, RoweC_Fatigue, RoweC_PEM, RoweC_SCP2) = 5) Rowe_Child = 1.

EXECUTE.

VALUE LABELS

Rowe_Child

0 ‘ Does Not Meet Rowe et al Pediatric MECFS Criteria’

1 ‘Meets Rowe et al Pediatric MECFS Criteria’.

EXECUTE.

**DePaul Symptom Questionnaire – Pediatric (Child Report Form)**

**DSQ-Ped (Child)**

**Note:** *Children under the age of 12, or those with reading or comprehension difficulties, should complete this questionnaire with the assistance of a parent or guardian.*

Participant ID: _______________

1. Today’s Date: _______________
2. Date of Birth: _______________
3. Age (in years): _______________
4. Gender:
   - Male
   - Female
   - Other
   - Prefer not to respond
5. What grade are you in right now at school?
   - Preschool
   - Kindergarten
   - 1^st^ Grade
   - 2^nd^ Grade
   - 3^rd^ Grade
   - 4^th^ Grade
   - 5^th^ Grade
   - 6^th^ Grade
   - 7^th^ Grade
   - 8^th^ Grade
   - 9^th^ Grade (Freshman)
   - 10^th^ Grade (Sophomore)
   - 11^th^ Grade (Junior)
   - 12^th^ Grade (Senior)
   - Ungraded
     1. If ungraded, for how many years have you attended school? _______________
6. Do you have any siblings?
   - Yes
   - No
     1. If yes, how many siblings do you have? _______________
     2. How many of your siblings are under 18 years old? _______________
7. How many people live in your home (including you)? _______________
8. What is your race? Select all that apply.
   - American Indian or Alaskan Native
   - Asian or Pacific Islander
   - Black / African American
   - White / Caucasian
   - Prefer not to respond
   - Other (please specify)
     1. If other, please specify your race: _______________
9. Are you of Latino or Hispanic origin?
   - Yes
   - No
10. What is your religious affiliation?
    - Atheist or Agnostic
    - Buddhist
    - Christian
    - Hindu
    - Jewish
    - Muslim
    - Prefer not to respond
    - Other (please specify)
11. If other, please specify your religion: _______________

For the next questions, think about how you have felt over the **past 3 months**. Please tell us how often you have had each symptom (frequency) and how much each symptom has bothered you (severity).

Please circle **one number for frequency** and **one number for severity**, using the following numbers:

| *Frequency:*  Throughout the **past 3 months**,  **how often** have you had this symptom?  For each symptom listed below, circle a number from:  **0 = none of the time**  **1 = hardly ever**  **2 = about half the time**  **3 = most of the time**  **4 = always** | *Severity:*  Throughout the **past 3 months**,  **how much** has this symptom bothered you?  For each symptom listed below, circle a number from:  **0 = no problem**  **1 = small problem**  **2 = medium problem**  **3 = big problem**  **4 = very big problem** |
| --- | --- |

| **Symptom** | **Frequency:** | **Severity:** |
| --- | --- | --- |
| 11. Fatigue / Extreme tiredness | 0 1 2 3 4 | 0 1 2 3 4 |
| 12. Your body feels heavy after starting to exercise | 0 1 2 3 4 | 0 1 2 3 4 |
| 13. Feeling sore or very tired after everyday activities, like walking around your house | 0 1 2 3 4 | 0 1 2 3 4 |
| 14. Your mind is tired after just a little effort | 0 1 2 3 4 | 0 1 2 3 4 |
| 15. A little bit of exercise makes your body tired | 0 1 2 3 4 | 0 1 2 3 4 |
| 16. Your body is tired or you feels sick after a little bit of activity | 0 1 2 3 4 | 0 1 2 3 4 |
| 17. Feeling tired after you wake up in the morning | 0 1 2 3 4 | 0 1 2 3 4 |
| 18. Need to nap daily | 0 1 2 3 4 | 0 1 2 3 4 |
| 19. Problems falling asleep | 0 1 2 3 4 | 0 1 2 3 4 |
| 20. Problems staying asleep | 0 1 2 3 4 | 0 1 2 3 4 |
| 21. Waking up early in the morning (like 3:00 am) | 0 1 2 3 4 | 0 1 2 3 4 |
| 22. Pain or aching in your muscles | 0 1 2 3 4 | 0 1 2 3 4 |
| 23. Pain / Stiffness / Tenderness in more than one joint, without swelling or redness | 0 1 2 3 4 | 0 1 2 3 4 |
| 24. Eye pain | 0 1 2 3 4 | 0 1 2 3 4 |
| 25. Chest pain / Heartburn | 0 1 2 3 4 | 0 1 2 3 4 |
| 26. Abdomen / Stomach pain | 0 1 2 3 4 | 0 1 2 3 4 |
| 27. Headaches | 0 1 2 3 4 | 0 1 2 3 4 |
| 28. Muscle twitches | 0 1 2 3 4 | 0 1 2 3 4 |
| **Symptom** | **Frequency:** | **Severity:** |
| 29. Problems remembering things | 0 1 2 3 4 | 0 1 2 3 4 |
| 30. Difficulty paying attention for a long period of time | 0 1 2 3 4 | 0 1 2 3 4 |
| 31. Difficulty finding the right word to say | 0 1 2 3 4 | 0 1 2 3 4 |
| 32. Difficulty understanding things | 0 1 2 3 4 | 0 1 2 3 4 |
| 33. Only able to focus on one thing at a time | 0 1 2 3 4 | 0 1 2 3 4 |
| 34. Slowness of thought | 0 1 2 3 4 | 0 1 2 3 4 |
| 35. Absent-mindedness or forgetfulness | 0 1 2 3 4 | 0 1 2 3 4 |
| 36. Frequently losing your train of thought | 0 1 2 3 4 | 0 1 2 3 4 |
| 37. Trouble with math or numbers | 0 1 2 3 4 | 0 1 2 3 4 |
| 38. Nausea | 0 1 2 3 4 | 0 1 2 3 4 |
| 39. Upset stomach | 0 1 2 3 4 | 0 1 2 3 4 |
| 40. Vomiting | 0 1 2 3 4 | 0 1 2 3 4 |
| 41. Ringing in ears | 0 1 2 3 4 | 0 1 2 3 4 |
| 42. Feeling unsteady on your feet, like you might fall | 0 1 2 3 4 | 0 1 2 3 4 |
| 43. Shortness of breath or trouble catching your breath | 0 1 2 3 4 | 0 1 2 3 4 |
| 44. Dizziness | 0 1 2 3 4 | 0 1 2 3 4 |
| 45. Irregular heart beats | 0 1 2 3 4 | 0 1 2 3 4 |
| 46. Losing or gaining weight without trying | 0 1 2 3 4 | 0 1 2 3 4 |
| 47. Sweating hands | 0 1 2 3 4 | 0 1 2 3 4 |
| 48. Not wanting to eat | 0 1 2 3 4 | 0 1 2 3 4 |
| 49. Night sweats | 0 1 2 3 4 | 0 1 2 3 4 |
| 50. Feeling chills or shivers | 0 1 2 3 4 | 0 1 2 3 4 |
| 51. Feeling hot or cold for no reason | 0 1 2 3 4 | 0 1 2 3 4 |
| 52. Feeling like you have a high temperature | 0 1 2 3 4 | 0 1 2 3 4 |
| 53. Feeling like you have a low temperature | 0 1 2 3 4 | 0 1 2 3 4 |
| 54. Sore throat | 0 1 2 3 4 | 0 1 2 3 4 |
| 55. Tender / Sore lymph nodes | 0 1 2 3 4 | 0 1 2 3 4 |
| 56. Fever and sweats | 0 1 2 3 4 | 0 1 2 3 4 |
| 57. Some smells, foods, or chemicals make you feel sick | 0 1 2 3 4 | 0 1 2 3 4 |
| 58. Rashes | 0 1 2 3 4 | 0 1 2 3 4 |
| 59. Allergies | 0 1 2 3 4 | 0 1 2 3 4 |

1. When you **first became sick**, what were your worst three symptoms? Please select from the list above, or if you are not sick, write “I am not ill.”
   1. Worst symptom: _________________________
   2. Second-worst symptom: _________________________
   3. Third-worst symptom: _________________________
2. **Right now**, what are your worst three symptoms? Please select from the list above, or if you are not sick, write “I am not ill.”
3. Worst symptom: _________________________
4. Second-worst symptom: _________________________
5. Third-worst symptom: _________________________
6. How long did it take for your problem with fatigue or tiredness to get started?
   - Quickly - within 24 hours
   - Over 1 week
   - Over 1 month
   - Over 2-3 months
   - Over 4-6 months
   - Over 7-11 months
   - Over 1-2 years
   - Longer than 2 years
   - I have always experienced fatigue
   - I do not have fatigue
7. If you have headaches now, do you get them more often, in a different place, or do the headaches feel worse than they did in the past?
   - Headaches happen more often
   - Headaches feel worse / more severe
   - Headaches are in a different place / spot
   - I don’t have headaches
8. Have you been experiencing problems with fatigue / extreme tiredness for **at least one month**?
   - Yes
   - No
9. If yes, for about how many months? _______________
10. If you rest, does all of your fatigue go away, some of it go away, or none of it go away?
    - All of it goes away
    - Some of it goes away
    - None of it goes away
    - I do not have fatigue
11. If some or all of it goes away, for **how many hours** do you have to rest before your fatigue gets better? _______________
12. Does you participate in any hobbies or activities outside of school?
    - Yes
    - No
    1. If no, why? Select all that apply.
       - Not interested
       - No time
       - Would like to, but cannot because of problems with fatigue / energy
       - Cannot because hobbies or activities make symptoms worse
13. Do you limit or cut back activity levels to avoid feeling even more tired?
    - Yes
    - No
14. How would you describe the way your fatigue problem is changing over time?
    - My fatigue is getting worse
    - I have good and bad times
    - There is no change
    - My fatigue is getting better
    - I do not have fatigue
15. Do your symptoms change over time?
    - Yes
    - No
16. Did your fatigue or energy problem start after you experienced any of the following? Check all that apply. If you check something, please tell us more about what happened and how old you were when it happened.
    - - a. An infectious illness ________________________________________________________________________________________________________________________________________________
    - b. An accident ________________________________________________________________________________________________________________________________________________
    - c. A trip or vacation ________________________________________________________________________________________________________________________________________________
    - d. An immunization (shot at doctor’s office) ________________________________________________________________________________________________________________________________________________
    - e. Surgery ________________________________________________________________________________________________________________________________________________
    - f. Severe stress (bad or unhappy events) ________________________________________________________________________________________________________________________________________________
    - g. Other (please describe below) ________________________________________________________________________________________________________________________________________________
17. Since the start of your problems with fatigue or energy, have your symptoms caused you to reduce the time you do activities by 50% or more?
    - Yes
    - No
    - Not having a problem with fatigue or energy
18. Do you seem to catch illnesses (get sick) more easily than other people your age?
    1. Yes
    2. No
19. Does it seem to take you longer to get better after you are sick than other people your age?
    1. Yes
    2. No
20. Are you more uncomfortable than other people your age when it is extremely hot or cold?
    1. Yes
    2. No
21. Thinking about the past month, about how many **hours per week** in a typical week have you spent on:
    1. School / School activities _______________
    2. Sports / Recreational activities _______________
    3. Activities with friends and family _______________
    4. Work activities _______________
22. In the past 4 weeks, have you had to reduce the number of hours you used to spend (before your illness) on school, sports or recreational activities, activities with friends or family, or work because of your health or problems with fatigue / energy?
    - Yes
    - No
    - Not having problems with fatigue / energy

If you answered “Yes” to the previous question: **before** your fatigue/energy-related problem, about how many hours per week did you spend on:

- 1. School / School activities _______________
  2. Sports / Recreational activities _______________
  3. Activities with friends and family _______________
  4. Work activities _______________

1. In a normal week, please rate the amount of **energy you have available** using a scale from 1 to 100, where 1 = no energy and 100 = a lot of energy: _______________
2. In a normal week, please rate the amount of **energy you *use*** on a scale from 1 to 100, where 1 = no energy and 100 = a lot of energy: _______________
3. In a normal week, please rate the amount of **fatigue** you have using a scale from 1 to 100, where 1 = no fatigue and 100 = very bad fatigue: _______________
