# Supplementary material for: The Development of the DePaul Symptom Questionnaire: Original, Expanded, Brief, and Pediatric Versions
Source: Front Pediatr. 2018 Nov 6;6:330. doi: 10.3389/fped.2018.00330 (PMC6232226; doi:10.3389/fped.2018.00330)
Supplement: Supplementary file 6 [file Data_Sheet_6.docx]

**Data Sheet 6.**

**DSQ Pediatric Screening Questionnaire (DSQ-PSQ)**

This document contains the following material:

1. Scoring rules to identify children who “screen positive” for symptoms of ME and CFS
2. Syntax for the “screen positive” criteria
3. Hard copy of the DSQ-PSQ

The DSQ Pediatric Screening Questionnaire (DSQ-PSQ) can be downloaded from the REDCap shared library. You can view the instrument here: <https://redcap.is.depaul.edu/surveys/?s=MFF8TXRPC8>

**DSQ-PSQ**

**Screen Positive Criteria**

**Screen Positive Criteria** *(to identify children who may have symptoms of ME and CFS)*:

- At least 1 of the following symptoms:
  - Fatigue / Extreme tiredness (Question 6; frequency and severity scores >= 2)
  - Missing activities because child is too sick/tired (Question 7, “Yes”)
  - Poor school attendance (Question 8, “Yes”)
  - Unable/Unwilling to go to school (Question 9, “Yes”)
  - School learning or memory problems (Question 10, “Yes”)
- A response of “Yes” to at least 4 of the following symptoms:
  - Headaches (Question 11)
  - Sore throat (Question 12)
  - Joint pain (Question 13)
  - Muscle pain (Question 14)
  - Abdominal pain (Question 15)
  - Lymph node pain (Question 16)
  - Rash (Question 17)
  - Fever, chills, or shivers (Question 18)
  - Eye pain / Light sensitivity (Question 19)
  - Problems sleeping (Question 20)
  - Impaired memory or concentration (Question 21)
  - Feeling worse/sick/exhausted after exercise (Question 22)
  - Dizziness (Question 23)

**DSQ-PSQ**

**SPSS Syntax:**

**Scoring Note: To determine which variables are associated with which items, note that variable names utilize the items numbers present in the questionnaire*.*

*****************************************************************************.

*DSQ-PSQ*.

*Screen Positive Criteria*.

*****************************************************************************.

*Criterion 1.*

*Fatigue or School Problems*.

COMPUTE SP_6 = 0.

EXECUTE.

IF((psq_6f >= 2) & (psq_6s >=2)) SP_6 = 1.

EXECUTE.

COMPUTE SP_7 = 0.

EXECUTE.

IF(psq_7 = 1) SP_7 = 1.

EXECUTE.

COMPUTE SP_8 = 0.

EXECUTE.

IF(psq_8 = 1) SP_8 = 1.

EXECUTE.

COMPUTE SP_9 = 0.

EXECUTE.

IF(psq_9 = 1) SP_9 = 1.

EXECUTE.

COMPUTE SP_10 = 0.

EXECUTE.

IF(psq_10 = 1) SP_10 = 1.

EXECUTE.

COMPUTE PSQ_SP1 = 0.

EXECUTE.

IF(SUM(SP_6, SP_7, SP_8, SP_9, SP_10) >= 1) PSQ_SP1 = 1.

EXECUTE.

*Criterion 2.*

*At least 4 ME or CFS symptoms*.

COMPUTE SP_11 = 0.

EXECUTE.

IF(psq_11 = 1) SP_11 = 1.

EXECUTE.

COMPUTE SP_12 = 0.

EXECUTE.

IF(psq_12 = 1) SP_12 = 1.

EXECUTE.

COMPUTE SP_13 = 0.

EXECUTE.

IF(psq_13 = 1) SP_13 = 1.

EXECUTE.

COMPUTE SP_14 = 0.

EXECUTE.

IF(psq_14 = 1) SP_14 = 1.

EXECUTE.

COMPUTE SP_15 = 0.

EXECUTE.

IF(psq_15 = 1) SP_15 = 1.

EXECUTE.

COMPUTE SP_16 = 0.

EXECUTE.

IF(psq_16 = 1) SP_16 = 1.

EXECUTE.

COMPUTE SP_17 = 0.

EXECUTE.

IF(psq_17 = 1) SP_17 = 1.

EXECUTE.

COMPUTE SP_18 = 0.

EXECUTE.

IF(psq_18 = 1) SP_18 = 1.

EXECUTE.

COMPUTE SP_19 = 0.

EXECUTE.

IF(psq_19 = 1) SP_19 = 1.

EXECUTE.

COMPUTE SP_20 = 0.

EXECUTE.

IF(psq_20 = 1) SP_20 = 1.

EXECUTE.

COMPUTE SP_21 = 0.

EXECUTE.

IF(psq_21 = 1) SP_21 = 1.

EXECUTE.

COMPUTE SP_22 = 0.

EXECUTE.

IF(psq_22 = 1) SP_22 = 1.

EXECUTE.

COMPUTE SP_23 = 0.

EXECUTE.

IF(psq_23 = 1) SP_23 = 1.

EXECUTE.

COMPUTE PSQ_SP2 = 0.

EXECUTE.

IF(SUM(SP_11, SP_12, SP_13, SP_14, SP_15, SP_16, SP_17, SP_18, SP_19, SP_20, SP_21, SP_22, SP_23) >= 4) PSQ_SP2 = 1.

***Screen Positive Criteria***.

COMPUTE PSQ_Screen = 0.

EXECUTE.

IF((PSQ_SP1 = 1) & (PSQ_SP2) = 1) PSQ_Screen = 1.

EXECUTE.

VALUE LABELS

PSQ_Screen

0 ‘Screen Negative

1 ‘Screen Positive’.

EXECUTE.

**DePaul Symptom Questionnaire – Pediatric Screening Questionnaire**

**DSQ – PSQ**

Date: _______________

Participant ID: _______________

1. Child’s Initials: _______________
2. Child’s Age: _______________
3. Child’s Gender:

- Female
- Male
- Other
- Prefer not to answer

1. Child’s Race (select all that apply):

- Black, African American
- White, Caucasian
- American Indian or Alaskan Native
- Asian
- Native Hawaiian or Pacific Islander
- Other

1. If other, please specify: _______________
2. Is your child of Latino or Hispanic origin?

- Yes
- No

**DSQ – PSQ (Continued)**

Please indicate if your child has had problems with any of the following over the past

**3 to 6 months** by circling “Yes” or “No.”

For all items marked “Yes”, circle **one number for frequency** and **one number for severity**, using the following scales:

| *Frequency:*  Throughout the **past 3 to 6 months**,  **how often** has your child had this symptom?  For each symptom listed below, circle a number from:  **0 = none of the time**  **1 = a little of the time**  **2 = about half the time**  **3 = most of the time**  **4 = all of the time** | *Severity:*  Throughout the **past 3 to 6 months**,  **how much** has this symptom bothered your child?  For each symptom listed below, circle a number from:  **0 = symptom not present**  **1 = mild**  **2 = moderate**  **3 = severe**  **4 = very severe** |
| --- | --- |

|  |  | *Only for items marked “****Yes****:”* | |
| --- | --- | --- | --- |
| **Symptom** |  | ***Frequency:*** | ***Severity:*** |
| 6. Fatigue / Extreme tiredness | Yes No | *0 1 2 3 4* | *0 1 2 3 4* |
| 1. Has your child had a problem with fatigue or energy for **6 months or longer**? | Yes No | *--* | *--* |
| 7. Missing activities because he/she is too sick or too tired | Yes No | *0 1 2 3 4* | *0 1 2 3 4* |
| 8. Poor school attendance | Yes No | *0 1 2 3 4* | *0 1 2 3 4* |
| 9. Being unable or unwilling to go to school | Yes No | *0 1 2 3 4* | *0 1 2 3 4* |
| 10. School learning or memory problems | Yes No | *0 1 2 3 4* | *0 1 2 3 4* |

**DSQ – PSQ (Continued)**

Please indicate if your child has had any of the following symptoms **constantly or repeatedly** over the past **3 to 6 months** by circling “Yes” or “No.”

For all items marked “Yes”, circle **one number for frequency** and **one number for severity**, using the following scales:

| *Frequency:*  Throughout the **past 3 to 6 months**,  **how often** has your child had this symptom?  For each symptom listed below, circle a number from:  **0 = none of the time**  **1 = a little of the time**  **2 = about half the time**  **3 = most of the time**  **4 = all of the time** | *Severity:*  Throughout the **past 3 to 6 months**,  **how much** has this symptom bothered your child?  For each symptom listed below, circle a number from:  **0 = symptom not present**  **1 = mild**  **2 = moderate**  **3 = severe**  **4 = very severe** |
| --- | --- |

|  |  | *Only for items marked “****Yes****:”* | |
| --- | --- | --- | --- |
| **Symptom** |  | ***Frequency:*** | ***Severity:*** |
| 11. Frequent headaches | Yes No | *0 1 2 3 4* | *0 1 2 3 4* |
| 12. Sore throat | Yes No | *0 1 2 3 4* | *0 1 2 3 4* |
| 13. Joint pain | Yes No | *0 1 2 3 4* | *0 1 2 3 4* |
| 14. Muscle pain | Yes No | *0 1 2 3 4* | *0 1 2 3 4* |
| 15. Abdominal pain | Yes No | *0 1 2 3 4* | *0 1 2 3 4* |
| 16. Lymph node pain (in neck or under arms) | Yes No | *0 1 2 3 4* | *0 1 2 3 4* |
| 17. Rash | Yes No | *0 1 2 3 4* | *0 1 2 3 4* |
| 18. Fever, chills, or shivers | Yes No | *0 1 2 3 4* | *0 1 2 3 4* |
| 19. Eye pain or light sensitivity | Yes No | *0 1 2 3 4* | *0 1 2 3 4* |
| 20. Problems sleeping | Yes No | *0 1 2 3 4* | *0 1 2 3 4* |
| 21. Impaired memory or concentration | Yes No | *0 1 2 3 4* | *0 1 2 3 4* |
| 22. Feeling worse, sick, or exhausted after exercise | Yes No | *0 1 2 3 4* | *0 1 2 3 4* |
| 23. Dizziness | Yes No | *0 1 2 3 4* | *0 1 2 3 4* |
